# Fourth International Bemisia Workshop International Whitefly Genomics Workshop December 3–8, 2006, Duck Key, Florida, USA

**DOI:** 10.1673/031.008.0401

**Published:** 2008-01-10

**Authors:** Phillip A. Stansly, Cindy L. McKenzie

**Affiliations:** ^1^ University of Florida/FAS, Southwest Florida REC, Immokalee, Florida, pstansly@ufl.edu; ^2^ USDA/ARS US Horticultural Research Laboratory, Fort Pierce, Florida. cmckenzie@ushrl.ars.usda.gov

Recently, the sycamore whitefly, *Bemisia afer* (Priesner & Hosny) has become a new economic pest and it attacks different economic plants in Egypt. The present work deals with host plants, geographical distribution and natural enemies of this pest. The results indicated that this species attacked 18 host plants distributed in 34 locations in Egypt. It was associated with 4 parasitoids [namely: *Encarsia inaron* (Walker), *Encarsia lutea* (Masi), *Eretmocerus* sp. and *Eretmocerus aegypticus* Evans and Abd-Rabou (Hymenoptera : Aphelinidae)] and 6 predators [namely: *Campylomma nicolasi* (Reuter) (Hemiptera: Miridae), *Chrysopa cornea* (Stephens) (Neuroptera: Chrysopidae), *Coccinella septempunctata* (L.) (Coleoptera: Coccinellidae), *Coccinella undecimpunctata* L. (Coleoptera: Coccinellidae), Orius sp. (Hemiptera Anthocoridae) and *Geocoris* sp. (Hemiptera: Lygaeidae)]. During this work, cotton (*Gossypium barbadense*) was recorded as a new host plant of economic importance attacked by *B. afer* and *E. aegypticus* as a promising natural enemy for controlling this pest in Egypt.

