# Supplementary material for: Fourth International Bemisia Workshop International Whitefly Genomics Workshop December 3–8, 2006, Duck Key, Florida, USA
Source: J Insect Sci. 2008 Jan 10;8:4. doi: 10.1673/031.008.0401 (PMC3061584; doi:10.1673/031.008.0401)
Supplement: Related texts

# Sub article: Impact of temperatures and plant species on the biological features of the castor bean whitefly Trialeurodes ricini

**DOI:** 10.1673/031.008.0403

**Published:** 2008-01

**Authors:** N.F. Abdel-Baky

**Affiliations:** Economic Entomology Department, Faculty of Agriculture, Mansoura University, Mansoura, Egypt.

Laboratory studies were carried out to determine the influence of temperature (abiotic) and host plant species (biotic) on the biological characters of the castor bean whitefly, *Trialeurodes ricini* Misra. Four temperatures (15, 20, 25 and 30°C), as well as the three plant species, castor bean (*Ricinus communis* L), papaya (*Carica papaya* L.), and sweet potato (*Ipomoea batatas* L.) were tested. Temperature affected insect development, oviposition, and generation time of *T. ricini* when reared on castor bean plants. At 30 °C, egg incubation period, development of nymphal instars, adult longevity and generation time were shortest, followed by 25 °C, while these characters were longer when the insects were reared at 15 °C. The hatching percentage and female fecundity were greater at both 30 and 25 °C and lower at 15 °C. The temperature threshold (t0) and thermal accumulative effect (degree-days) were also calculated. The laboratory studies were confirmed by field applications examining the relationship between seasonal temperature and insect populations. The study demonstrates that *T. ricini* can, in otherwise unlimited conditions, persist and increase in number within the range 20–30 °C. Therefore, the pest is well adapted to high temperatures and may extend its distribution if the mean world temperatures increase because of global warming. Regarding the plant host species, the castor bean was the preferred host followed by papaya, while the sweet potato was not preferred. Host plant species had a significant effect on egg hatching, nymphal survival, female fecundity and the duration of the life cycle of *T. ricini*.

# Sub article: Squash Vein Yellowing Virus, A Novel Ipomovirus, Isolated from Squash and Watermelon in Florida

**DOI:** 10.1673/031.008.0404

**Published:** 2008-01

**Authors:** Scott Adkins, Susan E. Webb, Diann Achor, Chandrasekar S. Kousik, Pamela D. Roberts, Carlye A. Baker

**Affiliations:** ^1^ USDA-ARS, Fort Pierce, FL, USA.; ^2^ University of Florida, Gainesville, FL, USA; ^3^ University of Florida, Lake Alfred, FL, USA; ^4^ USDA-ARS, Charleston, SC, USA; ^5^ University of Florida, Immokalee, FL, USA; ^6^ FDACS-DPI, Gainesville, FL, USA

A novel whitefly-transmitted member of the family *Potyviridae* was isolated from a squash plant (*Cucurbita pepo*) with vein yellowing symptoms in Florida. The virus, for which the name *Squash vein yellowing virus* (SqVYV) is proposed, has flexuous rod-shaped particles of ∼840 nm in length. SqVYV was transmitted by whiteflies (*Bemisia tabaci*, biotype B) but was not transmitted by aphids (*Myzus persicae*). The experimental host range was limited to species in the Cucurbitaceae, with the most dramatic symptoms observed in squash and watermelon, but excluded all tested species in the Amaranthaceae, Apocynaceae, Asteraceae, Chenopodiaceae, Fabaceae, Malvaceae and Solanaceae. Initial greenhouse and field screening of watermelon germplasm with SqVYV has identified potential sources of resistance and experiments are in progress to confirm these preliminary observations. Infection of squash and watermelon by SqVYV induced inclusion bodies visible by electron and light microscopy that were characteristic of members of the family *Potyviridae*. Comparison of the SqVYV coat protein gene and protein sequences with those of recognized members of the family *Potyviridae* indicate that it is a novel member of the genus *Ipomovirus*. A limited survey revealed that SqVYV was present over the five most recent growing seasons in watermelons suffering from a mature vine decline and fruit rot observed in southwest and west central Florida. Inoculation of greenhouse-and field-grown watermelon plants with SqVYV at different stages of growth was sufficient to induce these symptoms, suggesting that it is the likely cause of this disease.

# Sub article: Making a Friend from a Foe: Expressing a GroEL Gene from the Whitefly Bamisia tabaci in the Phloem of Tomato Plants Confers Resistance to Tomato Yellow Leaf Curl Virus

**DOI:** 10.1673/031.008.0405

**Published:** 2008-01

**Authors:** F. Akad, A. Eybishtz, D. Edelbaum, O. Dar-Issa, N. Iraki, H. Czosnek

**Affiliations:** ^1^ The Otto Warburg Minerva Center for Agricultural Biotechnology & The Robert H. Smith Institute for Plant Science and Genetics in Agriculture, The Hebrew University of Jerusalem, Rehovot, Israel.; ^2^ UNESCO Biotechnology Center, Bethlehem University, Bethlehem, West Bank, Palestinian Authority

Breeding for virus resistance in transgenic crop plants is based on a variety of strategies such as expressing pathogen-derived genes or on RNA-mediated gene silencing. The strategy used in the present research is based on a totally new concept. It takes advantage of the fact that some, and perhaps all, plant viruses transmitted by their insect vectors in a circulative manner interact in the insect haemolymph with GroEL homologues produced by the vector endosymbiotic bacteria. It has been suggested that GroEL-virus interaction could be a mechanism shared by plant circulative viruses to avoid destruction in the haemolymph. In this study, we have exploited this phenomenon to generate transgenic tomato plants expressing the whitefly GroEL in their phloem. We expected that once inoculated by their vector, phloem-limited circulative viruses will be trapped by GroEL in the plant phloem, thereby inhibiting invasion of phloem-associated cells and long distance movement, rendering the plants resistant to the virus. A gene encoding a GroEL homologue from the whitefly *B. tabaci* was cloned in an *Agrobacterium* binary vector under the control of an *Arabidopsis* phloem-specific promoter, which was used to transform two tomato genotypes. GroEL was expressed in the two genotypes, during two consecutive generations. All the twenty four To GroEL-transgenic tomato plants obtained (but one and its progeny) exhibited good levels of resistance to whitefly-mediated inoculation of TYLCV, exhibiting mild or no disease symptoms. The transgenic progeny of the resistant plants expressed GroEL and were as resistant as their parents. Resistant plants of the To and T1 generations contained less viral DNA than susceptible non-transgenic plants. In T1 resistant plants, *in vitro* assays indicated that viral particles were complexed with GroEL in the plant sap. The mode of interaction between GroEL and TYLCV, the effect of this complex on the cell-to-cell and long-distance spread of the virus, and the mechanism by which resistance is established, remain to be investigated.

# Sub article: Isolation of Bacillus thuringiensis Strain from Kuwait's Soil Effective Against Whitefly Nymphs

**DOI:** 10.1673/031.008.0406

**Published:** 2008-01

**Authors:** Y. Al-Shayji, N. Shaheen

**Affiliations:** Biotechnology Department, Kuwait Institute for Scientific Research, Shuwaikh, Kuwait.

The whitefly *Bemisia tabaci* Gennadius is considered one of the most important insect pests attacking vegetable crops in the greenhouse and outdoors in Kuwait. Current tactics to manage this pest are largely based on insecticide applications. However, recent concern of Gulf countries about the side effects of chemical pesticides on the environment has resulted in the restriction in the use of these products. Biological controls, using *Bacillus thuringiensis* (*Bt*) have proven to be a valuable alternative to conventional insecticides. In this respect, we have successfully isolated a strain of *Bacillus thuringiensis* capable of producing toxins when grown at two different temperatures 30°C and 50°C active against this serious pest that affects crop production in Kuwait. Laboratory studies were conducted to determine the insecticidal effects of this isolated strain grown at the two temperatures on the 1^st^ instar nymphs of *B. tabaci*. The mortality percentages were 68.2% and 60.0% for the two temperatures respectively, at a concentration of 500µg/ml. Further tests will be followed to study the effect of the toxin on the other stages of *Bemisia* and to clarify the mode of action of this toxin against this sucking insect. We also found that this strain exhibits insecticidal effects on the aphid *Aphis gossypii* and the leaf miner, *Liriomyza bryoniae*. When *Bt* toxin was grown at 30°C and 50°C, the mortality percentages of aphid nymphs were 93.3 % and 90.0 % respectively and of leaf miner pupae were 88.9% and 77.8% respectively.

# Sub article: Activity of Some Biorational and Conventional Insecticides against Bemisia tabaci their Compatibility with Whitefly Parasitoids

**DOI:** 10.1673/031.008.0407

**Published:** 2008-01

**Authors:** Judit Arnó, J. Roig, R. Gabarra

**Affiliations:** Departament de Protecció Vegetal. IRTA, Cabrils (Barcelona), Spain.

We have evaluated the efficacy of several compounds with insecticidal activity against *Bemisia tabaci* nymphs and adults and their compatibility with whitefly parasitoids. The trials were conducted in 2004 and 2005 in exclusion cages inside an experimental greenhouse of poinsettia. The plants were colonized with natural populations of *B. tabaci* (biotype Q) and the parasitoids *Eretmocerus mundus, Encarsia pergandiella* and *En. formosa*. Seven different commercial products were tested, including two potassium soaps (Tec Bom™ and Soapline 60™), one mineral oil (Sunspray Ultrafine™), neem (Iber-Neem™), algae extracts (Agri Sea-Green™) and two conventional insecticides: thiamethoxam (Actara™) and thiacloprid (Calypso™). Water was used as a control. To test the efficacy of the products to control *B. tabaci* nymphs and their effect on parasitoid emergence, all the compounds, except Actara™, were sprayed on poinsettia plants until run-off. Actara™ was applied by irrigation at two doses, the maximum labeled rate and a 2.5 times higher rate. To evaluate nymphal mortality, 2 days after the treatment, plants were taken to the laboratory where samples (1.5 cm diameter disks) were cut and stored in a climatic chamber at 25°C. Seven days after the treatment, the number of dead and live *B. tabaci* nymphs was evaluated. To estimate the impact on *B. tabaci* parasitoids, 2 days after the treatment 100 parasitized pupae were isolated and held for at least two weeks. Number of emerged adult parasitoids was recorded. In another experiment, the efficacy of the products to control *B. tabaci* adults was tested. The same compounds mentioned above were applied on poinsettia plants naturally infested with whitefly adults and their survivorship was evaluated 72 hours after the treatment. Efficacy to control *B. tabaci* nymphs was very limited for all the tested products. The mineral oil Sunspray Ultrafine was the product that killed most nymphs (58%). A second group of compounds, including Tec-Bom, Agri Sea-Green and Calypso, produced mortalities between 30 and 49%, while, Actara, at both doses, and Iber-neem killed less than 20% of whitefly nymphs. Regarding toxicity on *B. tabaci* adults, the two conventional insecticides Actara (at labeled dose) and Calypso produced the highest mortalities (70 and 88%, respectively). Less effective were the soaps (Tec-Bom and Soapline 60) and the mineral oil Sunspray Ultrafine that produced mortalities ranging from 35 to 40%. The least effective among the tested compounds, were Agri Sea-Green and Iber-neem (causing mortalities around 20%). None of the insecticides evaluated caused mortality to *Er. mundus* significantly different than the control. On the other hand, emergence of *En. formosa* adults from pupae treated with Soapline 60 was significantly greater than emergence from pupae treated with Tec-Bom, Sunspray Ultrafine and Agri Sea-Green. *En. pergandiella* proved very sensitive to all the tested products and significantly fewer parasitoids emerged from pupae treated with all the formulations when compared with the control. To control *B. tabaci* nymphs, our results demonstrate than some of the called “biorational” insecticides (e.g. potassium soaps or mineral oil) are as effective as conventional insecticides, or even better. In contrast, to control *B. tabaci* adults, the most effective products were the two conventional insecticides tested (Actara and Calypso). Therefore, since *B. tabaci* is difficult to control, it could be useful to integrate biological control with safe insecticides to natural enemies. To do so, our results indicate that the impact on biological control of some of the termed “biorational” insecticides should not be neglected and, the integration of both control methods will depend on the parasitoid species used.

# Sub article: Bemisia tabaci Biotype Q and its Natural Enemies in Vegetable and Ornamental Crops

**DOI:** 10.1673/031.008.0408

**Published:** 2008-01

**Authors:** Judit Arnó, J. Roig, R. Gabarra

**Affiliations:** Departament de Protecció Vegetal. IRTA-Cabrils. Cabrils (Barcelona), Spain.

Vegetables in some Mediterranean areas are grown mainly on small family operated farms. Vegetable crops are grown year- round in open fields and greenhouses built with metal frames, covered with plastic films, and mostly unheated. Vegetable production coexists with ornamental crops either in the open field or in heated greenhouses. In this situation, whiteflies reproduce all year with continuous carry-over among different crops and environments. For many years, *Trialeurodes vaporariorum* has been one of the main pests. However, from the early 90's, populations of *Bemisia tabaci* have been increasing in vegetable and ornamental crops. In the area of study, the northeast of Spain, tomato, cucumber and poinsettia crops are often heavily infested by *B. tabaci*. This pest usually coexists with *T. vaporariorum* and their relative abundance changes with crop and season. Tomato is grown in three different crop cycles from February to December in greenhouse and outdoors. Cucumber is grown mainly in greenhouses in two crop cycles from March to November. Finally, poinsettia is grown for the Christmas market in heated greenhouses from July to December. In tomato, biological control of whiteflies, by conservation and augmentation of native populations of the mirid bug *Macrolophus caliginosus*, is used by many growers. In cucumber, no specific biological control program is applied and, although the crop is sprayed more often that tomato, natural enemies can be preserved to a certain extent. In poinsettia, releases of the parasitoid *Eretmocerus mundus* to control *B. tabaci* populations together with the use of soft insecticides began in 2003 in few farms as an alternative to chemical control of the pest. In this scenario, we wanted to determine the importance of three *B. tabaci* sensitive crops (tomato, cucumber and poinsettia) on the survival of this whitefly, and to evaluate the natural enemies associated with *B. tabaci* and *T. vaporariorum*. For this purpose, tomato crops (IPM and non IPM) were surveyed: 9 in spring, and 7 in autumn 2003, and 11 in spring and 17 in autumn 2004. We also surveyed 7 cucumber greenhouses during September 2004, 7 poinsettia greenhouses during 2003 and 5 more during 2004. In these poinsettia greenhouses, growers released *Er. mundus* combined with the use of soft insecticides. Thirty tomato leaflets, 30 cucumber leaf-disks (9.6 cm^2^), and 30 poinsettia leaves were sampled to evaluate *B. tabaci* infestation, parasitization and predation. Parasitized pupae were isolated until the emergence of the parasitoid to determine its species. In tomato, *Trialeurodes vaporarioum* was the predominant species in the spring crops whereas *B. tabaci* was the most abundant in autumn crops. In cucumber and poinsettia, *B. tabaci* was almost the only whitefly present. In autumn 2004, although the infestation was very different among greenhouses, average density of *B. tabaci* on cucumber (3.43 nymphs/ cm^2^) was significantly greater than on tomato (0.13 nymphs/ cm2) or poinsettia (0.08 nymphs/ cm2) (F= 43.7; df 2,28; P<0.000). These data showed that cucumber was the most important host where *B. tabaci* built up its population during autumn. Nevertheless, poinsettia allowed *B. tabaci* survivorship in cooler seasons because it was grown in heated greenhouses and could maintain whitefly populations until December. Parasitoids caused the greatest mortality of *B. tabaci* pupae in cucumber (average 18%), but their incidence in tomato was much lower (average 10%). Note that *B. tabaci* parasitoids had not been released neither in cucumber nor in tomato. On the other hand, in poinsettia, *Er. mundus* was released in most of the greenhouses, and parasitism was substantial (average 56%). *Encarsia pergandiella* and *Er. mundus* were found parasitizing *B. tabaci* pupae (Table 1) with *Er. mundus* comprising 49 and 58 % of all parasitoids emerging from tomato and cucumber, respectively. Yet, in tomato and cucumber, both species were seldom found together in the same greenhouse (just in 2 out of 21 samples). In contrast, during 2004, both *En. pergandiella* and *Er. mundus* were found together in all the poinsettia greenhouses. Probably, the inoculation of *Er. mundus* in those greenhouses forced the coexistence of both parasitoids. Predator species differed in all 3 crops (Table 1). Predators were particularly important in tomato where *M. caliginosus* was the most abundant. In early tomato crops, where the *B. tabaci* population was very low, average predation reached 20 % of *T. vaporariorum* and 9% of *B. tabaci* nymphs. At the end of the season, percentages increased up to 34 and 46 % of *T. vaporariorum* of *B. tabaci* nymphs, respectively. In cucumber, average predation of nymphs was <1%. This mortality factor was not evaluated in poinsettia due to the low numbers of whitefly predators detected in the crop. In summary, the use of biological control in early tomato crops, where *T. vaporariorum* was the main whitefly species, conserved and enhanced *M. caliginosus* and *En. pergandiella* populations. Later on, these natural enemies caused a great deal of mortality in *B. tabaci* populations in the autumn crops. To reduce overall *B. tabaci* populations in the area, biological control of *B. tabaci* should focus on cucumber, since this crop hosts the highest *B. tabaci* populations. The high natural parasitism rates that we found in some cucumber greenhouses suggest that parasitoid releases could avoid the build up of the pest in this crop. In poinsettia, releases of *Er. mundus* were effective in some greenhouses and reduced dramatically *B. tabaci* populations. However, it would be necessary to maintain the heating until the end of the crop to be able to sustain high levels of parasitism.

# Sub article: Mortality factors affecting populations of Bemisia tabaci on cassava in Uganda

**DOI:** 10.1673/031.008.0409

**Published:** 2008-01

**Authors:** Peter Asiimwe, Justin Ecaat, Michael Otim, Dan Gerling, Moshe Guershon, Samuel Kyamanywa, James P. Legg

**Affiliations:** ^1^ International Institute of Tropical Agriculture-Uganda, Kampala, Uganda.; ^2^ Makerere University, Kampala, Uganda; ^3^ Tel Aviv University, Ramat Aviv, Israel; ^4^ International Institute of Tropical Agriculture-Tanzania, Dar es Salaam, Tanzania; ^5^ Natural Resources Institute, University of Greenwich, Chatham Maritime, Kent, UK

A cohort-based approach was used to determine the sources and rates of mortality of *Bemisia tabaci* on field-grown cassava in Uganda. Daily direct observations were used to construct partial life tables for twelve generations of egg and nymph populations which were studied over a one year period. Mortality was categorized as due to dislodgement, predation, parasitism (for nymphs only), unknown death and inviability (for eggs only). The highest mean rate of marginal mortality across all stages was attributed to parasitism, with dislodgement and predation following respectively. Across all factors, the highest mean rate of marginal mortality was observed in the fourth instar followed by the eggs, first, second and third instars respectively. Key factor analysis revealed that dislodgement was the major mortality factor contributing to generational mortality in eggs while for nymphs, parasitism in the fourth instar was the main driving force behind the observed generational mortality. Highest irreplaceable mortality in both the egg and nymph stages was attributed to dislodgement followed by parasitism, predation and least was due to unknown death. Across stages, the highest irreplaceable mortality rates were observed in the eggs and the fourth instar nymphs. The other stages had relatively low rates of irreplaceable mortality. Rain protection experiments revealed no significant differences in marginal mortality rates when compared to the open field situation.

# Sub article: Nutritional factors influencing whitefly development and flight behavior: The search for a suitable bioassay to test hypotheses

**DOI:** 10.1673/031.008.0410

**Published:** 2008-01

**Authors:** Jackie. L. Blackmer, Linda L. Lee

**Affiliations:** USDA-ARS, Arid-Land Agricultural Research Center, Maricopa, Arizona, USA

Obtaining adequate nitrogen (N) is considered to be one of the major evolutionary hurdles phytophagous insects had to overcome before they could exploit plants. Phloem feeders, like whiteflies, encounter some of the lowest levels of N, but they have excelled in this niche owing to physiological, morphological and behavioral modifications that suite them well. For most whitefly species, these traits are merely fascinating curiosities; however, for several polyphagous whitefly species, understanding the mechanisms that allow them to be so successful may provide us with insights that could be exploited in their control. One behavioral strategy that these insects use in dealing with inadequate or unbalanced nutrient levels is emigration. However, we know very little about the underlying control mechanisms that lead to the initiation of emigration. Changes in the levels of various amino acids or in the ratio of carbohydrates to amino acids almost certainly play a role in assessing the suitability of a given host for oviposition and feeding, and once host quality declines, may supply the cues that signal that it is time to emigrate. Previously, we showed that various life-history traits of *Bemisia tabaci* (Gennadius) were correlated with the relative concentrations of essential amino acids that fluctuated during the growing season in *Cucumis melo*, and that increases in serine and glutamine/glutamic acid during senescence were correlated with increases in flight activity measured in a vertical flight chamber. These findings were interesting, but without a proper bioassay where N concentrations could be manipulated, hypotheses generated from this study could not be tested. This led us to investigate an artificial rearing system for whiteflies where amino acid and carbohydrates could be manipulated. While it was possible to raise whiteflies to the red-eye stage, very few individuals emerged to the adult stage and those that did were too weak to fly in our flight chamber. Currently, we are using TurboGarden™ hydroponic units with rockwool media to grow cotton plants. A top feed dripper manifold supplies varying concentrations of macro and micronutrients. Oviposition, % hatch, developmental time, emergence rates, adult weights and flight behavior of adults were measured. Because whiteflies have the ability to increase their feeding rates to compensate for lower N levels, we also indirectly measured feeding rates on each of the nutrient regimes by counting and measuring honeydew deposited on aluminum foil placed below leaves containing nymphs. It is thought that if nutrients are limiting then the honeydew will lack key amino acids, so after honeydew drops were counted they were collected and analyzed for amino acids.

# Sub article: Genetic distance analysis among a large set of Bemisia tabaci samples

**DOI:** 10.1673/031.008.0411

**Published:** 2008-01

**Authors:** Petra Bleeker, Suzan Gabriëls, Kai Ament, Paul Diergaarde, Isa Lesna, Michel Haring, Michiel de Both, Robert Schuurink

**Affiliations:** ^1^ Keygene NV, Wageningen, The Netherlands.; ^2^ University of Amsterdam, Swammerdam Institute of Life Sciences, Dept. Plant Physiology, Amsterdam, The Netherlands

Whitefly (*Bemisia tabaci*) infestation and the consequent transfer of virus particles is responsible for severe losses in cultivation of many crop species, all over the world. Conventional control of whitefly infestations has proven to be difficult and therefore we aim towards the development of whitefly resistance in crop species. By studying the plant-insect interaction we aim to establish the preference behavior of *B. tabaci* on certain crop species. It is known that within the species there is considerable genetic variation between populations. Within the scope of this project we set out to characterize a large set of whitefly populations from all over the world taken from different crops. *B. tabaci* was collected from several relevant locations (research stations of seed companies in the production areas of Japan, Indonesia, Thailand, India, Brazil, Mexico, Israel, Turkey and Spain) and from different crop species (tomato, melon, pepper, squash, eggplant, oilseed rape). The amplified fragment length polymorphism (AFLP) technique was used to investigate the extent and nature of intraspecific variability of 35 different samples and to determine whether these populations comprise of multiple biotypes. Also, we established whether *B. tabaci* samples collected in the production areas differ from our lab culture, which originates from the Research station for Floriculture in Aalsmeer (The Netherlands).

# Sub article: Resistance of Common Bean Genotypes to Bemisia tabaci (Genn. 1889) Biotype B (Hemiptera: Aleyrodidae) in Two Growing Seasons

**DOI:** 10.1673/031.008.0412

**Published:** 2008-01

**Authors:** Arlindo L. Boiça, F. Gonçalves de Jesus, R. M. Pitta, M. C. Salvador, C. P. Stein

**Affiliations:** ^1^ Departamento de Fitossanidade — Faculdade de Ciências Agrárias e Veterinárias — Campus de Jaboticabal/SP, Brasil.; ^2^ Setor de Entomologia, Institute Agronômico-IAC, Campinas, SP, BR

Brazil is a major producer and consumer of common bean (*Phaseolus vulgaris* L.), which is the most important source of vegetable protein in the country. Among all pests attacking common bean, the whitefly *Bemisia tabaci* biotype B has become a key pest, causing substantial losses in many producing regions of the country. The whitefly transmits the bean golden mosaic virus (BGMV), which limits productivity and sometimes causes yield losses of 100%. Whitefly has been controlled with insecticides and cultural practices. Because of the environmental impacts of insecticides, alternative control methods have been sought. Another important approach is the use of resistant genotypes in an integrated pest management (IPM) program, which has shown great potential in controlling *B. tabaci* biotype B. In this work, we study the resistance of level of common bean genotypes to whiteflies in the rainy and dry seasons. The experiment was conducted from December 2005 to February 2006 (rainy season), and from May to June 2006 (dry season) at the experimental fields of the Department of Plant Protection (Departamento de Fitossanidade), FCAV/UNESP — Jaboticabal, SP — Brazil. Seed was sown in rows spaced 0.5 m at a density of 12 plants per meter. Fertilizer was applied at planting at a rate of 430 kg ha-1 of the formula 04-14-08 NPK, and 20 days after emergence (DAE) with 180 kg ha-1 of ammonium sulfate. The experiment was arranged in randomized blocks with three replications and nineteen treatments corresponding to the genotypes: IAC-Carioca Tybatã, IAC-Una, FT-Nobre, Pérola, Gen 96A98-15-3-32-1, Gen 96A45-3-51-52-1, Gen 96A98-13-1-52-1, Gen 96A98-5-1-1-55, Gen 96A3-P1-1-1, LP 98-122, LP 02-130, LP 01-38, LP 9979, BRS-Pontal, BRS-Requinte, BRS-Triunfo, BRS-Grafite, CV-48 and Z-28. Plots consisted of four rows of 4 m, covering 4.0 m^2^ of total area and 2.0 m^2^ of useful area. The total experiment area was 840 m^2^. Whitefly infestation was monitored on a weekly basis by counting *B. tabaci* biotype B eggs and nymphs on 10 leaflets per plot, until 60 day after emergence. The occurrence of BGMV was monitored 30 and 50 DAE, but no symptoms were observed. Data were analyzed by Fisher's test and submitted to Duncan multiple range test at 5 % of probability. In the rainy season, a significantly higher egg count was observed only in FT-Nobre, at 53 DAE. The highest nymph counts were found on different dates in each genotype. From 25 to 39 DAE the nymph count varied, although highest counts were observed mostly at 32nd DAE. The highest egg count in the dry season experiment was seen on genotypes Gen 96A98-13-1-52-1 (39 DAE) and Gen 96A98-5-1-1-55 (53 DAE). The nymph population per leaflet was higher on most genotypes from 25 to 39 DAE, and then it tended to decrease. However, on genotype BRS-Pontal the highest population was observed 53 DAE, while in IAC Tybatã population peaks were seen 25 and 60 DAE. In Gen 96A98-5-1-1-55 only one peak was observed 60 DAE. Our results showed that in the rainy season the most critical attacks took place until 39 DAE, with a population peak at 32^nd^ DAE. In the dry season, most genotypes showed infestation increases until 39 DAE, when the highest population was observed. The genotypes studied showed varying results in terms of *B. tabaci* biotype B oviposition, depending on the plant age and growing season (rainy or dry).

# Sub article: Control Strategies for Bemisia tabaci (Genn.) Biotype B and Other Insect Vectors in Tomato Lycopersicum esculentum (Mill.)

**DOI:** 10.1673/031.008.0413

**Published:** 2008-01

**Authors:** Arlindo L. Boiça, M. A. A. Macedo, N. R. Chagas Filho, M. R. Angelini, A. L. Boiça Neto

**Affiliations:** ^1^ Departamento de Fitossanidade — Faculdade de Ciências Agrárias e Veterinárias — Campus de Jaboticabal/SP, Brasil.; 2 Departamento de Proteção de Plantas — Faculdade de Ciências Agrárias da Unesp — Campus de Botucatu/SP, Brasil; 3 Pontifícia Universidade Católica — PUC, Campinas/SP, Brasil

Tomato (*Lycopersicum esculentum*, Mill.) is considered to be a high risk crop due to infestation of many pests, in fields intended for the production of fruits to be consumed *in natura* or processed. Because insect pests attack tomato plants during their entire cycle, it is very challenging to grow tomatoes under field conditions. To control early pests (thrips, aphids and whiteflies) many insecticide sprayings are required, sometimes every 3 days throughout the plant cycle, often combining two or more products. However, this procedure has resulted in the selection of resistant insect populations and environmental problems. In this study, we compare insect vector control techniques in tomato cultivars. Two experiments were carried out. The first was conducted in Cafelândia/SP with the cultivars AP 529 and H 108, to compare the following control techniques: 1) conventional, with application of carbofuran 50 G (soil) and sprayings with acephate 750 BR, metamidophos 600, thiacloroprid, imidacloprid 700 GRDA, chlorfenapyr, fenitrothion 500 CE, carbaryl 480 SC and abamectina 18 CE; 2) integrated Pest Management (IPM) with application of carbofuran 50 G, and adopting the action threshold for vector pests to spray imidacloprid 700GRDA, triflunuron 250 PM, lufenuron CE and abamectina 18 CE; 3) IPM and carbofuran 50 G, adopting the action threshold to spray 0.5% Neem oil extract (1.2% azadirachtin); 4) IPM adopting the action threshold to spray 0.5% Neem; 5) untreated control. The second experiment was conducted in Guarantã/SP with the cultivars AP 529 and Malinta, comparing the techniques: 1) conventional, with application of carbofuran 50 G and sprayings as described before; 2) IPM adopting the action threshold to spray 0.5% Neem; 3) sorghum barrier with no spraying; 4) maize barrier with no spraying; 5) IPM adopting the action threshold to spray 0.5% Neem plus sorghum barrier; 6) IPM adopting the action threshold to spray 0.5% Neem plus maize barrier; 7) control. Control treatments were conducted with the same cultivars without insecticide or Neem applications. The ‘conventional technique’ consisted of the same approach used by farmers, in which insecticides are sprayed according to a pre-set schedule, every three to six days. The mean number of adults of *Bemisia tabaci* (Genn.) biotype B, *Frankliniella schultzei* (Trybom) and *Myzus persicae* (Sulz.), the total vector count per plant, the rate of plants with virus symptoms and tomato yield (t.ha^-1^) were determined. Data were submitted to an ANOVA and F test through the software ESTAT and means were compared by Tukey test at 5% of probability. No difference was observed for cultivars in the first experiment in terms of pest infestation. However, whiteflies were observed in higher levels than aphids or thrips in both cultivars. The conventional control technique resulted in the lowest number of adults per plant (1.46, 0.79 and 0.86 whiteflies, thrips and aphids, respectively), followed by IPM with carbofuran 50 G plus insecticide sprayings, and IPM with Neem oil extract and carbofuran 50 G. The conventional control also resulted in the highest productivity (52.24 t.ha^-1^), followed by IPM with carbofuran 50 G (48.45 t.ha^-1^) and IPM with Neem and carbofuran 50 G (45.96 t.ha^-1^). Compared to the untreated control, these techniques increased tomato productivity in 79.52, 66.50, 57.94, and 56.67%, respectively. The second experiment showed no difference between cultivars in total vectors (whiteflies, aphids and thrips), however the whitefly population was the highest. Conventional control allowed the highest reduction in white fly population followed by IPM with Neem oil extract and maize barrier, with 1.33 and 2.31 adults per plant, respectively. The same was observed for total vectors, with means of 2.46 and 3.87 per plant for conventional control and IPM with Neem oil extract and maize barrier. The productivity was inversely proportional to the mean number of insects. The conventional technique showed the lowest infestation rate and the highest productivity (62.72 t ha^-1^). The sorghum barrier was not effective in controlling insect vectors, being statistically similar to the untreated control. Productivity increases of 71.10, 41.13, 4.70, 40.45 and 48.18% were reached with IPM with Neem, sorghum barrier without sprayings, maize barrier without sprayings, IPM with Neem and sorghum barrier, and IPM with Neem and maize barrier, respectively. The whitefly infestation is higher in comparison to the other virus vectors, and the conventional control is the most efficient, followed by IPM with Neem oil extract and carbofuran, and IPM with Neem oil extract and maize barrier, which are all promising to control these pests.

# Sub article: Out of Africa: Diversity and host plant utilization in sub-saharan Bemisia tabaci

**DOI:** 10.1673/031.008.0414

**Published:** 2008-01

**Authors:** L. M. Boykin, C. L. McKenzie, R. C. Resell, R. A. Bagnall, R. G. Shatters, D. R. Frohlich

**Affiliations:** ^1^ USDA US Horticultural Research Laboratory, Ft. Pierce, FL, USA.; ^2^ Biology Department, University of St. Thomas, Houston, TX, USA

The diversity of *Bemisia tabaci* in Sub-Saharan Africa is greater than in any other geographic location and suggests Sub-Saharan Africais the likely evolutionary origin of the species. Sampling in Africa is now quite extensive and includes a number of studies that have analyzed host preference within a phylogenetic framework. None of these studies have used consistent nomenclature for the major groups or included all available sequences. We obtained all *B. tabaci* mitochondrial COI sequences in Genebank from Africa and analyzed them in a Bayesian framework. Herein, we describe a phylogenetic reconstruction and examine those relationships within the parameter of host plant utilization. Discussion of consistent nomenclature for these major clades in Sub-Saharan Africa will also be addressed.

# Sub article: Bayesian phylogenetic analysis of mitochondrial COI DNA sequence from global samples of Bemisia tabaci (Hemiptera: Aleyrodidae)

**DOI:** 10.1673/031.008.0415

**Published:** 2008-01

**Authors:** L. M. Boykin, R. G. Shatters, R. C. Resell, C. L. McKenzie, R. A. Bagnall, Paul De Barro, D.R. Frohlich

**Affiliations:** ^1^ USDA US Horticultural Research Laboratory, Ft. Pierce, FL, USA.; ^2^ Biology Department, University of St. Thomas, Houston, TX, USA; ^3^ CSIRO Entomology, Indooroopilly, Australia

*Bemisia tabaci* (Gen.)(Hemiptera: Aleyrodidae) is one of the most devastating agricultural pests worldwide and affects the yield of a broad range of agricultural, fiber, vegetable and ornamental crops. Global phylogenetic relationships of the major races of *B. tabaci* remain unresolved thus a Bayesian phylogenetic technique was utilized to elucidate relationships. All COI DNA sequence data available in Genbank for *B. tabaci* world-wide (367 specimens) were obtained and the first well resolved phylogeny for the *B. tabaci* species complex was produced. Nine major well resolved (0.70 posterior probability or above) races were recovered and are: *B. tabaci* (Mediterranean/Asia/Africa-B Biotype), *B. tabaci* (Mediterranean-Q Biotype), *B. tabaci* (MS Biotype), *B. tabaci* (Q-related Africa), *B. tabaci* (Asia I), *B. tabaci* (Asia II), *B. tabaci* (New World-A Biotype), *B. tabaci* (sub-Saharan Africa), and *B. tabaci* (Uganda sweetpotato). *B. tabaci* is of African origin as indicated by position of the sub-Saharan African clade at the base of the global phylogeny. Re-analyses of ITS, COI, and a combined data set from a previous study resulted in seven major well resolved races with high posterior probabilities, also showing the utility of the Bayesian method. Relationships of the nine major *B. tabaci* races are discussed herein.

# Sub article: Cytoplasmic incompatibility phenotypes from reciprocal crosses of three biotypes of the Bemisia tabaci complex are associated with Cardinium (Bacteroidetes) and Wolbachia, and apparent partial rescue of CI Yyelding sex bias

**DOI:** 10.1673/031.008.0416

**Published:** 2008-01

**Authors:** J. K. Brown, Rafael De J Caballero

**Affiliations:** ^1^ Department of Plant Sciences and; ^2^ Department of Entomology, The University of Arizona, Tucson, AZ, USA.

We investigated the effects of two divergent, prospective cytoplasmic incompatibility (CI)-causing bacteria on gene flow among biotypes of the taxonomically confounded *B. tabaci* species group. We report the detection and identification by PCR, cloning, sequencing, and phylogenetic analysis of several endosymbiotic bacteria associated with three biotypes of *B. tabaci*: the polyphagous New World (NW) Arizona A (AzA), the polyphagous Old World (OW) Arizona B (AzB), and the monophagous NW Jatropha (Jat). Based on PCR amplification of the 16S rRNA, a *Cardinium* spp. (phylum *Bacteriodetes*) was associated with the AzA and three other A biotype isolates (RivA, CulA, SalA lab colonies), whereas a *Wolbachia* spp. (*Rickettsia*; Proteobacteria) was consistently associated with the Jat biotype. *A Chlamydia* spp. and *Rickettsia bellii* also were detected in certain but not all *B. tabaci* isolates, and are presumably secondary endosymbionts more likely associated with general fitness traits. To date neither has not been implicated in cytoplasmic incompatibility in the hemiptera, however such involvement cannot be ruled out. In a previous study, we reported partial and complete reproductive compatibility between three biotypes (AzA, B, Jat), respectively. In this follow-up study we have confirmed the association of two putative CI-bacteria, *Wolbachia* and *Cardinium*, with the Jat and A biotypes of *B. tabaci*, corroborating the CI-like phenotype observed in crosses between the AzA, Az B, and Jat biotypes, expressed as unidirectional incompatibility (AzA x B; Jat x B), and surprisingly, bidirectional compatibility (Az A x Jat). Identical results were obtained for A x B crosses when Riverside A was substituted for the AzA. Bidirectional female offspring produced from Jat x AzA crosses may be indicative of a rescue phenomenon by these divergent CI-causing bacteria. Further, results of crosses employing *Wolbachia*-Jat♀x *Cardinium*-AzA♂, indicated that fewer female offspring were produced in that cross, compared to AzA-*Cardinium*♀x Jat-*Wolbachia*♂. This observation suggested that *Cardinium* might more ably abate *Wolbachia* induced female offspring mortality than *Wolbachia* is able to counter *Cardinium*-induced CI. These results when taken together provide evidence that two phylogenetically divergent CI-bacteria might contribute operatively to *B. tabaci* reproductive incompatibility, expressed differentially, and not universally as barriers to gene flow. And, in at least one case this interaction also is seen as sex bias. Herein we report a novel, rescue-like phenomenon between two phylogenetically divergent bacteria, which could possibly have evolved similar CI-inducing mechanisms, making possible the rescue of CI effects induced by the other. Due to the inability to rid *B. tabaci* of these suspect CI-bacteria using various treatments effective for other insects, an introgression experiment is in progress to explore CI-causality over CI-association.

# Sub article: Positive evidence for interbreeding and differential gene flow between three well characterized biotypes of the Bemisia Tabaci complex (Gennadius) (Hemiptera: Aleyrodidae) excludes geographic and host barriers as isolating factors

**DOI:** 10.1673/031.008.0417

**Published:** 2008-01

**Authors:** Rafael De J Caballero, J. K. Brown

**Affiliations:** ^1^ Department of Entomology and; ^2^ Department of Plant Sciences, The University of Arizona, Tucson, AZ USA.

Reciprocal crosses were carried out with single pairs or groups of males and females for three well-characterized biological types (biotypes) of the whitefly *Bemisia tabaci*. Two polyphagous biotypes, the polyphagous Arizona A biotype (AzA) originating in the Western U.S.A., the polyphagous B biotype (AzB) from the Eastern Hemisphere, and the monophagous Jatropha (Jat) biotype from Puerto Rico, were used in crossing experiments (27°C, 80% humidity, 12:12 hr photoperiod). Reciprocal and homologous crosses were established between single pairs or groups of 20 virgin females and males. All crosses employed cotton as the oviposition host except for Jat female x AzA or AZB male crosses, for which *Jatropha gossypifolia* (Euphorbiaceae) was the host. Whitefly pairs and groups were caged together on the respective oviposition host for 7 and 10 days, respectively. Genetic identification of parents and selected F1 and F2 offspring was carried out using the maternally inherited mitochondria cytochrome oxidase I sequence. All homologous crosses yielded female offspring. Female offspring were produced in all but two reciprocal crosses. These exceptions were AzB♀ x AzA♂, or AzB♀ x Jat♂, for which female offspring were produced in a single direction. Because this distinctive pattern could be attributable to endosymbiont-induced incompatibility, whitefly colonies were screened for bacterial presence using PCR and 16S rRNA primers revealing that the AZA biotype harbored *Cardinium*, and that a *Wolbachia* spp. was associated with Jat. That *Wolbachia* and *Cardinium* are implicated in cytoplasmic incompatibility (CI) and/or other types of reproductive incompatibility makes this finding intriguing, and so their discovery has been the subject of a follow-on study. These results provide the first evidence for gene flow between monophagous and polyphagous New World biotypes (A, Jat, respectively), and between an Old World (B) and two New World (A, Jat) biotypes, indicating that geographic and host barriers do not mandate reproductive isolation. These results challenge the proposed classification of the B biotype as a species unique from all other *B. tabaci*. Based on the biological species concept, reproductively compatible organisms that produce viable offspring would be considered the same species. Several lines of evidence, including the results presented here, suggest that *B. tabaci* constitutes a single group of related variants, of which some are reproductively compatible while others are not. Also, certain variants are recognizable by definitive sets of (adaptive) behaviors, and are referred to as biotypes, a designation that has contributed to taxonomic confusion. Even so, results indicate that gene flow occurs between three of these *B. tabaci* variants, in this instance ‘biotypes’, which have experienced prolonged physical separations imposed by geography and/or host plant. It is not known whether the A, B, Jat, or other biotypes and haplotypes have hybridized in nature and/or if so, whether they are naturalized. Hybridization may more commonly be employed as a means of diversification in this species than previously thought.

# Sub article: Survival of whiteflies during long-distance transportation of agricultural products

**DOI:** 10.1673/031.008.0418

**Published:** 2008-01

**Authors:** Piero Caciagli

**Affiliations:** Istituto di Virologia Vegetale — CNR, Torino, Italy.

Besides being pests of tropical and subtropical crops and of protected crops in temperate regions, some whitefly species are also vectors of many plant viruses. Worries about their possible movements to new areas are therefore justified. Although whitefly species are known to have invaded new areas through natural spread, in general whiteflies are poor fliers and their long-distance movements are likely assisted by humans. The high risk of moving pests from place to place by human activity is one of the few agreed reasons for imposing quarantine restrictions. Hence, it is very important to know, besides other things, the conditions that permit survival of the pest. Among the abiotic factors influencing survival of whiteflies, temperature and relative humidity are important, particularly at extreme values. Temperature has two limits for the survival of whiteflies. High transport temperatures are not compatible with transportation of fresh plant products, but may be compatible with other types of goods that do not require cooling or controlled conditions. At 30 to 40°C, survival of adult *B. tabaci* is very poor after 6 hours, independently of RH. At the lower limit, adult whiteflies are the least resistant stage, but they can survive for long times at temperatures often recommended for the transportation of potted ornamental plants and florist green materials. Other stages, like eggs and nymphs, are much more resistant to cold. Within the ranges of temperature and time that allow survival of the non-adult stages, products can be moved substantial distances. There is also a possibility that plant pathogenic viruses can be moved together with whitefly immature stages. Among the biotic factors that could influence survival, only the osmotic pressure of the host leaves seems to have a direct bearing. Variations in host leaf water content, and its associated soluble sugar concentration, strongly influence egg survival of *T. vaporariorum*. On the other hand, 4th instar nymphs can survive desiccation of the leaves they are attached to and emerge as adults after a few days. Given that the risk of transporting undesirable species is high, the imposition of quarantine regulations is not a decision for scientists. Biologists, though, should provide the rationale behind decisions of this type: not only the basic knowledge of pests (biology, taxonomy), but also risk analysis, in order to establish the priorities for political decisions and for future research, and instruments for making regulations enforceable. Methods are available for identifying the undesirable pests both at the species (official diagnostic protocols, identification guides, ELISA tests, multiplex real-time PCR assays) or biotype level (analysis of esterase patterns, sequencing of whitefly genes, RAPD-PCR, AFLP, PCR-RFLP). The analysis of biotypes/races can be even more detailed by microsatellite markers. Other more general instruments for reducing the risk of exporting pests are available or being developed, with the aim of excluding pests. If exclusion fails, we can still use our knowledge to confine, and hopefully eradicate, the undesired “guest”. Agriculture operators and international travelers should be made aware of the existence of destructive, exotic (plant) pests threatening to enter a country in which they are not known to occur, because it is clear that the success of plant quarantine programs greatly depends upon public cooperation with quarantine legislation.

# Sub article: Controlling the Tobacco Whitefly Bemisia tabaci (Genn.) (Hom.: Aleyrodidae) in Horticultural Crops with the Predatory Mite Amblyseius swirskii (Athias-Henriot)

**DOI:** 10.1673/031.008.0419

**Published:** 2008-01

**Authors:** Javier Calvo, Karel Bolckmans, José E. Belda

**Affiliations:** ^1^ Departamento I+D, Koppert Biological Systems, Águilas (Murcia), Spain.; ^2^ Koppert Biological Systems, AD Berkel en Rodenrijs, The Netherlands

The predatory mite *Amblyseius swirskii* has demonstrated the potential to become a key biological control agent in several horticultural crops in southern Spain. However, actual implementation of *A. swirskii* required clarification of such critical parameters such as optimal release rates, and introduction times, response of pest populations, adaptability to various crops, and compatibility with different natural enemies. The experiments described below were intended to provide this information under simulated field conditions in sweet pepper, cucumber and eggplant. Trials were conducted in a 40 × 10 m greenhouse located in Aguilas (Murcia, Spain). The greenhouse was divided into 36 cages of 4 × 2 m, 12 of which were used for two studies on sweet pepper. The focus of the first experiment was to compare two widely divergent release rates for *A. swirskii* in sweet pepper 25 and 100 mites/m^2^ with an untreated control. Plants were infested by releasing 8 adult whitefly per plant over a 3 week period with the mites released the 3 week. The second experiment compared the efficacy of single species and combinations of several biological control agents: EM (*Eretmocerus mundus* alone), NT (*E. mundus* + *Nesidicoris tenuis*), AS (*E. mundus* + *A. swirskii*) and MIX (*E. mundus* + *N. tenuis* + *A. swirskii*). Plants were infested by releasing 50 adults of *Bemisia tabaci* per plant over 4 weeks in all the treatments. In the first experiment both release rates of *A. swirskii* (25 and 100 mites/m^2^) virtually eliminated whitefly nymphs from the plants. The second experiment demonstrated that the combination of *A. swirskii* and *E. mundus* was most efficacious against whitefly in sweet pepper. The same procedures and treatments (EM, NT, AS, and MIX) were applied to eggplant except that plants were infested by releasing a total of 30 *B. tabaci* adults in each of the 12 cages. The whitefly population was significantly suppressed by treatments (AS and MIX) that included *A. swirskii* was released compared with the remaining treatments. Furthermore, the addition of *N. tenuis* did not contribute significantly to whitefly control (NT and EM). Thus, the best biological control strategy for *B. tabaci* in eggplant was the combination of *A. swirskii* and *E. mundus*. Two trials were conducted in cucumber using 12 and 15 cages respectively, the first focusing on release rates for *A. swirskii* against whitefly and the second, on the relative effectiveness against whitefly and thrips, In the first experiment, infestation was initiated by releasing 35 adults of *B. tabaci* per plant and 4 release rates of *A. swirskii* were compared (0, 25, 50 and 75 mites/m^2^). In the second, 5 treatments were compared: WFC (35 *B. tabaci*/plant), FOC (15 *Frenkliniella occidentalis*/plant), ASWF (35 *B. tabaci*/plant + 75 *A. swirskii*/m^2^), ASFO (15 *F. occidentalis*/plant + 75 *A. swirskii*/m^2^), MIX (35 *B. tabaci*/plant + 15 *F. occidentalis*/plant + 75 *A. swirskii*/m^2^). Significant whitefly control was again observed over the entire range of release rates in the first experiment, although greater suppression was achieved at the higher release rates. The second experiment showed that, *A. swirskii*, was able to provide efficient control of *B. tabaci* in cucumber, and further that *F. occidentalis* could also be controlled, even when both pests appeared simultaneously in the crop. As a general conclusion, *A. swirskii* has demonstrated excellent potential as a biological control agent of *B. tabaci* for horticultural crops in Southern Spain and similar areas. Its implementation enables the use of IPM programs for these crops by controlling *B. tabaci* biologically in many situations, avoiding the necessity of insecticides. Due to its efficacy against whitefly and thrips, it has recently been implemented as a key component in the IPM programs for these crops.

# Sub article: Fertility of Bemisia tabaci and sex ratio determination according to male availability

**DOI:** 10.1673/031.008.0420

**Published:** 2008-01

**Authors:** Steven J. Castle

**Affiliations:** USDA, ARS Arid-Land Agricultural Research Center, Maricopa, Arizona, USA.

The expression of biotic potential in *Bemisia tabaci* populations is recognized worldwide by numerous historical outbreaks and a propensity to routinely attain high density infestations in various crop environments. Fertility rates of females are a crucial aspect of population growth and an important component of the pest potential of herbivorous insects. Based on mean lifetime eggs laid per female, past studies have provided variable estimates of fecundity ranging from <50 to >300. In the present study, per capita reproduction was measured by collecting F1 progeny adults from individual parental pairs, thus providing a measure of fertility. In addition, sex ratios of F1 progeny were determined for both paired and unpaired parental females, thereby allowing the role of male availability to be revealed. Experiments were carried out under natural light conditions in a clean greenhouse to avoid extraneous whiteflies and natural enemies. To obtain wild-type virgin adults, outdoor-grown eggplant leaves with late fourth instar nymphs were collected from the field one day prior to adult emergence. Virgin adults were collected as they emerged from their nymphal exuviae and held in gelatin capsules, then sorted into male and female groups using a microscope. Single males and females were then randomly paired within an 8 cm2 clip cage attached to whitefly-free cantaloupe plants. In the initial study, a cohort of 36 pairs were followed over a 24 day period. Serial transfers of the parental pairs were carried out on six different leaves per pair. Once attached, clip cages remained in position on leaves through courtship and copulation, egg laying and hatching, immature development and emergence of the progeny adults. Collections of emerged adults were made each day over a 30 day emergence period and daily proportions of sons and daughters were determined under a microscope for each parental female. In a follow-up study, access to males was experimentally manipulated using five different treatments involving period and duration of exposure to males over the course of female lifetimes. Of the initial 36 pairs started in the first experiment, 11 pairs remained intact for the 24 day duration. Eight of the 11 pairs produced total brood female proportions ranging from 0.59 to 0.77, while the other three pairs produced sex ratios near parity. Five of the eleven pairs produced total adult progeny that exceeded 500, and all but one pair had total adult progeny in excess of 400. The highest number of progeny produced was 608; mean progeny for all 11 pairs was 439. Of the remaining pairs of the original 36, surviving females that lost mates soon began laying unfertilized eggs based on records of subsequent adult emergence. The decline of female progeny was quite rapid and usually complete within three days following loss of the male. These observations were substantiated in the second experiment in treatments that involved isolation of females following a period of coupling with a male. In contrast, females initially isolated from males produced only male progeny until being paired with a mate, then began producing female progeny within one day of being paired; only male progeny were produced by females that remained unpaired for the duration of the experiment. These results demonstrate that males must be available for repeated copulations throughout the lifetime of females for egg fertilization to occur. Rapid shifts in sex ratio in both greenhouse colonies and field infestations may occur according to the availability of males for mating.

# Sub article: Relationship of Bemisia tabaci Adult Mortalities and Population Control to Imidacloprid Concentrations in Cantaloupes

**DOI:** 10.1673/031.008.0421

**Published:** 2008-01

**Authors:** S. J. Castle, J. C. Palumbo

**Affiliations:** ^1^ USDA, ARS Arid-Land Agricultural Research Center, Maricopa, AZ, USA.; ^2^ Department of Entomology, University of Arizona, Yuma Agricultural Center, Yuma, AZ, USA

Decision-making in chemical pest management typically depends upon information concerning how well a candidate pesticide performs against a particular pest species in a particular crop environment. The principal source of such information has traditionally been accumulated field efficacy data produced under variable trial circumstances, often outside the experimenter's control. Factors such as the type and age of the experimental crop, the degree of pest pressure from resident and immigrant populations, the level of natural mortality due to biotic and abiotic sources, etc., all potentially influence the outcome of each experimental trial. Thus, efficacy profiles for insecticides usually depend upon a consensus evaluation of individual trials conducted over a broad range of conditions. The process of consensus is a practical phenomenon whereby experimental field trial results are combined with the on-farm experiences of consultants and growers to arrive at a general and informal ranking of insecticides and how each performs against particular pest and crop combinations. While this system of evaluating insecticide efficacies has provided pest managers with rough guidelines on what works best in a given situation, it has done little towards identifying the activity profile of an insecticide in a crop over time. Greater confidence in the fate of an application might help reduce ‘insurance’ treatments that pest managers are sometimes compelled to apply because of uncertainty about persistence in the crop and level of control being exerted on a target population. The commercial availability of ELISA kits for quantifying residues of imidacloprid and thiamethoxam has made it possible to monitor titers of each of these systemic insecticides in plant tissues and extracted fluids. Quantification of residues provides a direct measure of activity within a plant, in contrast to indirect measures, such as monitoring insect densities post treatment. In the case of spring and fall cantaloupes grown in Arizona and California, titers of imidacloprid and thiamethoxam were measured in leaf tissue collected from plants in different stages of growth and under various treatment regimes. One of the choices facing growers each spring is whether to apply only one application at planting, as a sidedress, or as split applications. Results from two consecutive spring trials showed that significantly higher and more persistent titers of imidacloprid were attained when a single application was made at planting, compared to a full sidedress or split treatments. Much higher titers (> 10-fold) occurred in older, fully expanded leaves, compared to younger, growing leaves. Mean titers remained moderately high up to six weeks after planting, but then declined to a level where *Bemisia tabaci* nymphs began to establish. The relationship of imidacloprid titers to mortality of *B. tabaci* was further investigated by conducting in-field bioassays of adults attached to leaves with clip cages. Mortality was scored after 48 h, then leaf punches taken from the location where clip cages had been attached. Although adult mortality was generally low in each set of bioassays conducted, even when imidacloprid titers were high in leaves, nymphal densities remained low until later in the season. Differential mortality between adults and nymphs may be the reason why nymphs are controlled in the field at the same time that adults do not readily die. Sub-lethal effects, however, may prevent adults from actively feeding and ovipositioning on treated plants.

# Sub article: Bemisia tabaci nymphal feeding pathway in cotton

**DOI:** 10.1673/031.008.0422

**Published:** 2008-01

**Authors:** Chu Chang-Chi, Thomas P. Freeman, James S. Buckner, Thomas J. Henneberry

**Affiliations:** ^1^ USDA, ARS, Arid-Land Agricultural Research Center, Maricopa, Arizona, USA.; ^2^ Electron Microscopy Center, North Dakota State University, Fargo, North Dakota, USA; ^3^ USDA, ARS, Biosciences Research Laboratory, Fargo, North Dakota, USA

We are using brightfield light microscopy, differential interference contrast microscopy, confocal scanning light microscopy, scanning electron microscopy and transmission electron microscopy to investigate the feeding pathways of sweetpotato whitefly nymphs in cotton leaves. Microscopy events trace the insect's stylet penetrations from the initial entrance in the abaxial epidermal leaf surface and thereafter through the various leaf structures, as evidenced by the salivary sheaths, to the phloem tissue where feeding occurs. The morphology of the salivary sheath pathways between and through leaf cells was recorded with photographic images. The results indicate that salivary sheaths appear present and within the phloem tissue sieve tubes. Sweetpotato whitefly nymphs may move their stylets to feed in different sieve tubes. Continuing studies are being made to determine if and how the salivary sheaths attach to individual sieve tubes and the mechanisms of penetration of sweetpotato whitefly nymph stylets into phloem tissues during feedings.

# Sub article: Squash Leaf Curl Virus Localizes in Primary Salivary Gland Compartments, and at Midgut and Filter Chamber Brush Border Cells In Viruliferous Bemisia tabaci

**DOI:** 10.1673/031.008.0423

**Published:** 2008-01

**Authors:** J. M. Cicero, J. K. Brown

**Affiliations:** Department of Plant Sciences, The University of Arizona, Tucson, AZ, USA.

*Bemisia tabaci* acquires begomovirus (genus *Begomovirus*, family *Geminiviridae*) particles during feeding on the phloem sap of virus-infected plants. Virions follow a persistent, circulative pathway in the whitefly vector's body to reach the primary salivary glands, which upon gaining entrance, are transmitted to another host plant in the salivary contents that are egested during feeding. However, this circulative route, or ‘the transmission pathway’ is not well understood. Knowledge of the specific pathway of virons and of the relationship with the principle whitefly vector organs that comprise the transmission pathway are important requisites to identifying and characterizing interactive whitefly- and presumably endosymbiont-encoded proteins essential to the transmission process and the basis for virus-vector specificity. The alimentary canal of *B. tabaci* and other Homoptera is modified to accommodate the high concentration of water in its' food sources. Anterior and posterior ends of the midgut are connected to each other in the anterior abdominal hemocoel. Housed within the posterior end, where the hindgut starts, is a minute, conical, filter organ. The oesophagus complexes with, and empties directly into, this organ rather than the primitive location at the anterior midgut. Also associated with this complex are cells interpreted to be malpighian. The filter organ separates food from water, stores the food in the midgut loop, and allows the water to drain directly down the hindgut so that it doesn't dilute the blood or the food reserves as in other fluid-feeding insects. From *in vivo* observations and huge numbers of instances in dissections and sectioned material, the midgut loop was found capable of passing from the abdomen, into the thorax, and back again. When in the thorax, it can reach the base of the head, where the salivary glands are located. *B. tabaci* has two pairs of salivary glands, primary and accessory, tethered to each other by their canals, which in turn are joined to common ducts that empty into the buccal cavity. In this study, *in-situ* hybridization with digoxygenin-labelled oligonucleotide viral DNA probes localized virus DNA in whole viruliferous whiteflies embedded in paraffin sections, revealing segments of the circulative pathway that have been pointed to by previous studies, were not entirely confirmed, or were altogether unknown. In this study, heavy deposits of silver-enhanced, ultrasmall gold-conjugated-anti-digoxygenin were observed on the microvilli, whereas more sparse deposits were observed in the cytoplasm and along the basement membrane of the midgut and filter chamber for virtually all specimens examined. Heavy deposition also occurred in two lobate, probably cellular, compartments at the apex of each primary salivary gland, at the same general region where the salivary canals are adjoined. Labeling of the analogous locations was not observed in identically treated, nonviruliferous adult whiteflies. Even though the accessory gland has long been thought to serve a key function in the pathway and in virus-vector specificity, no analogous labeling was seen in the accessory glands that could be considered above the threshold of sensitivity when using this approach to localize virus DNA in the whitefly. In addition to aortal-directed circulation, we posit that hemolymph is reciprocally displaced, volume for volume, whenever the midgut translocates from abdomen to thorax and back again, and that this action therefore assists in the diffusion of blood-borne virions. Further, virions might also be transported directly to the salivary gland membrane from contact with the midgut when it is anteriorized into the thorax. In contrast to the aphid-luteovirus model, these results suggest that the primary glands, instead of the accessory glands are organs in which virions are concentrated while awaiting discharge in salivary contents during subsequent feeding and egestion.

# Sub article: Analysis of Bemisia tabaci gene expression using Drosophila microarrays

**DOI:** 10.1673/031.008.0424

**Published:** 2008-01

**Authors:** Henryk Czosnek, Murad Ghanim

**Affiliations:** ^1^ Faculty of Agriculture, The Hebrew University of Jerusalem, Rehovot, Israel.; ^2^ Institute of Plant protection, Department of Entomology, the Volcani Center, Bet Dagan, Israel

The Whitefly Functional Genomics Project has generated cDNA libraries from viruliferous and non-viruliferous adult whiteflies and from the insect developing stages. A microarray has been generated which contains 6,000 spots representing 4,860 contigs and singletons. We have started to use this platform to study gene expression during insect development and begomovirus vectoriality. However, since we estimate that the number of genes represented on the array is approximately one third to one forth of the genes of *B. tabaci*, the use of this platform may be limited. Hence we wondered whether the *Drosophila* microarray might be of use in studying whitefly gene expression. In recent years, many genomic projects took the advantage of an existing microarray platform of one organism to investigate the transcriptome of related organisms (cross-species hybridizations). We have used a microarray representing the entire *D. melanogaster* genome and compared RNA samples derived from the whitefly *Bemisia tabaci* to RNA derived from *D. melanogaster*. We found that a relatively large number of the *Drosophila* genes represented on the microarray hybridized with whitefly RNA samples. By comparing some of the genes, which hybridized to the *D. melanogaster* microarray, we were able to identify previously annotated genes in the whitefly. We have compared the expression of whitefly genes from four distinct developmental stages that hybridized to a RNA mixture extracted from *D. melanogaster* across developmental stages used as standard. As expected not all genes y were expressed in *B. tabaci* the same way they behaved in *Drosophila*. In *Drosophila* a very high transcriptional activity occurs during the oogenesis and embryogenesis stages; more than 85% of the *Drosophila* genes were expressed during the first 22 hours of the embryo development. By comparison, 2,378 spots on the *Drosophila* microarray hybridized with RNA from *B. tabaci* egg, compared with 1,744 spots with RNA from larvae, 1610 spots with RNA from pupae and 1,684 spots with RNA from adult. These results demonstrated that besides the conservation in sequence, which allowed hybridization, conservation in the temporal expression of some of the genes identified across development was also observed. Similarly heterologous hybridizations allowed discovering whitefly genes involved in stress response accompanying acquisition and retention of begomoviruses. In conclusion, in the absence of a microarray that represents the complete genome of *Bemisia tabaci*, one can extract valuable information on the biology of this inset by hybridizing whitefly RNA to the *Drosophila* microarray.

# Sub article: Limited but persistent genetic differentiation among biotype Q of Bemisia tabaci, the only biotype detected in protected crops in Southern France

**DOI:** 10.1673/031.008.0425

**Published:** 2008-01

**Authors:** A. Dalmon, F. Halkett, M. Granier, M. Peterschmitt

**Affiliations:** ^1^ INRA-LNPV, Plant Pathology Department, Montfavet, France.; ^2^ CIRAD, UMR BGPI, Montpellier, France

*Bemisia tabaci* is a major pest in glasshouses in Southern France. It was first reported in the 80ies and population levels increased highly in 2001. At the same time TYLCV was reported for the first time, and subsequently several criniviruses were characterized in greenhouse crops. Genetic diversity among *B. tabaci* French populations was characterized, and gene flow in vector populations was studied. Eighteen populations were collected in glasshouses in Southern France from 2003 to 2005, mainly from tomato crops, but also from other vegetables and ornamentals. One population was collected on Poinsettia in a botanical garden. The 19 populations (520 female individuals) were studied with seven independent microsatellite markers, which revealed a high genetic diversity. Within populations, some heterozygote deficiency was observed, but *F*IS values were not significantly different from 0. Four groups were distinguished using a Bayesian clustering method (Structure software). One group limited to the botanical garden population consisted of biotype B individuals according to nucleotide sequences of cytochrome oxidase 1 gene. Despite a mixed ancestry derived from the three other groups, all individuals of the 18 crop populations were inferred to belong to biotype Q. This is the first detection of biotype Q in France. Its predominance was unexpected because only biotype B was previously detected based on silver leaf symptoms (Villevielle and Lecoq, 1992, Phytoma 440). No geographic structuring was observed among the 18 crop populations with AMOVA test (after pooling 2003 populations into 3 main geographic areas) and isolation by distance analysis, suggesting a recent colonization event of a single origin and/or large-scale migration events despite of isolation due to glasshouse protection. When all data were pooled according to the host species sampled, a host selection effect was tentatively noted for individual sampled from hibiscus.Genetic diversity was studied over time for *B. tabaci* populations collected two or three times in the same glasshouse from 2003 to 2005. A genetic differentiation process was highlighted in one of the four repeatedly sampled glasshouses through a decrease of allelic richness and observed heterozygosity in the *B. tabaci* populations collected yearly from 2003 to 2005. The populations from this glasshouse were the sole to differentiate among biotype Q. It is supposed that this glasshouse population was sufficiently isolated from other populations and has undergone sufficiently severe reductions of population size during host free periods to exhibit such a drift.

# Sub article: Bemisia tabaci, a top 100 invader

**DOI:** 10.1673/031.008.0426

**Published:** 2008-01

**Authors:** Paul De Barro

**Affiliations:** CSIRO Entomology, Queensland, Australia.

Invasive species are considered by the IUCN to be amongst the top three threats to biodiversity and the structure and function of agricultural ecosystems. One of these, *Bemisia tabaci* is grouped in the GISP One Hundred of the World's Worst Invasive Alien Species. Since the 1980s one *Bemisia tabaci*, biotype B, has been particularly successful at invading and establishing to the extent that it has extended its range from the region of the Middle East/Asia Minor to all continents except Antarctica. Yet B is not the only example of a successful *B. tabaci* invader. Here, the history of past *B. tabaci* invasions is discussed in the context of their impact on the global genetic structure of the complex. Possible mechanisms such as mating interference and competition for resources are described and their possible roles as contributing factors to the patterns observed discussed. A recent invasion in Asia by a non-B *B. tabaci* is described and its impact on local economies discussed.

# Sub article: A Molecular Phylogeny of Indo-Australian Aleyrodidae

**DOI:** 10.1673/031.008.0427

**Published:** 2008-01

**Authors:** Paul DeBarro, Marc Coombs, Rosemarie Rosell, Heather Costa, Donald R. Frohlich

**Affiliations:** ^1^ CSIRO, Brisbane, Queensland, Australia; ^2^ Biology Department, University of St. Thomas, Houston, Texas, USA.; ^3^ Entomology Department, University of California, Riverside, California, USA

Two nuclear DNA sequences (D2 and 18S) were used to reconstruct the phylogeny of 80 species in over 40 Indo-Australian Aleyrodid genera. Four methods were applied: distance, parsimony, maximum likelihood, and a bayesian technique. Herein we discuss relationships between the genera and compare the utility and differences of the four analytic approaches with relationships based on morphological characteristics of puparia.

# Sub article: Insecticide Resistance in Bemisia tabaci: A Global Perspective

**DOI:** 10.1673/031.008.0428

**Published:** 2008-01

**Authors:** Ian Denholm, Kevin German, Martin Williamson

**Affiliations:** Rothamsted Research, Harpenden, Hertfordshire, England.

Current management strategies for *Bemisia tabaci* are severely constrained by the propensity of these species to develop resistance to insecticides. The breadth and geographical extent and breadth of resistance continue to increase, and many novel compounds are now affected in addition to chemical classes used widely in the past. Factors likely to promote the build-up and spread of resistance include (i) a haplodiploid breeding system enabling the rapid selection and fixation of resistance genes (ii) its breeding cycle on a succession of treated host plants; and (iii) its occurrence on high value crops that are widely traded internationally. There has been substantial progress with resolving the mechanistic basis of many forms of resistance, but others remain poorly characterised, other than at a phenotypic level. This paper will provide an update on the known incidence of resistance in *B. tabaci*, its practical consequences, and available information on the mechanisms responsible and their cross-resistance implications. As mechanisms become better understood, there is exciting scope for investigating the homology of resistance traits and genes between potentially reproductively-isolated biotypes, and the possible role of resistance in influencing changes in biotype composition. In this context, steps to expand the range of genomics-related tools applicable to *B. tabaci* are very welcome. These will provide valuable additional resources for the resistance research community.

# Sub article: Management of Bemisia Resistance: Cotton in the Southwestern USA

**DOI:** 10.1673/031.008.0429

**Published:** 2008-01

**Authors:** T. J. Dennehy, B. DeGain, G. Harpold, X. Li, D.W. Crowder, Y. Carrière, P. C. Ellsworth, R. L. Nichols

**Affiliations:** ^1^ Department of Entomology, University of Arizona, Tucson, AZ, USA.; ^2^ University of Arizona, Maricopa Agricultural Center, Maricopa, AZ, USA; ^3^ Cotton Incorporated, Cary, NC, USA

*Bemisia tabaci* can be a severe pest of many crops produced in arid regions of the southwestern United States including cotton, melons and vegetables. It has also become an increasingly common problem in glasshouse production systems. Attempts to control whiteflies with conventional broad-spectrum insecticides have had devastating results in many desert agro-ecosystems. Severely reduced natural enemy populations have been associated with resurgences of whiteflies, outbreaks of secondary pests, and rapid evolution of pest resistance. Under such conditions, *B. tabaci* has developed resistance to essentially all insecticides to which it has been repeatedly exposed. Such was the case in 1995, when whitefly numbers reached crisis proportions in Arizona cotton despite application of 6 to 15 insecticide treatments per acre. In consultation with researchers in Israel and the United Kingdom, emergency alternatives for whitefly control were formulated and implemented in 1996 that replaced broad-spectrum insecticides during the early season with once-per-season use of the insect growth regulators, pyriproxyfen and buprofezin. Concomitant registration of Bt cotton significantly reduced treatments of conventional insecticides for lepidopteran pests. Additionally, neonicotinoid insecticides provided exceptional whitefly suppression in the other major whitefly hosts, melons and winter vegetables. The end result has been over a decade of unprecedented low insecticide use in cotton, and equally unprecedented effectiveness of biological control in cotton fields. Management of *Bemisia* resistance in the Southwestern USA is focused intensively on sustaining effective, selective insecticides. This includes statewide detection and isolation of resistance in cotton, vegetables, melons and glasshouses, and collaborative research to characterize critical toxicological, genetic and ecological parameters of resistance in laboratory and field experiments. I will overview new developments from this research, including information regarding the distribution and threat posed by Q biotype.

# Sub article: Novel Measurement of Group Adoption of IPM in Diverse Cropping Communities

**DOI:** 10.1673/031.008.0430

**Published:** 2008-01

**Authors:** P. C. Ellsworth, J. C. Palumbo, A. Fournier, Y. Carriere, C. Ellers-Kirk

**Affiliations:** ^1^ Department of Entomology, University of Arizona, Tucson, AZ, USA.; ^2^ Maricopa Agricultural Center, University of Arizona, Maricopa, AZ, USA

Integrated pest management (IPM) in the sensitive environments of the desert Southwest is vulnerable to the destabilizing impact of mobile polyphagous pests that are capable of attacking winter vegetables, melons, and cotton -- most notably, the silverleaf whitefly (*Bemisia tabaci*). Because of the year-round growing season present in desert cropping systems and the chronic nature of pest incidence, emphases are needed on area-wide reduction of pest populations through all means possible. This has led to the development of IPM programs for these crops that emphasize selective and other reduced-risk technologies, including insect growth regulators (IGRs) and neonicotinoid insecticides for the control of pests. To preserve these valuable IPM tactics — by protecting them from resistance — we have developed IPM guidelines for cross-commodity management of whiteflies that transcend field or grower borders and depend on group adoption over large areas, to be effective in area-wide source reduction and in proactive resistance management for major reduced-risk technologies (IGRs and neonicotinoids). We have developed a novel approach for measuring spatially-relevant adoption of our IPM guidelines. This new analytical approach utilizes pesticide-use reporting data and GIS/GPS technology to understand area-wide adoption of the cross-commodity guidelines and to further guide future research, technology transfer, and outreach efforts. This technique allows us to evaluate IPM and its implementation and adoption, to a level of organization that spans multiple crops and pests over entire ecosystems. Our model system of analysis should be broadly applicable to the measurement and improvement of IPM systems worldwide.

# Sub article: Ultrastructural Characteristics of Bemisia Adult and Nymph Feeding

**DOI:** 10.1673/031.008.0431

**Published:** 2008-01

**Authors:** Thomas. P. Freeman, James. S. Buckner, Chu Chang-Chi, Scott A. Payne, Jayma A. Moore

**Affiliations:** ^1^ Electron Microscopy Center, North Dakota State University, Fargo, North Dakota, USA.; ^2^ Biosciences Research Laboratory, USDA-ARS, Fargo, North Dakota, USA; ^3^ USDA-ARS, Arid-Land Agricultural Research Center, Maricopa, Arizona, USA

Included among the more than 1200 known species of whiteflies are important agricultural pests that can cause significant economic loss for a wide variety of field and horticultural crops. Damage to host plants can be a direct result of obligate phloem feeding, whitefly transmission of virus or non-viral plant disorders, or fungal growth associated with the presence of honeydew. Both adults and nymphs feed on the abaxial surfaces of leaves, and host-plant selection affects egg-hatch success and nymph survival. Important factors associated with successful feeding include stylet length and salivary sheath development. The mouthparts of adult whiteflies are typical of the Homoptera. Whiteflies have a triangular labrum covered with hairs, and a four-segmented labium. The first segment is interpreted as an extension of the thorax. Segments two, three, and four are characterized by a deep labial groove. The stylet bundle enters the labial groove between the first and second labial segments. When the insect is not feeding, the stylet is completely contained within the labium. The stylet bundle consists of two external mandibular stylets and two interlocking maxillary stylets forming separate food and salivary canals. Mandibular and maxillary protractor muscles probably play a role in directional movements of the stylets and perhaps have a limited role in stylet advancement. Adult feeding sites occur both along the margins and through the central portions of leaf abaxial epidermal cells. Both initial probe sites and successful feeding sites can be identified easily by the presence of a glue-like deposit where the tip of the labium was anchored to the host plant epidermis. The mechanical force required for both stylet insertion and withdrawal results from changes in the position of the whitefly head in relation to the anchored labium during feeding. The total length of the stylets and the portion of the stylets inserted into the host plant leaf can be measured easily by examining the position where the stylet bundle is inserted into the labial sheath. During penetration of the host leaf, the whitefly forms a salivary sheath. This sheath is formed by the production of a small bead of material that emanates from the stylet tip, hardens, and is then penetrated by the stylet bundle. Additional beads are formed, giving the sheath a segmented or beaded appearance. The salivary sheath leaves a permanent record of stylet travel from the epidermis to the phloem tissue. Stylets usually do not penetrate directly from the epidermis to the phloem tissue, and salivary sheaths are frequently highly branched as they wind their way through the intercellular spaces of the leaf mesophyll. Branching characteristics of both adult and nymph salivary sheaths provide clear evidence that the stylets can be partially withdrawn and reinserted. Nymphs feed during all developmental instars. The stylets of early nymphal stages are generally shorter that those of adults but they are still long enough to reach the phloem tissue of the host plant. Although the stylets are longer with each new instar, they do not lengthen in direct proportion to the increase in body length. The rostrum on the nymph is short and has a groove similar to the groove on the adult labium through which the stylet bundle slides. In the nymph, unlike the adult whitefly, it is difficult to determine the portion of the stylet used in feeding, as the rostrum and stylet insertion point are below the nymph. Stylets found in all exuviae confirm that the stylets are withdrawn prior to molting and that new stylets must be formed and inserted following the molt. Photographs obtained using light microscopy, confocal microscopy, scanning electron microscopy and transmission electron microscopy will be presented to document whitefly adult and nymph feeding characteristics including the mechanism of stylet penetration, salivary sheath formation, the course of stylets through the host plants, and a detailed anatomical and morphological description of whitefly adults and nymphs.

# Sub article: Interaction between Two Entomopathogenic Fungi with the Parasitoid Encarsia formosa Gahan in the Greenhouse Whitefly Control

**DOI:** 10.1673/031.008.0432

**Published:** 2008-01

**Authors:** Félix García-Valente, L. D. Ortega-Arenas, H. González-Hernández, F. Tamayo-Mejía, A. Guzmán-Franco

**Affiliations:** ^1^ Programa en Entomología, Campus Montecillo, Colegio de Postgraduados, Montecillo Estado de México, México.; ^2^ Sanidad Vegetal, SAGARPA, Celaya, Guanajuato, México

Interaction of the entomogenous fungi *Paecilomyces fumosoroseus* and *Beauveria bassiana* with the parasitoid *Encarsia formosa*, all natural enemies of whiteflies, was studied under greenhouse conditions. Second instar nymphs of the whitefly *Trialeurodes vaporariorum* were treated with the entomopathogens and later exposed to parasitoid females at different intervals of time. With the combination *P. fumosoroseus-E. formosa* nymphl mortality decreased with increases in time between application of the fungus and the release of the parasitoids (66.9% to 49.3%, 3 days later). In contrast, the combination *B. bassiana* - *E. formosa* showed that mortality increased as the time interval increased (52.6% to 61.9%, 3 days later). In both cases, mortality from the effect of the two control agents was greater than that caused by either of the natural enemies acting independently. In a second experiment, each fungus was applied to whitefly nymphs previously exposed to *E. formosa*. The greatest mortality was observed when fungi were applied 2 days after nymphs were exposed to the action of the parasitoids (75.4% in the combination *E. formosa - P. fumosoroseus*, and 84.5% in the combination *E. formosa - B. bassiana*). Mortality decreased as the time between parasite exposure and infection increased. The results suggest that the combination of *E. formosa* and one of the two entomopathogenic fungi has potential use in the control of *T. vaporariorum* nymphs under greenhouse conditions.

# Sub article: Microsatellite Markers in the Entomopathogenic Fungus Paecilomyces fumosoroseus for Monitoring of Isolates Introduced against Bemisia tabaci, Epidemiological and Population Genetics Studies

**DOI:** 10.1673/031.008.0433

**Published:** 2008-01

**Authors:** Nathalie Gauthier, Bon Marie-Claude, Cécile Dalleau-Clouet, Jacques Fargues

**Affiliations:** ^1^ Campus International de Baillarguet, Centre de Biologie et de Gestion des Populations, Institut de Recherche pour le Développement (IRD), Montferrier sur Lez, France.; ^2^ European Biological Control Laboratory, USDA-ARS, Montferrier sur Lez, France; ^3^ Campus International de Baillarguet, Centre de Biologie et de Gestion des Populations, INRA, Montferrier sur Lez, France

One of the greatest challenges for improving the use of fungi, not only as microbial control agents but also for all epidemiological and population genetics studies, is the ablity to identify the isolates used and analyzed. The haploid entomopathogenic hyphomycete *Paecilomyces fumosoroseus* (*Pfr*) used for biological control of economically serious agricultural pests is a geographically widespread fungus infecting various orders of insects. In particular, it is naturally associated with the whitefly *Bemisia tabaci* (Homoptera Aleyrodidae) in various geoclimatic regions. Ongoing difficulties in applying morphological approaches to isolate-recognition *Pfr* have spurred the search for discriminant markers. Based on rDNA-ITS RFLP approach, all isolates collected from *B. tabaci* clustered in 1 group although they originated from diverse geographical locations. Microsatellite markers were therefore developed and characterized in *Pfr*. Upon the 9 isolated, 8 were revealed as polymorphic. Amplification success and length and sequence variability of these markers were investigated on a set of 34 *Pfr* sampled from various insect-host species and geographical locations. First, PCR amplifications at the 9 previously designed loci were only successful with *Pfr* from Homoptera hosts (25 *B. tabaci* and 1 *Phenacoccus sp.* hosts) which has suggested existence of genotype host-associations. Second, allele size and above all sequence analyses (flanking microsatellite-regions and/or microsatellite regions) improve our ability to discriminatamong *Pfr* isolates and provide a means to subdivide isolates obtained from *B. tabaci*. Indeed, from a unique *Pfr* rDNA-ITS RFLP haplotype, 7 patterns based on specific microsatellite allelic-sizes and 14 haplotypes based on their sequencing were resolved from *B. tabaci* which emphasizes their value for further epidemiological studies and use in biological control. Finally, their value for genetic relationships inference was also proven and a phylogeographic framework was henceforth established providing evidence for two distinct lineages of *Pfr*, American-Cuban and Asian, with a putative Indian origin of the American-Cuban *Pfr* group from an Indian group. Consequently, the microsatellite markers described represent a potent tool for *Pfr* population diversity studies and open a new and informative window on the use of well-discriminated isolates in epidemiology and biological control strategy against *B. tabaci*.

# Sub article: Novel Candidates for the Development of Biopesticides to Control Whitefly Pests

**DOI:** 10.1673/031.008.0434

**Published:** 2008-01

**Authors:** Dale B. Gelman, Jing S. Hu, Phyllis A. W. Martin, Michael B. Blackburn, Michael E. Salvucci

**Affiliations:** ^1^ Insect Biocontrol Laboratory, USDA, ARS. Beltsville, MD, USA.; ^2^ Western Cotton Research Laboratory, USDA, ARS, Phoenix, AZ, USA

The sweet potato whitefly, *Bemisia tabaci* (Biotype B) causes hundreds of millions of dollars in crop losses each year both in field and greenhouse settings. Reports of pesticide resistance in whiteflies and environmental concerns related to pesticide usage have made the reduction of pesticide application a primary goal for agriculture and have increased the emphasis on the use of cost-effective biological control strategies and insect specific biopesticides in IPM programs. Characteristics associated with the designation biopesticide include: greater safety than conventional pesticides and usually no residue problems. The two classes of biopesticides are biochemical and microbial. The use of biochemical and bacterially-produced insecticidal compounds as control agents for whiteflies and other sucking insects are worthwhile alternative strategies that should be examined. Recently, several monosaccharides as well as toxins produced by *Photorhabdus luminescens* and a newly discovered bacterium, *Chromobacterium substugae*, were found to be toxic to whiteflies. Arabinose, mannose, ribose and xylose, when added to artificial diet, were found to kill sweet potato whitefly (SPWF) nymphs and adults. Results showed that there was no substantial inhibition of alpha glucosidase (converts sucrose to glucose and fructose) or trehalulose synthase (converts sucrose to trehalulose) activity by arabinose, mannose or xylose. When adult *B. tabaci* were fed for 24 h on diets containing U- ^14^C sucrose, L-[methyl-^3^H] methionine or inulin-[^14^C]-carboxylic acid, and one or none (control) of the toxic sugars, the four insecticidal sugars, significantly reduced (by 70 – 80%) the amount of radioactivity incorporated and excreted in honeydew. Thus, we conclude that the four sugars act as antifeedants. When U- ^14^C sucrose and either arabinose, xylose, or mannose were fed to *B. tabaci* adults, less radiolabeled carbon dioxide was excreted; thus, respiration also appears to be reduced. Interestingly, melibiose and trehalose, two sugars that are not toxic to whiteflies, also reduced (by 30 – 40%) U- ^14^C sucrose uptake but not carbon dioxide release. In other experiments, a high molecular weight insecticidal protein complex (Tca) produced by the entomopathogenic bacterium P. luminescens, has been reported to be orally toxic to *B. tabaci*. When artificially fed Tca, newly emerged nymphs developed poorly, or not at all. Tca concentrations of between 0.1 and 0.2 ppm reduced the number of nymphs reaching the second instar by 50%. In addition, a preparation of Tca missing two prominent subunits, TcaAii and TcaAiii, was found to be at least as toxic to *B. tabaci* nymphs as Tca itself, indicating that the activity of Tca is not dependant on the presence of these subunits at the time of ingestion. Tca (-Aii and Aiii) administered at 4 ppm to adult whiteflies also reduced mean day survival from 6.7 to 2.6 days. In addition, a recently discovered species of *Chromobacterium* (*C. subtsugae*) was found to produce toxins that are insecticidal to whiteflies and other insect pest species. When fed on a diet containing 10% of a *C. subtsugae* extract, the number of 2^nd^, 3^rd^ and 4^th^ instar nymphs and emerged adults was significantly lower (15 – 34%) in experimental groups as compared to control groups. When adult *B. tabaci* were fed on artificial diet containing 5% of a cell-free bacterial preparation, 100% mortality was observed after 4 days. Upon fractionation through molecular weight cut-off filters, activity was present in the 3–10 kDa and greater than 300 kDa fractions. Thus, data support the existence of at least two toxins, and although the identities of these toxins have not yet been determined, results show that the toxins are not protein in nature.

# Sub article: Bemisia tabaci research: Past -present — future

**DOI:** 10.1673/031.008.0435

**Published:** 2008-01

**Authors:** Dan Gerling, Paul DeBarro

**Affiliations:** ^1^ Tel Aviv University, Department of Zoology, Israel.; ^2^ CSIRO Division of Entomology, Australia

*Bemisia tabaci* was described as a pest in 1889 and has maintained that status ever since. Damage ranges from direct plant sucking to honeydew production and plant contamination through virus transmission. Research followed damage-related developments. We review it in relation to the pest's identity, plant relationships, natural enemies and management, pointing out past research directions and possible directions for future research. Principal research on the identity of *B. tabaci* began with the recognition of more than one biotype differing in life history parameters, host plant associations, plant-related damage and insecticide resistance. Following mapping of the world situation, we have reached a phase in which finer meanings of the biotype identity can serve as landmarks for their affinity and pestiferous characteristics. Plant relationships, combined with the study of insect behavior, probably constitute the most meaningful subject on which research can bring about better management of *B. tabaci*. The discovery that plant metabolism is manipulated by whiteflies and the existence and action of PR proteins and of other plant defensive chemicals, are landmarks that should be followed. Their mode of action should be better understood and serve as bases for future plant manipulation and breeding. The initial production of plant stimulators and their utilization for overcoming whitefly-caused problems are steps in this direction. Study of ecological parameters such as influences of field sizes and shapes and the behavior of the organisms involved, may also help to improve whitefly management. Natural enemy exploitation has moved from utilization of spontaneous whitefly decimation by local fungi, parasitoids and predators through the manipulation of available biological control agents to the screening and selection of globally occurring organisms. This has provided excellent greenhouse control and should encompass more of the available unstudied organisms and broaden its scope to all facets of *B. tabaci* pest conditions. Natural enemy utilization should take into account the host plant relationships as well as the available insecticidal solutions. Controls strategies presently take into account the available enemies and insecticides. Plant resistance-inducing materials and plant stimulators are just coming into use. With proper research, the integration of molecular technology, proper plant breeding and utilization of additional natural enemies, better *B. tabaci* management can be achieved.

# Sub article: Distribution of Secondary Symbionts in Israeli Populations of Bemisia tabaci

**DOI:** 10.1673/031.008.0436

**Published:** 2008-01

**Authors:** Murad Ghanim, Yuval Gottlieb, Elad Chiel, Einat Zchori-Fein

**Affiliations:** ^1^ Institute of Plant Protection, Department of Entomology, the Volcani Center, Bet Dagan, Israel.; ^2^ Department of Evolutionary and Environmental Biology, University of Haifa, Haifa; ^3^ Laboratory of Genetics, Newe-Ya'ar Research Center, ARO, Ramat-Yishai

Whiteflies are sap-sucking insects that harbor the primary symbiont *Portiera aleyrodidarum* as well as a diverse facultative microbial community. The sweetpotato whitefly *Bemisia tabaci* (Gennadius) is actually a species complex consisting of many biotypes which may differ in characters such as host range, insecticide-resistance and ability to transmit plant viruses. All Israeli *B. tabaci* tested to date belong to either the B or the Q biotype. In this work we have studied the bacterial composition of several laboratory and field populations collected between 1987 and 2005 from a variety of host plants and geographic regions in Israel. Beside *Portiera*, this community was found to be composed of four secondary symbionts: The B biotype harbors exclusively *Hamiltonella*, while the Q biotype harbors exclusively *Arsenophonus*, and *Wolbachia*. Both biotypes harbor *Rickettsia*. The localization of *Rickettsia*, and *Hamiltonella* in *B. tabaci* eggs, nymphs and adults was studied using fluorescence in-situ hybridization. This analysis revealed a unique concentration of *Rickettsia* around the gut and follicle cells, as well as a random distribution in the haemolymph, excluding the bacteriomes. The unique distribution of *Rickettsia* may be related to the role it plays in the biology of the whitefly. *Hamiltonella* was found to be localized inside the bacteriocytes confined with *Portiera* during all developmental stages. We propose that the host's ability to thrive under diverse environmental conditions can be partially explained by correlation between the presence of various bacteria and *B. tabaci* biotype.

# Sub article: Gene expression in pyriproxyfen resistant Bemisia tabaci biotype

**DOI:** 10.1673/031.008.0437

**Published:** 2008-01

**Authors:** Murad Ghanim, Svetlana Kontsedalov

**Affiliations:** Institute of Plant Protection, Department of Entomology, the Volcani Center, Bet Dagan, Israel.

Pyriproxyfen is a biorational insecticide that acts as a juvenile hormone analog (JHA) and disrupts insect development with unknown molecular mode of action. This insecticide is one of the major insecticides being used to control the whitefly *Bemisia tabaci* (Gennadius), and is one of the major insecticides that comply with integrated pest management (IPM) programs, because of minimal effects on the environment, humans and beneficial organisms. During the last few years, resistance to pyriproxyfen has been observed in several locations in Israel; sometimes reaching thousand-fold increase in the resistance. The molecular basis underlying this resistance, which may lead to understanding the mode of action of pyriproxyfen, and developing molecular markers for rapid monitoring of resistance outbreaks, was not studied. In this communication, we used a recently developed cDNA microarray from *B. tabaci* to monitor changes in gene expression in resistant *B. tabaci* populations. Based on statistical analysis we identified 111 expressed sequence tags (ESTs) that were differentially regulated in the resistant strain. Although many of the regulated ESTs observed in our study belong to families usually associated with developed resistance and xenobiotic detoxification such as mitochondrial genes, P 450s and oxidative stress, many other genes associated with protein, lipid and carbohydrate metabolism, and genes related to JH associated processes in insects such as oocyte and egg development were also observed.

# Sub article: Can molecules solve the Bemisia conundrum when morphology cannot? A taxonomist's perspective

**DOI:** 10.1673/031.008.0438

**Published:** 2008-01

**Authors:** Raymond J. Gill

**Affiliations:** California Department of Food and Agriculture, Sacramento, CA, USA.

*Bemisia tabaci* has a number of synonyms produced partly due to the extreme plasticity of the pupal stages. And even though the mechanisms for the pupal variability are now largely understood, as are the limits of this variability within some generic groupings, we still do not understand the relationships of the general morphology of whiteflies to species limits, or even to generic limits. Early work by Laurence Mound and Louise Russell showed that whitefly pupal morphology could be morphologically altered by environmental factors at least in the genera *Bemisia* and *Trialeurodes*. Further studies have shown variable pupal morphology to be common in many other genera, at least within the whitefly subfamily Aleurodinae. Current molecular studies are beginning to shed some light on a few morphological structures that could be reliable generic and specific characters for some of the *Bemisia* species and related genera, by giving us a glimpse of evolutionary relationships that have not been visible to the classical taxonomist previously. Hopefully this research will give us some indication of which morphological characters are useful in this way, and those characters, which are not of importance. While there are no good morphological characters for separating the various races of *tabaci* (as currently understood), there are some interesting differences in biology, mating behavior, cross fertility and virus transmission. Are there cryptic species involved? Some interesting molecular data is emerging that suggests strongly that there could be, considering the close morphological similarities of *Bemisia formosana* and *graminus*, and some of the *Lipaleyrodes* species studied so far. Recent molecular work has given us some insights into the relationships of many of the variants (biotypes=strains=races, genetic variants) of the *Bemisia tabaci* complex. Several of the species that are very close to *tabaci* morphologically include *B. capitata; B. formosana* and *B. graminus* and these are being included in a *tabaci* complex of species. But as an example of what we need to learn is the relationships of some other species in other genera that may in fact belong in the genus *Bemisia* and may even be part of the *tabaci* complex, such as *Bemisiella, Lipaleyrodes*, some species of *Pealius* (i.e. *P. azaleae*), *and Parabemisia myricae*. And a few of the races of those species appearing to be related to *B. afer* are also open for question. It will be of interest to see whether other genera such as *Asterobemisia* and *Neobemisia* are actually part of the *B. afer* complex, or are actually part of the same species. There are some schools of thought that the *afer* complex of species including *tuberculata* in South America, *B. berbericola* in North America, *leakii* and *moringae* from India and others may all be the same species that has been spread by humans, as has *tabaci*. One of the most important pupal characteristics that occur in *Bemisia* is the morphological shape of the vasiform orifice and lingula. The vasiform orifice is open at the posterior end, and the lingula usually protrudes beyond the operculum. However, a number of other genera share these characteristics to some degree. So far, only molecular data have been able to clarify certain generic positions within the Aleyrodinae, as it relates to these characters. Clearly, much additional work is needed to achieve a robust understanding of *Bemisia*.

# Sub article: Pest Situation and Biological Control of Bemisia tabaci in Canary Islands

**DOI:** 10.1673/031.008.0439

**Published:** 2008-01

**Authors:** A. González, R. Martin, M.A. Reyes, I. Jiménez, V. Suárez, I. De Paz, I. Echevarría, A. Carnero, J.M. Rodríguez, E. Hernández — Suárez

**Affiliations:** ^1^ Plant Plant Protection Service, Gov.of Canary Islands, Spain; ^2^ Plant Protection Dpto, ICIA, Canary Islands, Spain.; ^3^ Agriculture Service of Cabildo, Gran Canaria, Spain

The Agriculture Service of the Canary Island Government released during 2005 and 2006 the parasitoids *Eretmocerus eremicus* and *E. mundus* and the adults of the predator *Nesidiocoris tenuis* in some localities on the islands of Tenerife and Fuerteventura, and in Gran Canaria on diverse horticultural crops (tomatoes, eggplant, zucchini, sweet pepper, green bean, and melon). The inundative release method used for these trials was in cooperation with the commercial firms Koppert and Biobest. Pest control was mainly obtained from the released natural enemies. The releases were applied to the crops and surroundings areas on pre-existing alternative host plants of *Bemisia tabaci*. Although the results to date have been variable, 85% parasitism has been recorded at some release sites.

# Sub article: Bemisia tabaci (Homoptera: Aleyrodidae) Instar Preference by the Parasitoids Eretmocerus mundus and Encarsia pergandiella (Hymenoptera: Aphelinidae)

**DOI:** 10.1673/031.008.0440

**Published:** 2008-01

**Authors:** S. M. Greenberg, W. A. Jones, T. -X. Liu

**Affiliations:** ^1^ Beneficial Insect Research Unit, Kika de la Garsa Subtropical Agricultural Research Center ARS-USDA, Weslaco, TX, USA.; ^2^ Current address: European Biological Control Laboratory ARS-USDA, France; ^3^ Texas Agricultural Experiment Station, Texas A&M University, Weslaco, TX USA

Studies were conducted to compare preference among *Bemisia tabaci* Gennadius biotype B instars for parasitization by *Encarsia pergandiella* Howard and *Eretmocerus mundus* Mercet under no choice and two and four instar choice experiments. In the no choice treatment, *E. mundus* was more successful in parasitizing the younger host instars, while *E. pergandiella* was more successful in parasitizing the older instars. When parasitoids females were presented six different pair combinations of a two instars at a time, parasitism by *E. mundus* was always greatest in the youngest of any of the two-instar combinations, except when first (29.2%) and second (31.0%) instars were presented together. Parasitism by *E. pergandiella* was always greater in the older instars, except significantly more third instars were parasitized when present with fourth instars. When equal numbers of all four host instars were provided simultaneously, the number of first, second, and third instars parasitized by *E. mundus* was not significantly different between each other (range: 10.3–16.4%), but four instars were parasitized at a significantly lower rate (2.1%). When *E. pergandiella* were presented with all four instars, parasitization was significantly highest in third instars (17.2%), and lowest in first (2.8%). Thus, each parasitoid species possesses a different range of instar preferences for reproduction. The present results provide additional important information toward explaining elements of interspecific interactions, and combined with ongoing research, should lead toward more efficient use of parasitoids to manage whiteflies.

# Sub article: Novel Technique for the Control of Insect Growth Regulator Resistant B-biotype Bemisia tabaci in Australia

**DOI:** 10.1673/031.008.0441

**Published:** 2008-01

**Authors:** Robin Gunning, Emma Cottage, Valerio Borzatta

**Affiliations:** ^1^ New South Wales Department of Primary Industries, Tamworth, Australia.; ^2^ Endura, SpA, Bologne, Italy

B-biotype *Bemisia tabaci* (the silverleaf whitefly), is a serious pest on cotton, grain, legume and horticultural crops in Australia. The silverleaf whitefly entered Australia resistant to most organophosphates, carbamates and pyrethroids and has since developed resistance to most other insecticides used for control, including insect growth regulators (IGRs), buprofezin and pyriproxyfen. There is cross-resistance between the two IGRs. IGR-resistance in the silverleaf whitefly is a major threat to the economic production of cotton in Australia and field control problems have occurred. IGR-resistance in Australian populations of B-biotype *B. tabaci* appears largely to be due to overproduced esterase isoenzymes, which apparently sequester the IGR insecticides. In this work, we investigated synergism with an esterase inhibitor, as a means to overcome IGR resistance in B-biotype *B. tabaci*. Esterase inhibitors such as organophosphates and piperonyl butoxide are commercially available, as they are used on cotton in Australia, in tank mixes with pyrethroids. Pyriproxyfen-resistant B-biotype *B. tabaci* nymphs were bioassayed with pyriproxyfen after exposure (by leaf dip) to a non-toxic dose of formulated piperonyl butoxide (PBO). Data indicated that PBO completely synergised pyriproxyfen and suppressed resistance. The use of synergists to control IGR-resistance could give effective control of B-biotype *B. tabaci* on cotton in Australia.

# Sub article: Performances of Three Types of Insect Screens as a Physical Barrier Against Bemisia tabaci and their Impact on TYLCV Incidence in Greenhouse Tomato in the Souss Valley of Morocco

**DOI:** 10.1673/031.008.0442

**Published:** 2008-01

**Authors:** A. Hanafi

**Affiliations:** IAV Hassan II, Complexe Horticole d'Agadir, Department of Plant Protection, IPP Unit, Agadir Morocco.

*Tomato yellow leaf curl virus* (TYLCV) was introduced to Morocco in 1998. Since then it has become the major challenge for tomato farmers in Morocco. The only way to seriously control the disease is by effectively controlling the vector. Although pesticides will remain an important tool for pest management in greenhouse crops, non chemical methods must be introduced to reduce damage to the environment and to delay build up of insecticide resistance. Screens have been found to be an efficient method for reducing the entry of pests into the greenhouse, and hence for reducing the number of insecticide applications targeting the insect pests and vectors. In this study we report on observations that were conducted in three greenhouses belonging to private farmers and which are equipped with three different types of insect screens 10^*^14, 10^*^20 and 10^*^22. We report on whitefly captures in sticky traps as well as TYLCV incidence in the tomato crop. The results indicate that the insect-net 10^*^22 gave the best exclusion of whiteflies followed by the insect net 10^*^20, whereas the screen 10^*^14 showed very poor performances. TYLCV incidence at the end of the crop cycle was estimated at 4%, 16% and 76% in the greenhouses equipped wit the screen 10^*^22, 10^*^20 and 10^*^14, respectively.

# Sub article: Looking for Bemisia tabaci biotype Q in Florida: Results of biotype sampling from 2005–2006.

**DOI:** 10.1673/031.008.0443

**Published:** 2008-01

**Authors:** Greg S. Hodges, Cindy L. McKenzie

**Affiliations:** ^1^ Florida Department of Agriculture and Consumer Services, Division of Plant Industry, Gainesville, Florida, USA.; ^2^ USDA, U.S. Horticultural Research Lab, Fort Pierce, Florida, USA

In March 2005, Arizona scientists identified the Q biotype of *Bemisia tabaci* from Poinsettia plants that originated from California. Soon after, Florida began forming a plan for surveying, identifying and dealing with the Q biotype. Efforts for surveying began immediately in March 2005 and have continued through present day. Pest alerts and educational efforts from the FDACS-DPI and the University of Florida have played a key role in getting involvement from growers, extension agents and agricultural inspectors. As of November 1, 2006 there have been a total of five Counties in Florida in which the Q biotype has been detected.

# Sub article: The Biotypes B and Q of Bemisia tabaci in Israel — Distribution, Resistance to Insecticides and Implications for Pest Management

**DOI:** 10.1673/031.008.0444

**Published:** 2008-01

**Authors:** R. Horowitz, Svetlana Kontsedalov, V. Khasdan, H. Breslauer, I. Ishaaya

**Affiliations:** ^1^ Dept. of Entomology, ARO, Gilat Research Center, Israel.; ^2^ Dept. of Entomology, ARO, The Volcani Center, Bet Dagan, Israel

Two biotypes of the whitefly, *Bemisia tabaci* (Gennadius) (Hemiptera: Aleyrodidae), have been documented from Israel. Biotype B was recognized in the early 1990s and biotype Q was first evidenced in 2000. In order to determine the biotype status of *B. tabaci* in Israel, various whitefly populations collected during 2000–2006 from field and greenhouse sites were examined with DNA markers. The markers were developed through cleaved amplified polymorphic regions (CAPS) analysis, based on primers complementary to the mitochondrial cytochrome oxidase I (mtCOI) gene sequences. The whitefly collections were part of a nationwide program to monitor resistance to several important whitefly control agents such as, pyriproxyfen, acetamiprid and diafenthiuron. Based on the collected samples, both the B and Q biotypes are present throughout Israel, and field populations may consist of a mixture of the two biotypes. Their distribution probably depends on crop type, climate, and the intensity of treatments with pesticides. Restrictions to interbreeding between the two biotypes of *B. tabaci* were found, and it may resolve differences in biochemical and physiological characters and symbiont composition of these biotypes. A link between *B. tabaci* biotypes and insecticide resistance was found under field and laboratory conditions. In various locations the Q biotype has reached moderate to high resistance levels to pyriproxyfen (Resistance ratio= 25 – 600-fold). No resistance to pyriproxyfen was detected in the B biotype. Accordingly, B biotype predominates on organic farms, while numerous insecticide applications have selected for Q biotype elsewhere.

# Sub article: Association of the ‘Distortion-Recovery Phenotype’ in ‘Anaheim’ Pepper Systemically Infected with the Non-Whitefly Transmissible Pepgmv-Distortion Strain (Di), with the BC1/Promoter Region

**DOI:** 10.1673/031.008.0445

**Published:** 2008-01

**Authors:** M. Idris, J. K. Brown

**Affiliations:** Department of Plant Sciences, The University of Arizona, Tucson, AZ, USA.

The whitefly non-transmissible strain of *Pepper golden mosaic virus* (PepGMV-Distortion (Di) (genus, *Begomovirus*) causes leaf distortion, mild mosaic, and ‘recovery’ symptoms in ‘Anaheim’ pepper *Capsicum annuum* L., and viral DNA is detectable by PCR in apical ‘recovered’ leaves that do not express disease symptoms. Two whitefly-transmissible mosaic strains, PepGMV-Mosaic (Mo) and PepGMV-Serano (Ser), which have both been isolated from pepper plants co-infected with PepGMV-Di, cause systemic yellow mosaic symptoms and plants do not ‘recover’ from infection. Laboratory assembled reassortants inoculated to pepper plants in all possible combinations of the PepGMV Di, Mo, and Ser DNA-A and DNA-B components were capable of causing systemic symptoms. Although the resultant phenotypes ranged from full blown, systemic ‘mosaic’ symptoms, to ‘veinal crumpling and foliar ‘distortion’ in the inoculated and in 1–2 subsequently developing leaves, and the absence of symptoms in apical leaves, or ‘recovery’ [*herein*, ‘distortion-recovery’], it was clear each symptom phenotype was linked to its respective, wild type viral DNA-B component. Nucleotide and amino acid sequence comparisons for the DNA-B component of the three PepGMV strains revealed considerable variation in the BC1 ORF (movement protein) and in the putative, upstream promoter region [*herein*, BC1+promoter], with Mo and Ser being highly similar. To investigate the role of this region of the PepGMV genome in symptom phenotype, the sequence was amplified using strain-specific PCR primers flanking the [ORF + promoter] and existing restriction sites *Nde* I and *Spe* I to exchange the Mo and Di [ORF + promoter] into the context of the respective DNA-B component background. The resultant DNA-B chimeric mutants, Mo-Di^/^ and Di-Mo^/^, were co-inoculated with the Mo or DI DNA-A component to ‘Anaheim’ pepper seedlings using biolistic inoculation. Controls consisted of pepper seedlings inoculated with wild type, infectious PepGMV-Mo and -Di clones, and mock-inoculated pepper plants (no DNA). Pepper seedlings inoculated with PepGMV Mo and Di exhibited characteristic wild type mosaic and ‘distortion-recovery’ symptoms, respectively, whereas, the Mo-Di^/^ and Di-Mo^/^ chimeras developed symptoms associated with the respective [BC1+ promoter] in the exchanged sequence. Results suggest that the ‘recovery’ phenotype may be associated with a defective BC1 promoter and/or the BC1 coding region, or both. If so, it would be anticipated that these functionally defective sequences might make possible host innate immune system countering of infection by PepGMV-Di, leading to the observed ‘recovery phenotype’, which effectively corresponds to a *latent resistance phenomenon*. This is in contrast to the development of a full blow systemic infections observed for PepGMV Mo and Ser, which both appear to contain fully functional [BC1+ promoter]-regions that the host apparently cannot overcome.

# Sub article: Monitoring Bemisia Resistance to Neonicotinoid Insecticides in Floriculture Crops

**DOI:** 10.1673/031.008.0446

**Published:** 2008-01

**Authors:** Terry Junek, Carlos E. Bográn, Patricia Pietrantonio

**Affiliations:** Department of Entomology, Texas A&M University, College Station, TX, USA.

*Bemisia* whiteflies continue to be key pests of floriculture crops, but the availability and efficacy of neonicotinoid-based control programs in the last 10 years have reduced their pest status in the industry. However, specific operational factors in greenhouse production increase the risk of resistance selection to neonicotinoid insecticides. First, the high value of ornamental plants allows growers to use insecticides at frequencies that would be un-economic in other cropping systems. Second, neonicotinoid insecticides can be applied as foliar sprays, root drenches or through irrigation systems. And third, their efficacy as both residual-systemic and as contact-foliar insecticides gives them great versatility but also renders them particularly prone to overuse. The observed patterns of resistance evolution to neonicotinoid insecticides in *Bemisia* populations affecting greenhouse crops in Europe and the recent introduction of several neonicotinoid insecticides for the floriculture industry indicate the need for resistance management strategies. The long-term objective of our work is to develop an insecticide resistance monitoring program for floricultural crops. The objectives of the present study are to initiate the process by focusing on a high risk pest-host plant complex, *Bemisia* on Poinsettias. Our specific objectives are to 1) obtain baseline susceptibility data for neonicotinoid insecticides in *Bemisia* whiteflies attacking floricultural crops; 2) develop and implement neonicotinoid insecticide resistance detection and monitoring programs for floricultural crops; and 3) develop and distribute neonicotinoid resistance management information for floricultural crops using existing extension education programs. Here we report results of preliminary bioassays to determine baseline susceptibility to imidacloprid in a *B. argentifolii* laboratory colony. A *Bemisia argentifolii* colony that has been maintained in the laboratory since 2000 without exposure to insecticides was used as a source of susceptible insects. Solutions containing from of 0 to 100 ppm imidacloprid (Marathon® II, OHP Inc. Mainland, PA) were used to drench cotton seedlings growing in soil-less media in 6 inch pots (soil systemic bioassay). Cotton was used as the host plant because it is easily grown from seed under laboratory conditions and for consistency with previously published methods. Seedlings with two true-leaves were cut at the base of the stem at 24, 48 and 72 hours after insecticide application to determine the effect of up-take time on lethal concentration (LC) values. Single treated seedlings were placed into micro-centrifuge tubes filled with distilled water to provide moisture and then placed inside 150 mm (diameter) × 25 mm (depth) Petri dishes (Falcon®, Becton Dickinson and Co. Lincoln Park, NJ). Twenty adults were introduced into each Petri dish and the dishes were sealed with parafilm (Parafilm®, American National Can™, Neenah, WI) to avoid whitefly escape. Each concentration was tested 4 to 8 times (one dish = one replicate) at each of the three up-take time treatments. Whitefly mortality was assessed 24 hours after exposure to treated seedlings. Probit analyses were conducted to determine LC50 and LC90 values. As expected, whitefly mortality increased linearly with imidacloprid concentration. LC50 values were 25.4, 1.2 and 0.2 ppm for bioassays with uptake times of 24, 48 and 72 hours, respectively. Similarly, LC90 values were 69.6, 26.8 and 5.4 ppm for bioassays with uptake times of 24, 48 and 72 hours, respectively. These values are lower than those previously reported from soil systemic bioassays but similar in magnitude to those reported from leaf-disk bioassays. Based on the slope of regression lines and the variability around LC50 value estimations in Probit analyses, an uptake time of 48 hours will be used in subsequent bioassays. The challenges and opportunities of resistance monitoring and development of insecticide resistance management strategies for floricultural crops will be discussed.

# Sub article: Within Plant Distribution of Bemisia tabaci and its Principal Parasitoids on Cassava Mosaic Disease (CMD) Resistant and Susceptible Varieties

**DOI:** 10.1673/031.008.0447

**Published:** 2008-01

**Authors:** Samuel Kyamanywa, Peter Asiimwe, Michael Otim, Dan Gerling, James P. Legg

**Affiliations:** ^1^ Department of Crop Science, Makerere University, Kampala, Uganda.; ^2^ International Institute of Tropical Agriculture - Uganda, Kampala, Uganda; ^3^ Zoology Department, Tel Aviv University, Ramat Aviv, Israel; ^4^ International Institute of Tropical Agriculture - Tanzania, Dar es Salaam, Tanzania; ^5^ Natural Resources Institute, University of Greenwich, Chatham Maritime, Kent, UK

Cassava mosaic disease CMD), caused by cassava mosaic geminiviruses and vectored by the whitefly, *Bemisia tabaci*, has been successfully managed using resistant cassava varieties. The CMD-resistant varieties, however, are heavily infested by *B. tabaci* causing direct injury resulting in significant reduction in tuberous root yield. Consequently there is a need to develop a CMD control strategy that also addresses the whitefly problem. Exploiting natural enemies is one of the strategies that is being explored. Knowledge of the distribution and incidence of the existing natural enemy fauna is a prerequisite for developing effective biological control. A study was therefore carried out to establish the distribution of *B. tabaci* parasitoids on cassava plants in order to determine the stage of the plant at which parasitoids start colonizing *B. tabaci* and to develop a sampling protocol for the parasitoids. The study was conducted using the CMD-resistant cassava variety, Nase 4 and the CMD-susceptible Ebwanateraka. There were significant differences in the distribution of both the whitefly and the parasitoids between different parts of the plant. For both varieties, over 65% of the whitefly nymphs were found on leaves 10 to 17 from the shoot apex of the plant. The density of *B. tabaci* nymphs decreased with age of the plant with over 60% of the nymphs occurring within 17 weeks after planting of cassava. Parasitism was highest on lower leaves (16 – 24). The level of parasitism varied with the maturity of the plant. Parasitism was least in young plants (11 weeks), highest in 19 week-old plants and thereafter dropped in plants more than 21 weeks old. Peak parasitism at a given growth stage depended on leaf position. For the susceptible variety, peak parasitism for young (11–13 week-old) cassava plants occurred on leaf number 14, while for older plants (17–19 week-old), parasitism peaked on leaves 18 and 21 respectively. The implications of the present findings for designing sampling procedures for both the whitefly and its parasitoids, and for studying the impact of the parasitoids on whitefly populations are discussed in this paper

# Sub article: Symptoms and Complete Nucleotide Sequence Analysis of Sweet Potato Leaf Curl Virus Transmitted by Bemisia tabaci

**DOI:** 10.1673/031.008.0448

**Published:** 2008-01

**Authors:** Hae-Ryun Kwak, Mi-Nam Jung, Mi-Kyeong Kim, Minho Lee, Park Jin-Woo, Lee Su-Heon, Choi Hong-Soo

**Affiliations:** ^1^ Department of Agricultural Biology, NIAST, RDA, Suwon, Republic of Korea.; ^2^ Mokpo Experiment Station, NICS, RDA, Mokpo, Republic of Korea

*Sweet potato leaf curl virus* (SPLCV) is a member of the genus *Begomovirus* of the family *Geminiviridae,* and is transmitted by *Bemisia tabaci* in a persistent manner. A sweet potato disease in Korea causing leaf curl symptom was identified as SPLCV based on biological, cytopathological, and molecular properties. The isolate could be detected in and transmitted by *Bemisia tabaci* Q biotype which recently appeared in Korea, but not by mechanical inoculation. Ribbon-like and rectangular inclusions were shown in the nuclei of infected plant cells. The complete nucleotide sequence was determined using 5 overlapping PCR clones from nine isolates. These isolates were similar in genome organization to SPLCV-US including six open reading frames (ORFs). The nine isolates could be divided into two groups based on genomic sequence identity. Their identity indices of intragroup and intergroups showed 100% and 97%, respectively. The relationships between these isolates and SPLCV-US were determined using phylogeny of the derived AV1, AV2, AC1, AC2, AC3, and AC4 amino acid sequences. The results indicate that all of the isolates might have evolved from a common ancestor possibly from the old world.

# Sub article: Tomato Plants and Whitefly Interactions: Can It Be a Love Story?

**DOI:** 10.1673/031.008.0449

**Published:** 2008-01

**Authors:** M. Lapidot, D. Levy

**Affiliations:** ^1^ Institute of Plant Sciences, Volcani Center, ARO, Bet Dagan, Israel.; ^2^ Hazera Genetics, Mivhor, Israel

Apart from virus transmission, whiteflies cause extensive direct damage to crops through excessive sap removal, excretion of honeydew that promotes the growth of sooty mold fungi, and by inducing systemic developmental disorders. In tomato, whiteflies have been shown to induce irregular ripening (Schuster *et al*. 1990. *HortScience* 25, 1618–1620). Is this always the case? In a study of the levels of resistance to *Tomato yellow leaf curl virus* (TYLCV) among different tomato genotypes, plant performance following inoculation was compared between plants exposed to non-viruliferous whiteflies and plants not exposed to whiteflies. Total yield and yield components were measured. As expected, all the TYLCV-infected plants expressed disease symptoms, and suffered from growth inhibition and a yield reduction due to the devastating effect of the virus. The viral induced damage was negatively correlated with the level of resistance displayed by the test plant. Therefore, the higher the TYLCV resistance, the less the yield reduction. To our surprise some of the genotypes exposed to non-viruliferous whiteflies did not show any whitefly-induced damage. Moreover, the yield components of some of the whitefly-treated tomato cultivars were greater than that of the control untreated plants.

# Sub article: Occurrence of Bemisia tabaci B-and Q-Biotypes in Korea

**DOI:** 10.1673/031.008.0450

**Published:** 2008-01

**Authors:** Minho Lee, Uhm Ki-Baik, Lee Sunyoung, Park Heeyong, Choi Hong-Soo, Park Jin-Woo

**Affiliations:** Department of Agricultural Biology, NIAST, RDA, Suwon, Republic of Korea.

The occurrence of *Bemisia tabaci* in greenhouses has been monitored in Korea since the first report about the introduction of Q biotype in 2005. The *Bemisia tabaci* B biotype has occurred only in rose greenhouses of a middle region (Jincheon county) in Korea since 1998. The host plant of the Q biotype, however, was sweet pepper, hot pepper, tomato, eggplant, and cucumber in the southwest (total 24 cities or counties) of Korea. The biotypes and genetic differentiation of the whiteflies collected in each regions were analyzed by mitochondrial 16S DNA and CO1 gene sequences. The Q biotype spread rapidly to other regions from invaded southern region in Korea, and various host plants during the year. *Tomato yellow leaf curl virus* (TYLCV), however, has not yet been found in tomato or sweet pepper greenhouses in Korea.

# Sub article: Life History of Delphastus Catalinae: A Predator of Bemisia tabaci

**DOI:** 10.1673/031.008.0451

**Published:** 2008-01

**Authors:** Jesusa Crisostomo Legaspi, Alvin M. Simmons, Benjamin C. Legaspi

**Affiliations:** ^1^ USDA, ARS, CMAVE-FAMU-CBC, Tallahassee, FL, USA.; ^2^ U.S. Vegetable Laboratory, USDA-ARS, Charleston, SC, USA; ^3^ FL PSC, FL, USA

Immature development and reproductive life history of *Delphastus catalinae* (Coleoptera: Coccinellidae) feeding on *Bemisia tabaci* (Homoptera: Aleyrodidae) immatures was studied at three constant temperatures: 22, 26 and 30 °C. Development rates and lower temperature threshold temperatures (*T _0_* ) were estimated at 9 and 9.9 °C, for males and females, respectively. Thermal units required for immature development was about 300 degree-days in both sexes. As temperature increased from 22 to 30 °C, immature development time from eggs to pupae declined from 24 to 15 days, and adult longevity ranged from 138 and 77 days. The intrinsic rate of increase *r _m_* , increased from 0.048 to 0.082 and doubling time *DT* decreased from 14.44 to 8.45 days as temperature increased from 22 to 26 °C. Results indicated that *D. catalinae* was found to perform best from 22 and 26 °C; while 30 °C was detrimental to immature development and adult reproduction. In a separate experiment, we studied the effect of plant species and insect stages on feeding by *D. catalinae* on *B. tabaci.* To study the influence of plant species, immature whitefly prey were presented simultaneously to starved predator adults on leaf cuttings of five different plant species: cotton (*Gossypium hirsutum*), tomato (*Lycopersicon esculentum*), *Hibiscus* (*Hibiscus rosasinensis*), cowpea (*Vigna unguiculata*) and collard (*Brassica oleracea*). Percentage predation over 24 h was significantly highest on cotton, followed in rank order by collards, cowpea and tomato, and lowest on *Hibiscus.* Different predation rates may have been caused by differential response to volatile secondary compounds released by the leaf cuttings. Host stage preference was studied by presenting individual adult predators with equal numbers of prey (200 per replicate) in three aggregate life stages: eggs, small nymphs (1^st^ to 3^rd^ instars) and large nymphs (4^th^ to pupae). Significantly higher numbers of eggs were consumed in a 24-h predation period, compared with small or large nymphs. These findings suggest that among the plant species tested, *D. catalinae* may be most effective on early-season cotton or immediately after whitefly infestation when eggs are predominant.

# Sub article: Changes in the Biology of Bemisia tabaci on Cassava in Africa and Their Impact on Virus Disease Pandemics

**DOI:** 10.1673/031.008.0452

**Published:** 2008-01

**Authors:** James P. Legg, Peter Asiimwe, Peter Sseruwagi, Judith K. Brown

**Affiliations:** ^1^ International Institute of Tropical Agriculture-Tanzania, Dar es Salaam, Tanzania.; ^2^ Natural Resources Institute, Chatham Maritime, Kent, UK; ^3^ International Institute of Tropical Agriculture-Uganda, Kampala, Uganda; ^4^ Kawanda Agricultural Research Institute, Kampala, Uganda; ^5^ University of Arizona, Tucson, Arizona, USA

*Bemisia tabaci* has been recognized as the vector of cassava mosaic geminiviruses (CMG) for many years. More recently, it has also been shown to transmit cassava brown streak virus (CBSV), which causes cassava brown streak disease (CBSD). Super-abundant populations of *B. tabaci* and the recombinant CMG, East African cassava mosaic virus-Uganda, have been associated with the expansion through nine countries of East and Central Africa of the African pandemic of severe cassava mosaic disease (CMD). The pandemic currently affects over 100 million people in an area greater than 2.6 million square kilometers and continues to expand, primarily southwards and westwards. Experiments to investigate the biology and population genetics of super-abundant *B. tabaci* have revealed a number of novel features of this pest and its relationship with its cassava host. Yield loss studies aimed at quantifying physical damage have shown that losses solely attributable to physical feeding are as high as 45%. Local cultivars are highly susceptible to CMD but relatively unaffected by whitefly feeding damage and the associated sooty mould. Improved varieties currently being promoted in view of their resistance to CMD have variable levels of yield loss resulting from whitefly physical damage (12.5–44.6%), but host consistently higher populations of *B. tabaci* and therefore have more feeding and sooty mould damage. Pesticide regimes required to protect cassava crops from the effects of whitefly physical damage consisted of two soil-drench applications of imidacloprid (at one and four months after planting), and weekly foliar applications of cypermethrin. Comparisons of whitefly abundance in these chemically controlled plots (2002–4) with unprotected plots of similar varieties prior to the passage of the pandemic (1992–4) revealed a similar average value over the first eight months after planting. Efforts to determine the cause of the change in *Bemisia* populations on cassava have focused both on interactions with virus-infected cassava host plants and the genetic characteristics of *B. tabaci* populations. Preliminary evidence based on MtCO1 sequence comparisons suggested the occurrence of pandemic-associated genotype clusters. More recent work using nuclear markers seems to confirm the hypothesis that genetically distinct ‘invasive’ populations are linked with the super-abundance phenotype and concomitant role as a key determinant of pandemic spread. Recent increases in CBSD incidence in Uganda and unconfirmed reports of the disease in Democratic Republic of Congo and western Kenya raise the worrying possibility that super-abundant *B. tabaci* are now driving a dual pandemic.

# Sub article: Oviposition, Development, and Survivorship of Eretmocerus melanoscutus (Hymenoptera: Aphelinidae) Parasitizing Nymphs of Bemisia tabaci (Homoptera: Aleyrodidae)

**DOI:** 10.1673/031.008.0453

**Published:** 2008-01

**Authors:** Tong-Xian Liu

**Affiliations:** Vegetable IPM Laboratory, Texas Agricultural Experiment Station, Texas A&M University System, Weslaco, TX, USA.

*Eretmocerus melanoscutus* Zolnerowich & Rose was originally imported from Thailand, and has been released in southern Texas for biological control of *Bemisia tabaci* (Gennadius) biotype ‘B’. It was found parasitizing *B. tabaci* on various vegetables in the greenhouses at the Texas Agricultural Experiment Station, Texas A&M University System at Weslaco, Texas in 2002. Studies were conducted to determine biological parameters of *E. melanoscutus,* and evaluate its potential as a biological control agent of *B. tabaci* under laboratory conditions (26.7 ± 2°C, 50–75% RH and a photoperiod of L:D = 14:10 h). Developmental time of *E. melanoscutus* was 5.3, 7.0, and 4.5 d for eggs, larvae, and pupae, respectively, with an average of 16.8 d from oviposition to adult emergence. Adult longevities of *E. melanoscutus* averaged 9.4 d for females and 13.2 d for males. Females normally oviposit on the first day after emergence. Each female laid an average of 122.7 eggs during her lifespan or 12.5 eggs per day. Preimaginal survivorship was estimated at 93.4%. The intrinsic rate of natural population increase (*r*), net reproductive rates (*R _0_*), generation time (*T*), and doubling time (*DT*), and the finite rate of increase (λ) of *E. melanoscutus* were estimated by life table analysis at 0.2175/day, 61.42 offspring, 19.0 d, 3.2 d, and 1.2429/day, respectively. The results suggest that *E. melanoscutus* has a great potential as a biological control agent of *B. tabaci*.

# Sub article: Impact of Insecticide Residue on Bemisia tabaci (B-biotype)

**DOI:** 10.1673/031.008.0454

**Published:** 2008-01

**Authors:** Scott W. Ludwig, Cindy McKenzie

**Affiliations:** ^1^ Texas Cooperative Extension, Overton, TX, USA.; ^2^ U. S. Horticultural Research Laboratory, Fort Pierce, FL, USA

In 2005, the Q-biotype of *Bemisia tabaci* was identified in the United States. This finding and increased problems with management of the B-biotype of *Bemisia tabaci* have resulted in a national effort to develop a comprehensive management plan for whiteflies on ornamental crops. The objective of the following study is to evaluate the activity of insecticide residue against silverleaf whiteflies to aid in the development of a whitefly resistance management program. Three studies have been conducted evaluating pesticide residue on poinsettia plants (Freedom Red) against *B. tabaci* biotype B. In the first study we evaluated pesticide residue at three time intervals after a single foliar application to poinsettia plants. The following treatments were evaluated at labeled rates: Judo (spiromesifen), Avid 0.15EC (abamectin), Sanmite 75WP (Pyridaben), Enstar II (S-kinoprene), Endeavor 50WG (pymetrozine), Distance IGR (pyriproxyfen), and an untreated control (see Table 1 for rates). Clip cages were placed onto leaves at 7 hrs, 8 days, and 14 days after application. These leaves were fully expanded when the plants were treated. Ten adult whiteflies of mixed sex and age were then placed into each cage. After 48 hours the number of eggs and dead whiteflies were recorded. Eggs were allowed to hatch, nymphs develop, and adults emerge. The number of emerged adults was then recorded. Similar procedures were followed in the second and third experiments, except the trials conducted for longer time periods. In the second trial the following neonicotinoids were evaluated as drenches: imidacloprid, clothianidin, dinotefuran and thiamethoxam. In the third trial the following neonicotinoids were evaluated as foliar sprays: imidacloprid, clothianidin, dinotefuran, thiamethoxam and acetamiprid. Results from all three trials will be presented at the meeting. Data were transformed using an arcsine transformation prior to analysis. Data were analyzed with ANOVA and means separation was accomplished by using the least significant difference test (LSD) at the *P*<0.05 level. All data are presented as original means. No insecticide provided greater that 70% direct adult mortality (Table 1). Distance provided 100% control of the resulting generation on all sample dates (Table 2). Judo provided greater than 80% control of the resulting generation on all sample dates. Avid provided greater than 90% control for the first two sample periods. The other insecticides have varying levels of residue activity. These results indicate that none of the products evaluated will give effective control of adults once the insecticides have dried. However, Distance, Judo and Avid provided excellent immature whitefly control for 10 days while Distance and Judo provided an additional 6 days of excellent and good control, respectively. Sanmite managed to kill over 60% of the nymphs during the periods evaluated. This trial will be repeated and additional trials are planned to evaluate the residual activity of other insecticides used to manage whiteflies. These results will enable grower and extension personnel to better understand the residual activity of insecticides. This will, in turn, result in better insecticide rotation programs for the management of whiteflies.

# Sub article: Development, Longevity, Fecundity, and Survivorship of Bemisia tabaci (Gennadius) Biotype ‘B’ on Six Cotton Cultivars

**DOI:** 10.1673/031.008.0455

**Published:** 2008-01

**Authors:** Chen Luo, Gao Qing-Gang, Mo Tie-Lu, Fan Zhang, Zhang Zhi-Li

**Affiliations:** ^1^ Institute of Plant and Environment Protection, Beijing Academy of Agriculture and Forestry Sciences, Beijing, China.; ^2^ Department of Entomology, College of Plant Protection, Shandong Agricultural University, Taian, China

We studied the development, longevity, fecundity, and survivorship of *Bemisia tabaci* biotype ‘B’ on six cotton cultivars: GK-12 (Bt), AST-104, CCRI 19, Liao-Yang (hirsute), ZGK-9708 (Bt+CpTI), and Liao 7238 at 27 ± 1°C, 60% RH and a photoperiod of 14:10 (L:D) h in the laboratory. Our results show that the developmental time, survivorship, and fecundity of *B. tabaci* were significantly different on the six cotton cultivars except for the adult longevity that was not significantly different among the cotton cultivars. The developmental times of *B. tabaci* from egg to adult were 22.3, 21.9, 24.2, 21.6, 22.5 and 20.8 d on GK-12 (Bt), AST-104, CCRI 19, Liao-Yang (hirsute), ZGK-9708 (Bt+CpTI) and Liao 7238, respectively. The total survivorship from egg to adult was 39.3, 37.0, 29.3, 58.3, 38.8, and 25.0%, respectively. Fecundity varied from 138.1 eggs per female on CCRI 19 to 80.8 eggs per female on GK-12. The intrinsic rate of natural increase (*r _m_* ) of *B. tabaci* on Liao-Yang (hirsute) was the highest (0.1121), where that on Liao 7238 was the lowest (0.0844). The development and reproduction of *B. tabaci* on the two Bt-transgenic cotton cultivars (GK-12 and ZGK-9708) were not significantly different from those on the other four non-Bt transgenic cottons.

# Sub article: Fitness of Encarsia sophia (Hymenoptera: Aphelinidae) Parasitizing Trialeurodes vaporariorum and Bemisia tabaci (Homoptera: Aleyrodidae)

**DOI:** 10.1673/031.008.0456

**Published:** 2008-01

**Authors:** Chen Luo, Tong-Xian Liu

**Affiliations:** ^1^ Vegetable IPM Laboratory, Texas A&M Agricultural Experiment Station, Weslaco, TX, USA.; ^2^ Institute of Plant and Environment Protection, Beijing Academy of Agriculture and Forestry Sciences, Beijing, China

Fitness of *Encarsia sophia* (Girault & Dodd) parasitizing *Trialeurodes vaporariorum* (Westwood) and *Bemisia tabaci* (Gennadius) biotype ‘B’ was investigated on green bean under laboratory conditions at 27 ± 2°C, 55 ± 5% RH, and a photoperiod of 14:10 (L: D) h. Development and survivorship of immatures and adults, body size, longevity and fecundity of adult females were determined. The results indicate that the fitness of *E. sophia* that parasitized on two different host species differed significantly. Almost all biological parameters of *E. sophia* parasitizing *T. Vaporariorum* were superior to those parasitizing *B. tabaci*.

# Sub article: Influences of Bt Cotton on Population Dynamics of Bemisia tabaci

**DOI:** 10.1673/031.008.0457

**Published:** 2008-01

**Authors:** Raj S. Mann

**Affiliations:** University of Florida, Gulf Coast Research and Education, Wimauma, Florida, USA.

*Bemisia tabaci* (Gennadius) is a major sucking pest of cotton in India and other cotton growing areas of the world. The Indian government permitted commercial cultivation of genetically modified Bt (*Bacillus thuringiensis*) cotton in Northern India in early April 2005. One of the most prominent questions concerning Bt cotton is its effect on non-target organisms. The field experiments were conducted at Punjab Agricultural University, Ludhiana, to investigate the effects of Bt cotton on population dynamics of whitefly and predators in plots of Bt cotton and conventional cotton that received no insecticide applications and plots of conventional cotton in which insecticides were used regularly for control of *bollworms*. The results indicated that mean population densities of whitefly did not differ significantly in Bt cotton plots and conventional cotton plants. However, populations in Bt cotton plots showed fluctuations and reached peaks at different times than in conventional cotton. The population of predators (*Chrysoperla*, coccinelids, predatory bugs and spiders) was significantly lower in insecticide treated plots of conventional cotton than untreated plots of conventional and Bt cotton.

# Sub article: Current Situation of Bemisia tabaci Vegetable Crops in Portugal

**DOI:** 10.1673/031.008.0458

**Published:** 2008-01

**Authors:** Célia Mateus, F. Amaro, D. Louro, A. Mexia

**Affiliations:** Plant Protection Department. Estação Agronómica Nacional, Oeiras, Portugal.

In Portugal, *Bemisia tabaci* was first recorded in 1992 in vegetable crops and since 1995, it has been an important pest in Algarve, southern Portugal. In this region, *B. tabaci* is present year-round in greenhouse crops, with high infestation levels during summer. It is a serious problem to several vegetable crops, due partly to direct damage, but mainly to acquisition and transmission of plant viruses. The most severely affected crops are greenhouse tomato and cucurbits. IPM is considered the best way to deal with the sustainable management of this problem. Nevertheless, the presence of the viruses complicates the implementation of IPM programs and the integration and synergy of multiple control tactics has to be considered. The work presented here gives an overview of the results obtained through research activities carried out in this country regarding *B. tabaci*: geographical distribution, bioecology, biotype identification, virus transmission, monitoring, risk assessment and control methods.

# Sub article: Monitoring the U. S ornamental industries B'S and Q's

**DOI:** 10.1673/031.008.0459

**Published:** 2008-01

**Authors:** C. L. McKenzie, Jim Bethke, Laura Boykin, Frank Byrne, Joe Chamberlin, Dan Gilrein, Scott Ludwig, Ron Oetting, Lance Osborne, Lin Schmale, Robert G. Shatters

**Affiliations:** ^1^ Subtropical Insect Research Unit, U. S. Horticultural Research Laboratory, USDA-ARS, Fort Pierce, FL, USA.; ^2^ Department of Entomology, University of California, Riverside, CA, USA; ^3^ Valent USA Corporation, Snellville, GA, USA; ^4^ Cornell University Cooperative Extension of Suffolk County, Long Island Horticultural Research and Extension Center, Riverhead, New York, USA; ^5^ Texas Cooperative Extension, Texas A&M University, Overton, TX, USA; ^6^ College of Agriculture and Environmental Sciences, University of Georgia, Griffin, GA, USA; ^7^ Mid-Florida Research and Extension Center, IFAS, University of Florida, Apopka, FL, USA; ^8^ Society of American Florists, Alexandria, VA, USA

Biotype “Q” of *Bemisia tabaci* was first detected in the United States on poinsettias from a southwest retail outlet in Arizona during December 2004. During the past 20 months, biotype Q has been detected in 22 states and appears to be spreading. Although indistinguishable in appearance from silverleaf whitefly (biotype B), these insects are much less susceptible to insect growth regulators and many neonicotinoid insecticides. Our primary objective was to monitor populations of *B. tabaci* from grower sites throughout the United States, in an effort to document the distribution of the Q-type within the ornamental industry. The ornamental grower response to the Q biotype issue has been tremendous, and many have sent in samples for testing. Without their contribution, the extent of the Q problem in the United States would not be known. Molecular techniques used to distinguish whitefly biotypes included esterase assays, analysis of mitochondrial COI DNA sequence, and microsatellite technology. Q biotype *Bemisia* can be distinguished from B biotype insects based on electrophoresis of the banding patterns of their esterases and this method was used to routinely confirm biotype status results utilizing cytochrome oxidase I (COI) sequence and microsatellite data analysis. For COI and microsatellite biotype determination, DNA was extracted from single whitefly using the Cartagen Genomic DNA extraction kits following the manufacturer's protocols (Cartagen Molecular Systems, Inc. Seattle, WA). Mitochondrial COI sequence analysis was performed, by first PCR amplification of an approximately 800 bp COI DNA fragment and then sequencing the PCR amplified DNA. Sequencing reactions were analyzed on an Applied Biosystems 3730XL sequence analyzer and the resulting sequence was compared and edited using Sequencher software (Gene Codes, Ann Arbor, MI). Biotype determination was inferred from neighbor joining methods of phylogenetic analysis of the COI sequences using CLUSTALW alignments of each sequence type. Two microsatellite primers developed by De Barro et al. (2005), BEM6 -(CA)8IMP and BEM23 -(GAA)31IMP, were found to be diagnostic for B and Q biotypes and were used to determine biotype status in conjunction with analysis of mitochondrial COI DNA sequence. Results of the monitoring effort revealed 100 % concurrence in biotype determination using COI sequence and microsatellite markers. More than one positive Q sample was reported from some states and currently 22 states have reported biotype Q. Interestingly, in over 1,000 B-biotype individuals collected from throughout the continental U.S., there was no variation in the COI sequence. However, in the Q biotype COI data from approximately 750 individuals, there were 12 polymorphic sites that created 4 different COI haplotypes. The host plant from which growers were sampling whiteflies were varied, but were predominantly poinsettias. Other host plants included gerbera daisies, hibiscus and verbena. States that were identified as positive were not overrun with biotype Q and all populations were managed. With knowledge of the whereabouts of this biotype, more effective action can be taken to manage its spread, thereby preventing considerable economic losses to the ornamental industry.

# Sub article: Determining the Role of Ethylene Biosynthesis in the Development of Tomato Irregular Ripening Disorder Using Microarray Technology and rt-Real Time PCR

**DOI:** 10.1673/031.008.0460

**Published:** 2008-01

**Authors:** C. L. McKenzie, X. H. Sinisterra, J. P. Albano, C. A. Powell, Scot E. Dowd, R. G. Shatters

**Affiliations:** ^1^ Subtropical Insect Research Unit, U. S. Horticultural Research Laboratory, USDA-ARS, Fort Pierce, FL, USA.; ^2^ Indian River Research and Education Center, IFAS, University of Florida, Fort Pierce, FL, USA; ^3^ Livestock Issues Research Unit, USDA-ARS, Lubbock, TX, USA

Whiteflies, *Bemisia tabaci* biotype B, are associated with a plant physiological disorder termed tomato irregular ripening. Symptoms of tomato irregular ripening disorder do not appear on tomato foliage where whitefly feed, but appear as an uneven fruit color development during ripening in tomato. This disorder is characterized externally by incomplete reddening of the fruit exhibited by longitudinal white or yellow streaks that typically start as a star at the blossom end of the fruit which can become more pronounced with the points radiating up the fruit. Color often develops along locule walls with intermediate areas remaining green or yellow, producing a star-burst appearance. Internally, fruit exhibit white or yellow tissues. The mechanism(s) of symptom development and the physiological bases of this disorder are not completely understood although altered gibberellin metabolism and suppression of ethylene biosynthesis in tomato fruit as a result of whitefly feeding have been indicated. In order to determine the underlying causes of tomato irregular ripening disorder associated with whitefly feeding, microarray hybridization analysis followed by reverse transcription (rt) real time PCR validation were used to determine gene regulation in young and old leaf tissue, stems, flowers, roots, and fruits over time. We found that tomato plants infested with whiteflies compared to non-infected had higher N levels (18.5%); were taller, wider, produced more leaves, flowers (2,998 vs. 2,443) & fruit (871 vs. 828), however significantly fewer fruit ever became ripe (48 vs. 272). Based on sequence similarity analysis we determined that selected genes were likely to be involved with ethylene biosynthesis production. Microarray analysis results of the first harvest (21 days after whitefly infestation) indicated a total of 68 genes involved in ethylene biosynthesis and were up or down-regulated in response to whitefly feeding after 21 days exposure. Seven genes involved in ethylene biosynthesis responded to whitefly feeding in new leaf tissue (3 up-regulated, 4 down regulated); 18 genes in old leaf tissue (10 up-regulated, 8 down-regulated); 4 genes in stem tissue (all up-regulated); 13 genes in root tissue (all up-regulated); 15 genes in flower tissue (10 up-regulated, 5 down-regulated); and 11 genes responded in fruit tissue (8 up-regulated, 3 down-regulated). After 3 weeks of whitefly infestation, the greatest changes were in old leaf tissue (4.78 fold) and fruit (-8.58 fold). Genes with the greatest changes were chosen for validation using rt-Real Time PCR and changes in gene regulation were monitored over time (21, 45, and 65 days after whitefly infestation). Results will be presented and ethylene's role in the development of tomato irregular ripening disorder will be discussed.

# Sub article: Influence of Weeds on the Whitefly-Virus-Parasitoid Complex in Veracruz, Mexico

**DOI:** 10.1673/031.008.0461

**Published:** 2008-01

**Authors:** Salomón Medina, L. D. Ortega-Arenas, H. González-Hernández, J. A. Villanueva

**Affiliations:** ^1^ Programa en Entomología, Campus Montecillo, Colegio de Postgraduados, Montecillo Estado de México.; ^2^ Programa en Agroecosistemas Tropicales, Campus Veracruz, Colegio de Postgraduados, Veracruz, México

The influence of the planned presence of weeds on the whitefly *Bemisia tabaci*-virus-parasitoids complex in the central coastal region of Veracruz, Mexico was studied. A randomized block design with three trfree growth of weeds; and c) tomato without weeds. The population density of *B. tabaci* adults and nymphs per leaflet, the viral incidence, the percentage parasitism and the crop yield, was recorded weekly. Weeds host for *B. tabaci* were identified and the average parasitism of the pest was determined. The presence of weeds favored the presence of parasitoids, reduced the population density of *B. tabaci* and the incidence of virus. Even when the presence of weeds delayed the incidence of virus, their presence also decreased the crop yield because of competition for resources. The selective presence of weeds should be planned in time and space, in order to protect the crop in its stage of most susceptibility to the viruses transmitted by *B. tabaci*.

# Sub article: Identification of Bemisia argentifolii Resistant Loci from Solanum habrochaites Accession LA1777

**DOI:** 10.1673/031.008.0462

**Published:** 2008-01

**Authors:** Aliya Momotaz, Jay W. Scott, David J. Schuster

**Affiliations:** Gulf Coast Research and Education Center, IFAS, University of Florida, Wimauma, FL, USA

Tomato, Solanum lycopersicon L. (formerly Lycopersicon esculentum Mill.) is a widely distributed and economically important crop throughout the world. Insect pests may limit successful crop production. The silverleaf whitefly (SLWF) *Bemisia argentifolii* Bellows & Perring [also known as biotype B of the sweetpotato whitefly, *B. tabaci* (Genn.)] is a particularly destructive pest of tomato in many tropical and subtropical regions. Damage is caused directly by its feeding habit and indirectly by the transmission of more than 50 different begomoviruses. Often SLWF is difficult to control solely with insecticides because of its ability to develop resistance to insecticides, thus causing serious problems for crop producers. Resistant cultivars would be an economical and environmentally sound way of managing *B. argentifolii* in tomato production. The wild accession LA1777 of *S. habrochaites* (formerly known as *Lycopersicon hirsutum*) was reported to be highly resistant to whiteflies. The objective of this study was to identify the gene(s) responsible for resistance to SLWF. In our earlier work no resistance was detected in any recombinant inbred lines (RIL) derived from tomato crossed with LA1777. An interspecific F2 population that was developed in our laboratory was assayed for resistance based on whitefly oviposition in no choice evaluations and on type IV trichome counts. From the F2 population 11 resistant and 10 susceptible plants were selected and were used to locate resistance genes by testing them with molecular markers. Over 400 molecular markers that span the tomato genome at about 10cM intervals have been tested. So far, markers in 5 regions on 4 different chromosomes appear to be associated with resistance, while markers in 4 other regions are less clearly associated with resistance. Crosses have been made between RILs to combine the putative resistance regions. Plants with combined target regions will be assayed for resistance to verify their role in SLWF resistance.

# Sub article: Progress in Positional Cloning of CMD2 the Gene that Confers High Level of Resistance to the Cassava Mosaic Disease (CMD)

**DOI:** 10.1673/031.008.0463

**Published:** 2008-01

**Authors:** Isabel Moreno, Jeffrey Tomkins, Emmanuel Okogbenin, Martin Fregene

**Affiliations:** ^1^ Centro Internacional de Agriculture Tropical (CIAT), Cali, Colombia.; ^2^ Clemson University Genomics Institute (CUGI), Clemson, South Carolina, USA; ^3^ International Institute for Tropical Agriculture (IITA), Ibadan, Nigeria, Africa

We describe the development of a chromosome walking experiment using different strategies toward cloning a Cassava Mosaic Disease (CMD) resistance gene (*CMD2*). The first part of our project was the fine mapping of a population made up of 1690 individuals from a cross between TME-3, the source of *CMD2* and the improved variety TMS30572. Two SSR markers previously reported NS158 and SSRY 28 were evaluated in the fine-mapping population and 112 recombinant individuals were identified. Two bulks from resistance and susceptible individuals were constructed and evaluated with several molecular marker systems including AFLP (STMPs, AFLP-NBS), ISTRs, RAPDs and SSR in a modified Bulk Segregant Analysis (BSA). Two polymorphic RAPD markers were identified in the gene region. The new linkage map with all markers reported, showed the resistance gene (CMD2) flanked by RME-1 and NS158 markers. A bacterial artificial chromosome (BAC) library with more than 10X coverage for the cassava genome was constructed from cassava variety TME3 for contig mapping. The BAC library was screened with RME-1 and NS158 markers by PCR amplification of BAC pools and 14 RME-1 positives clones and 2 NS158 clones were identified. Restriction enzyme fingerprinting with Hind III was employed to construct two BAC contigs around those markers using the FPC program. Ends of each contig were sequenced to design allele specific and SSCP-SNP primers to map the BAC ends into the CMD2 region. Additionally, new set the molecular markers are currently designed using the BLAST homology between the molecular markers associated with this characteristic and the castor bean genome sequenced. Successive screening with the BAC end markers and new COS markers in the BAC library will be carried out until a contig that contains the CMD2 gene is obtained.

# Sub article: The involvement of glutathione s-tranferases from Bemisia tabaci (Hemiptera: Aleyrodidae) in plant-insect interactions

**DOI:** 10.1673/031.008.0464

**Published:** 2008-01

**Authors:** Shai Morin, Fishel Alon, Michal Alon

**Affiliations:** Department of Entomology, the Hebrew University of Jerusalem, Faculty of Agricultural, Food and Environmental Quality Sciences, Rehovot, Israel.

Polyophagous insects like *Bemisia tabaci* are capable of feeding on a wide range of plant families. As a result, they have to cope with a tremendous diversity of plant defense secondary compounds. In many cases, resistance to these compounds is achieved by the activity of metabolic enzyme families like glutathione s-tranferases (GSTs), which are capable of detoxifying (among other substrates) glucosinolates (GLS), produced mainly by plant species belonging to Brassicaceae. Here, we compared the expression level of three GST genes previously isolated from *B. tabaci*. Throughout our experiments, *B. tabaci* individuals were subjected to two Brassicaceae hosts (mustard and cabbage) and one non-Brassicaceae host (cotton). When *B. tabaci* adults were transferred from cotton to mustard or cabbage, the expression level of all three GST genes increased (1.7–2.74 fold). When adults were returned back to cotton, all three genes showed reduced expression level but only one gene (designated GST2) showed a significant decrease (1.84 fold in transition from mustard to cotton). In order to examine the possibility the *B. tabaci* individuals are capable of switching on and off their GST genes is response to their plant host, the expression level of the three genes was examined for nine generations. In each generation, adults were allowed to choose between three host plants: cotton, mustard or cabbage and RNA was extracted from each sample. For producing the next generation, we continued only with offspring that chose the same plant species as their female parent (developed on cotton and chose cotton or developed on mustard and chose mustard). Significant increase and decrease in gene expression after switching and switching back between Brassicaceae and cotton was observed only for GST2. AFLP analysis revealed that the mustard and cotton lineages were genetically similar (only 2.8% variation among populations from the two plant species), suggesting that no selection occurred in nine generations. When *B. tabaci* adults were fed on artificial diet that contained different glucosinolates degradation products, the highest elevation in GST2 expression was observed when nitriles were added. All together, these findings suggest that GST2 is involved in the response of *B. tabaci* to plants from Brassicaceae and that the insect has the ability to increase the expression level of this gene only when required.

# Sub article: Mating Behavior and its Effect on Reproductive Potential of the B and Q Biotypes of Bemisia tabaci

**DOI:** 10.1673/031.008.0465

**Published:** 2008-01

**Authors:** Shai Morin, Moshe Elbaz

**Affiliations:** Department of Entomology, the Hebrew University of Jerusalem, Faculty of Agricultural, Food and Environmental Quality Sciences, Rehovot, Israel

Ecological interaction between species or sub-species (biotypes), such as reproductive interference, refers to the negative fitness consequences of behavior that disrupts reproduction. Interference may occur between species or biotypes if they have incompletely isolated mate recognition systems. Interspecific reproduction interference includes reduced female reproduction due to mating with heterospecific males, fertilization and aborted development, sperm competition and mate guarding. The whitefly *Bemisia tabaci* has two biotypes in Israel, B and Q. Although the biotypes exist side by side at many locations, accumulated data suggest them to be reproductively isolated. Recent crossing studies between the two biotypes have shown that interbiotype courtship behavior is common. The goal of this study was to check if interbiotype courtship behavior affects the reproductive potential of the B and Q biotypes. We compared the courtship behavior using intra- (B male and B female or Q male and Q female) and inter- (B male and Q female or Q male and B female) biotype pairs. Fecundity was studied using small populations of males and females from one biotype or populations containing females from one biotype and males from both biotypes. Differences were found in the courtship steps and the total courtship period between the intra- and interbiotype pairs. We also observed reduction in the fecundity of Q females in the presence of B males. These results suggest that reproductive interference between the B and Q biotypes of *B. tabaci* exists in at least one direction.

# Sub article: Reversal of Resistance to Pyriproxyfen in the Q Biotype of Bemisia tabaci (Hemiptera: Aleyrodidae)

**DOI:** 10.1673/031.008.0466

**Published:** 2008-01

**Authors:** Shai Morin, Margaret Wilson, Pnina Moshitzky, Einat Laor, A. Rami Horowitz

**Affiliations:** ^1^ Department of Entomology, the Hebrew University of Jerusalem, Faculty of Agricultural, Food and Environmental Quality Sciences, Rehovot, Israel.; ^2^ Department of Entomology, Agricultural Research Organization, The Gilat Research Center, Israel

Pyriproxyfen, a juvenile hormone (JH) mimic, is a biorational insecticide that disrupts insect development. It is one of the principal insecticides being used to control *Bemisia tabaci* (Gennadius) on cotton, and has many environmentally positive attributes, which makes it compatible with integrated pest management (IPM) programs. In Israel, high levels of resistance to pyriproxyfen have been observed in several isolated regions. Here, we tested whether temporal refuges from exposure to pyriproxyfen can be useful for restoring the effectiveness of the compound. We found an eight-fold reduction in resistance when exposure to pyriproxyfen was ceased for 13 generations. Reversal of resistance was accompanied by increased biotic fitness of the revertant colony. By incorporating experimental estimates of nymph survival, sex ratio, fecundity, egg-hatching rate and developmental time, the seasonal cost per generation for resistant insects was estimated to be 25%. A genetic simulation model, optimized by empirical data from bioassays, predicted fitness cost per generation of 19% for resistant homozygous (*RR*) females and hemizygous (*R*) males, and produced rates of reversal similar to the experimental results. The model also predicted that even after five years (∼55 generations) without pyriproxyfen treatments, the frequency of the resistance allele (*R*) will still remain high (0.02). We therefore conclude, based on experimental and modeling results, that the effectiveness of temporal refuges for reversing development of resistance to pyriproxyfen in *B. tabaci* may be limited.

# Sub article: Induced Plant Responses in the Bemisia tabaci Tomato System

**DOI:** 10.1673/031.008.0467

**Published:** 2008-01

**Authors:** Mariano Muñiz, G. Nombela, D. Alonso, A. Gómez

**Affiliations:** Plant Protection Department, Instituto de Ciencias Agrarias - Centro de Ciencias Medioambientales (CSIC), Madrid, Spain.

Some plants have constitutive barriers against possible attacks by pests or pathogens, and plant resistance is an alternative tool to the use of environmentally damaging insecticides in integrated control. In addition, plants can activate protective mechanisms upon contact with a previous invader. This interesting phenomenon is termed induced or acquired resistance and often protects the plant against a second invasion by the same or another pest or pathogen. At times, the induced responses in the plant need not necessarily be negative to subsequent invaders and could produce beneficial effects to such organisms. It is known that some biological agents such as certain bacteria and fungi can induce plant resistance to other pathogens. However, few investigations have been conducted to date to evaluate host-plant induced responses to piercing-sucking insects in susceptible horticultural crop plants. Recently, we have demonstrated that plant resistance to whitefly *Bemisia tabaci* was induced in susceptible tomato plants after infestation by the potato aphid *Macrosiphum euphorbiae,* but not the reverse. Now we present preliminary results from a study investigating whether plant resistance to *B. tabaci* (B biotype) could be induced in susceptible tomato plants (cv. Marmande) by a previous infestation with the same insect pest or, on the contrary, whether responses elicited in the plant would enhance suitability to this whitefly species.

# Sub article: Effects of SB Plant Invigorator® on Bemisia tabaci Development in Tomato Plants

**DOI:** 10.1673/031.008.0468

**Published:** 2008-01

**Authors:** Mariano Muñiz, G. Nombela, A. Gómez, D. Alonso

**Affiliations:** Plant Protection Department, Instituto de Ciencias Agrarias - Centro de Ciencias Medioambientales (CSIC), Madrid, Spain.

Whitefly control programmes are mostly based on the application of insecticides. However, *Bemisia tabaci* is able to develop resistance to many of these chemical substances, and high levels of resistance have been reported for many insecticides in different agricultural systems worldwide. Moreover, many of these chemical products are damaging to the environment if not used properly. These disadvantages make necessary the search for alternative strategies of pest control for implementation in IPM programs. Utilization of new products which fight physically but not chemically against pest is one of the alternative methods investigated nowadays. The Stan Brouard Group has developed a stimulant of plant growth called SB Plant Invigorator® (SBPI®) which helps the plant to produce quality fruits. It presents a physical mode of action that makes the product environmentally safe. Due to its formulation, SBPI® is not a pesticide but a non toxic plant stimulant, No harvest interval is therefore required after use, special health and safety measures for users are not necessary, and the product does not cause problems with residues in food. As its activity against insects is achieved by physical means, insects do not develop resistance to SBPI® and it continues being useful a long time after application. The objective of the present study was to test whether SBPI® is effective to control *B. tabaci* in commercial tomato plants (cv. Marmande) which lack the *Mi-1* gene of resistance to whiteflies. Three days after the first foliar spraying (2ml/l solution), the product seemed to be ineffective against the adult insects, as the numbers of dead females was practically equal on both, control and treated plants. No statistically significant differences were found for egg density although the mean number of eggs laid on SBPI®-treated leaves was slightly greater than that observed on control plants. Treatment was repeated weekly during the next 4 weeks. At 31 days, new adult whiteflies started to emerge from pupae on control, with significant differences with the SBPI®-treated plants where any L3, L4 or new adults were not found. Differences in the number of L1+L2 were not statistically significant, although the mean number of individuals in these larval stages on SBPI®-treated plants was twice as much as on control plants. In conclusion, SBPI® is an effective alternative product to control *B. tabaci* in tomato, because foliar application inhibits or delays larval development, thereby avoiding the risk of a new whitefly generation.

# Sub article: Biological Control of Whitefly in Poinsettia in Ontario, Canada

**DOI:** 10.1673/031.008.0469

**Published:** 2008-01

**Authors:** Graeme D. Murphy, M. Short, A-M Cooper, M. Fast, D. Neal, S. Jandricic

**Affiliations:** ^1^ Ontario Ministry of Agriculture, Food and Rural Affairs, Vineland, Ontario, Canada.; ^2^ Eco Habitat Agri Services, Puslinch, Ontario, Canada; ^3^ Plant Products Co. Ltd. Brampton, Ontario, Canada; ^4^ Global Horticultural Inc. Beamsville, Ontario, Canada; ^5^ Koppert Canada Limited, Scarborough, Ontario, Canada

This presentation describes the results of biological control trials in 13 commercial poinsettia crops in 2006 in Ontario, comparing treated and untreated crops, the use of eggplants as trap plants/banker plants and their impact on the efficacy of biological control. These trials were a cooperative venture among government extension services and industry representatives (including major biocontrol producers, biocontrol distributors and private consultants). In preliminary trials in 2005, whitefly biological control programs were established in 3 commercial poinsettia crops in Ontario, Canada. Trap plants (tomato and eggplant) were used to investigate whether these had potential as either monitoring tools or as banker plants to enhance the regular introductions of insectary-reared insects. Parasitoid wasps, (*Eretmocerus mundus* and *Encarsia formosa*) were released to control *Bemisia tabaci* (= *B. argentifolii*) and *Trialeurodes vaporariorum* respectively. Two rates of introduction were used. A high rate of *Er. mundus* was released in two of the three greenhouses, at a total of approx. 20/m^2^ (approx. 2/ft^2^) over the life of the crop. In the same two greenhouses, *En. formosa* was released at a total of 10/m2 (approx.1/ft2) over the life of the crop. A lower rate of introduction was used in the third greenhouse, with *Er. mundus* and *En. formosa* being released at about one third of the rate described above. At one of the two greenhouses using the high rate of introduction, good control was achieved with no insecticides being applied and only 2% of plants at shipping showing any evidence of whiteflies. In the other two greenhouses, whitefly populations started to build up towards the end of the crop and by mid-October, growers at both facilities began a clean-up spray program using pyridaben. Insufficient introduction rates of parasitoids were considered to be a contributing factor to the whitefly increase in one of these greenhouses, and in the other, there was evidence of pesticide residues as a limiting factor in success. The trap plant trial demonstrated considerable potential as a monitoring tool. Eggplant was significantly more attractive than both poinsettia and tomato to both species of whitefly. Tomato was more attractive to *T. vaporariorum* than poinsettia, but not as attractive to *B. tabaci*. As a banker plant, the trial was less successful, with limited reproduction and colonization of the parasitoids, especially *Er. mundus*. This may have been due to the fact that commercial cultivars of tomato and eggplant were used, which led to management problems in controlling height and vigour. Growers approached this by using strategies such as pinching and excessive use of growth regulators, both of which could have reduced whitefly populations and consequently parasitism. The other issue to note with the use of eggplant is that it is also very effective as a trap plant for thrips, aphids and mites. To respond to these concerns, predatory mites (*Amblyseius cucumeris*) were released preventatively on the trap plants and biocontrol agents for aphids and mites were used as necessary. In 2006, the program was expanded to include a major Ontario propagator of poinsettia cuttings. *Er. mundus* and *En. formosa* were introduced from February to July at a total rate of 15/m^2^ (approx.1.5/ft^2^) and 7.5/m^2^ (approx.0.75/ft^2^) respectively. Until the beginning of June, less than 2% of stock plants were found infested with whiteflies. However, during the month of June this increased to 10%, due in large part to an influx of whiteflies from an adjacent area of the greenhouse where overwintering houseplants had been housed. While not a large infestation, low numbers of whiteflies were found on a large number of plants in a localized area, skewing the final count of infested plants. Encouraging levels of parasitism of *B. tabaci* and *T. vaporariorum* by *Er. mundus* and *En formosa* respectively were evident. Additionally, 13 growers of finished poinsettias agreed to use biological control on their whitefly in the 2006 season (from August-November). Treated crops ranged in size from 1100 m^2^ (12,000 ft^2^) to 13,500 m^2^ (150,000 ft^2^) with a mean of 4100 m^2^ (46,000 ft^2^). A program of introductions was developed based on that used by the successful grower the previous year, with a safety factor of 50% higher introductions built in. Thus, the growers in 2006 worked with total introduction rates of 30 *Er. mundus* and 15 *En. formosa* per m^2^ (approx. 3 and 1.5 per ft^2^ respectively). It was calculated that this would result in a final cost to growers of less than $0.10/pot (based on a 15cm pot), a cost that is competitive with the use of pesticides. Introductions were weighted towards the beginning of the crop with proposed introductions of *Er. mundus* at 3/m^2^/week (approx. 0.3/ft^2^/week) for 6 weeks, followed by 2/m^2^/week (approx. 0.2/ft^2^/week) for a further 6 weeks. Not all growers committed their whole crop to biological control, which allowed comparisons between treated and untreated areas. Eggplants (using a dwarf variety, Stokes Seeds, cv Baby Bell) at a rate of 1/100 m^2^ (approx 1/1000 ft^2^) were used by some of the growers as trap plants/banker plants. In some cases, the whole crop was interplanted with eggplants, and in others, some areas were left untreated, again allowing for comparisons. Results from these trials are presented and discussed in relation to the potential for biological control in commercial poinsettia crops.

# Sub article: Establishment and Impact of Exotic Aphelinid Parasitoids in Arizona: A Life Table Approach

**DOI:** 10.1673/031.008.0470

**Published:** 2008-01

**Authors:** Steven E. Naranjo

**Affiliations:** USDA-ARS, Arid-Land Agricultural Research Center, Maricopa, AZ, USA

A large multi-institutional, interagency classical biological control program was initiated in the early 1990s to combat the invasion of the B biotype of *Bemisia tabaci* into the USA. This large program was successful in the discovery, importation, rearing and release of more than 30 species/strains of aphelinid parasitoids (primarily *Eretmocerus* and *Encarsia*) from around the world into multiple states. Establishment of several species in each targeted state has been documented. However, the impact of these establishments and the overall biological control program has been poorly documented. From 1996 through the present *in situ* life tables have been constructed for *B. tabaci* in cotton and several other important host crops and plants in central Arizona. In the late 1990's, and prior to the documented establishment of two exotic aphelinids in Arizona in the early 2000's (*Er.* nr. *emiratus* and *En. sophia*), parasitism contributed a consistently small amount of mortality to *B. tabaci* in cotton fields. Subsequent life tables in cotton and a number of other host crops and plants showed that levels of parasitism were generally enhanced within a multi-crop system and that these increased levels of mortality coincided with the documented establishment of two exotic species. However, life table studies in cotton over the past few years have been inconclusive relative to impact, with levels of mortality from parasitism fluctuating widely between low and moderate. Further life table analyses (key factors, density dependence) are planned and should help to interpret and quantify the potential impact of these introductions on whitefly population dynamics.

# Sub article: Conservation of Natural Enemies Through use of Selective Insecticides: Recent Developments

**DOI:** 10.1673/031.008.0471

**Published:** 2008-01

**Authors:** Steven E. Naranjo, Peter C. Ellsworth

**Affiliations:** ^1^ USDA-ARS, Arid-Land Agricultural Research Center, Maricopa, AZ, USA.; ^2^ Department of Entomology, University of Arizona, Maricopa Agricultural Center, Maricopa, AZ, USA

It has been well established that the use of selective insecticides promotes the critical role of biological control in the successful management of *Bemisia tabaci* within the Arizona Cotton IPM program. An increasing number of putatively selective insecticides have been introduced in recent years, but their potential role in replacement or alternative IPM strategies are unknown. Field studies were undertaken to evaluate and quantify the selectivity of acetamiprid (neonicotinoid), spiromesifen (lipid synthesis inhibitor) for the management of whitefly in cotton. A two year study showed that use of acetamiprid resulted in the decline of fewer taxa of natural enemies compared with conventional, broad-spectrum materials, but that the levels of reduction were similar in taxa negatively affected by both insecticide regimes. The grower-standard insect growth regulators (IGRs) buprofezin and pyriproxyfen were confirmed to be highly selective. An on-going commercial-scale study demonstrated that spiromesifen appears to be equally selective as the IGRs but that selectivity of this new insecticide is dosage dependent. An additional year of field study with spiromesifen and NI-0101 (a new putatively selective compound from Nichino America) is underway in 2006. Results from the acetamiprid study and the preliminary results from the spiromesifen study have led to revision of recommended chemistry within the cotton IPM program. Low to moderate doses of spiromesifen are suggested as an alternative to the IGRs in Stage I of the plan where selectivity is of critical importance. Acetamiprid is suggested for use in Stage II of the IPM plan which promotes the rotation of chemistry across the season, provides for the usage of insecticides with low to moderate levels of selectivity, and eliminates the usage of broad-spectrum materials such as pyrethroids until late in the growing season.

# Sub article: Mortality and Population Dynamics of Bemisia tabaci within a Multi-Crop System

**DOI:** 10.1673/031.008.0472

**Published:** 2008-01

**Authors:** Steven E. Naranjo, Peter C. Ellsworth, Luis Cañas

**Affiliations:** ^1^ USDA-ARS, Arid-Land Agricultural Research Center, Maricopa, Arizona, USA.; ^2^ Department of Entomology, University of Arizona, Maricopa Agricultural Center, Maricopa, Arizona, USA; ^3^ Department of Entomology, Ohio State University, OARDC, Wooster, Ohio, USA

*Bemisia tabaci* is a multivoltine insect with no diapause that maintains population continuity by moving from one host to another over the year. To better understand the mechanisms governing population development of this insect, whitefly “ecosystems” were established in three geographically and climatically distinct areas in Arizona. These systems consisted of a sequence of six representative hosts including winter broccoli, spring and fall cantaloupes, summer cotton, perennial alfalfa, various annual weeds, and the perennial ornamental, lantana. No insecticides were used at any site. Source and rates of natural mortality were quantified, *in situ*, on each host plant by constructing life tables. The same mortality factors were observed in all hosts at all sites. The quantitative contribution of each mortality factor varied among hosts and time of the year, but was relatively consistent among geographic sites. Predation and dislodgement from the plant surface were consistently the largest sources of mortality, although mortality from desiccation was high during the fall and winter. Most mortality occurred during the 4th nymphal stadium and the egg stage for most host plants. Median generational mortality was generally > 90% except on spring-planted cantaloupe where median mortality was ≈ 65%. Predation, dislodgement, desiccation, and occasionally parasitism, were the key factors depending on host plant, season and site. Low mortality during the spring on cantaloupe appears to act as a biotic release leading to outbreak populations during summer months. Overall, an understanding of the year round dynamics of this pest will greatly aid the development of better pest management strategies in all affected crops.

# Sub article: Greenhouse Whitefly Eggs and Nymphs Density on Gerbera jamesonii under Different Nitrogenous Fertilizer Regimes

**DOI:** 10.1673/031.008.0473

**Published:** 2008-01

**Authors:** Laura D. Ortega-Arenas, D. A. A. Miranda, M. Sandoval-Villa

**Affiliations:** ^1^ Programa en Entomología, Campus Montecillo, Colegio de Postgraduados, Montecillo, Estado de México. México.; ^2^ Programa en Edafología, Campus Montecillo, Colegio de Postgraduados, Montecillo, Estado de México. México

The objective of the study was to evaluate the influence of nitrogenous fertilization in three varieties of gerbera (*Gerbera jamesonii* H. Bolus), on the oviposition rate and population density of whitefly nymphs, *Trialeurodes vaporariorum* (West.). An evaluation was also made of the effect of nitrogen concentration on indicators of flower quality (diameter of capitulum, diameter of peduncle and length of penduncle), yield and post-harvest life. Three varieties of gerbera were used (Tzigana, Fallela and Marisol) and three concentrations of nitrogen (8, 12 and 16 me L^-1^), in a completely random design with factorial arrangement of treatments. The effect of the treatment was determined registering every two days, the population density of whitefly eggs and nymphs, per cm of leaf, in two experiments: 1) free choice and 2) no choice. The population density of eggs and nymphs was higher in free choice assays. In these, the number of eggs and nymphs increased significantly (p≤0.05) as the supply of nitrogen increased. In the no choice assays, however, the oviposition rate and nymphs density were not notably influenced by the nitrogen supply. In the three varieties, the highest density of eggs and nymphs was related to the nitrogen content in the leaves. The nitrogen supply did not effect the diameter of the capitulum, the length and thickness of the peduncle and the yield, but it did have an influence on the post-harvest life of the flower. In the varieties Fallela and Marisol, the post-harvest life was longer as the nitrogen supply was increased; on the other hand, in the Tzigana variety, the duration of the flower did not show a direct relationship with the nitrogen content.

# Sub article: Invasion and response: Impacts of bemisia on worldwide agriculture

**DOI:** 10.1673/031.008.0474

**Published:** 2008-01

**Authors:** L. S. Osborne

**Affiliations:** Mid-Florida Research and Education Center, IFAS, University of Florida, Apopka, FL, USA.

The historical record reveals that *Bemisia tabaci* has caused damage to agricultural crops for more than 80 years. However, this pest was generally considered a minor or even a non-pest in most agricultural systems until the late 1980's and early 1990's. During the past 20 years the impact this pest has had on agriculture has been staggering. Why, after almost 80 years, has this whitefly reached such a pest status that we are attending the 4th International Workshop to discus its management and biology? The two workshops in Duck Key this week will address some of the issues that have contributed to this heighten pest status. During my presentation, I will discuss the impact this pest has had on agriculture and why we should pay very close attention to *Bemisia tabaci* This insect should severe as a model of an invasive species. Why has this pest become so invasive? What can we learn from the worldwide invasion of the B and Q biotypes that can be useful in mitigating new invasions? Can we learn lessons so that we won't be attending International Workshops on other common pests such as *Frankliniella occidentalis* (Pergande), *Liriomyza trifolii* (Burgess), *Aphis gossypii* Glover or *Myzus persicae* (Sulzer) in the near future? During the period from 1986 to 2000, 150 new exotic arthropods found their way into Florida and became established (Thomas 2000). *Bemisia tabaci* is not listed in this document probably because it was not treated as an actionable or unique insect in Florida or the United States. Contaminated imports were allowed to proceed to their destination unless a significant infestation was detected. What was different about existing populations of *B. tabaci* (probably A biotype) and the populations that made their why into Florida in the 1980's (probably B-biotype)? It has been postulated that sensitivity to pesticides, reproductive capacity, host range, and “toxic” salivary secretions differ between the biotypes. Most of these factors probably contributed to the displacement of the A-biotype and the elevation of *Bemisia tabaci* to one of the most serious pests of commercial agriculture. The one contributing factor that we influence by our actions is pesticide resistance. All things being equal, a resistant strain has a significant competitive advantage in agriculture systems that predominantly rely on pesticides to manage pests. We have not, nor do we currently regulate arthropods below the species level. The detection of the Q-biotype has brought this topic into sharper focus and fostered an evaluation of the issue. I will discuss how regulating arthropods at a level that requires specialized and often slow techniques for identification could negatively impact our ability to protect agriculture.

# Sub article: Managing Bemisia on Organically Grown Herbs

**DOI:** 10.1673/031.008.0475

**Published:** 2008-01

**Authors:** L. S. Osborne, G. J. Hochmuth, R. C. Hochmuth, D. Sprenkel

**Affiliations:** ^1^ Mid-Florida Research and Education Center, IFAS, University of Florida, Apopka, FL, USA.; ^2^ Office of the Dean for Research, IFAS, University of Florida, Gainesville, FL, USA; ^3^ North Florida Research and Education Center, IFAS, University of Florida, Live Oak, FL, USA; ^4^ North Florida Research and Education Center, IFAS, University of Florida, Quincy, FL, USA

*Bemisia tabaci* (Q and B-biotypes) is a limiting factor in the production of organically grown spearmint. Very few pesticides are registered for use on this crop in greenhouses and those that we have tested have caused significant phytotoxicity or, at safe rates, did not provide acceptable levels of control. Biological control has also been evaluated. During 2005, *Eretmocerus eremicus* was eliminated as a potential candidate because of economics, poor establishment and efficacy. *Eretmocerus mundus* was selected for use in trials during December 2005. Three shipments were obtained for use at three Florida study sites (Apopka, Live Oak and Quincy). Only one shipment contained sufficient parasitoids for the studies. In the other two shipments, less than 25% of the parasitoids emerged. Current studies are focused on further evaluations of *E. mundus, Amblyseius swirskii* and *Encarsia sophia.* We are also looking at various pesticides that could be integrated with these natural enemies.

# Sub article: Cross-commodity Guidelines for Neonicotinoid Insecticides in Arizona

**DOI:** 10.1673/031.008.0476

**Published:** 2008-01

**Authors:** J. C. Palumbo, P. C. Ellsworth, A. Fournier, T. J. Dennehy, R. L. Nichols

**Affiliations:** ^1^ Department of Entomology, University of Arizona, Tucson, AZ, USA.; ^2^ Maricopa Agricultural Center, University of Arizona, Maricopa, AZ, USA; ^3^ Cotton Incorporated, Cary, NC, USA

Arizona is enjoying a sustained recovery from the devastating silverleaf whitefly (*Bemisia tabaci*) outbreaks of the early 1990s. This success is built on an integrated pest management (IPM) strategy that includes the use of selective and effective chemistry. Admire has been a key soil insecticide, protecting vegetables and produce throughout Arizona, and is the first member of a burgeoning class of chemistry known as the neonicotinoids. New members of this valuable, reduced-risk, class of chemistry are now available to agricultural producers, placing a burden on users of these compounds to adopt science-based plans for sustaining their efficacy. This consensus document represents our best efforts to share this chemistry among different agricultural interests. Our goal is to preserve the long-term efficacy of the neonicotinoids and protect growers' interests in sustainable and economical whitefly management. Through identification of crop communities (i.e., ‘multi-crop’, ‘cotton-intensive’, and ‘cotton/melon’) common to Arizona agriculture, we have designed sensible plans of use that should allow access to this valuable chemistry for everyone, while protecting it from resistance.

# Sub article: The Effect of Begomoviruses on Whitefly Fitness

**DOI:** 10.1673/031.008.0477

**Published:** 2008-01

**Authors:** J. E. Polston, M. Toapanta

**Affiliations:** ^1^ University of Florida/IFAS, Department of Plant Pathology, Gainesville, FL, USA.; ^2^ Bayer Cropscience, Tampa, FL, USA

The fitness of the whitefly *Bemisia tabaci*, biotype B, on virus-free tomato plants was compared to the fitness of those inoculated with *Tomato yellow leaf curl virus* (TYLCV) or *Tomato mottle virus* (ToMoV), begomoviruses which infect tomato. The fecundity (number of eggs oviposited) of *B. tabaci* on cabbage, a non-host for either virus, was similar regardless of whether whiteflies were reared on virus-free tomatoes, ToMoV or TYLCV-infected tomatoes. However, begomovirus-infected tomatoes affected both development and reproduction parameters of whiteflies which were reared on cabbage and then allowed to oviposit on virus-free and virus-infected tomatoes. Both fecundity and fertility of whiteflies were significantly higher on virus-free tomatoes than on ToMoV- or TYLCV-infected tomatoes. TYLCV-infected tomatoes had the lowest number of eggs oviposited and the lowest percent of eggs hatched. Moreover, the development time of whiteflies from egg to adulthood was shorter on virus-free plants than the development time of whiteflies on begomovirus-infected tomatoes. These preliminary results suggest that neither begomovirus has a direct positive or negative impact on the fitness of their whitefly vector. The effect of begomoviruses on whitefly fitness and life history appears to be indirect, and more a consequence of the reduced suitability of the host plant for whitefly development and reproduction.

# Sub article: CORAGEN™ SC (DPX-E2Y45): a Novel Anthranilamide Insecticide: Pest Spectrum with Emphasis on Whitefly Control in Vegetables

**DOI:** 10.1673/031.008.0478

**Published:** 2008-01

**Authors:** Hector Portillo, Paula C. Marçon, Robert W. Williams, Danny Tamayo, Daniel W. Sherrod, Fred W. Marmor

**Affiliations:** ^1^ DuPont Crop Protection, Stine Haskell Research Center, Newark, USA.; ^2^ DuPont Crop Protection, Bradenton, FL USA; ^3^ DuPont Crop Protection, Yuma, AZ, USA; ^4^ DuPont Crop Protection, Memphis, TN, USA

The anthranilamide class of insecticides is a novel chemistry that acts with outstanding selectivity on the ryanodine receptor in insects. CORAGEN™ SC is an anthranilamide discovered and currently being commercialized by Dupont Crop Protection. CORAGEN™ SC is highly potent and has shown broad-spectrum control of Lepidopteran crop pest species. In addition to its outstanding activity on Lepidoptera, CORAGEN™ SC has shown varied levels of efficacy on selected crop pest species in the order Coleoptera, Diptera, and Homoptera. Within Homoptera, CORAGEN™ SC has been most consistent in providing efficacy and crop protection against key whitefly species, i.e., *Bemisia tabaci* and *B. argentifolii.* Under laboratory and field conditions, the level of efficacy on whiteflies with CORAGEN™ SC varies with the method of application (i.e., foliar spray versus root uptake), the ability of the foliar spray to penetrate the leaf cuticle (translaminar movement), the intensity of the infestation, and the timing of the application in relation to pest pressure. CORAGEN™ SC acts primarily via ingestion on whitefly nymphs. Although adult whiteflies are not directly controlled, feeding behavior and reproduction are affected. Field efficacy tests conducted around the world indicate that in addition to its outstanding efficacy on Lepidopteran pests, CORAGEN™ SC can be a valuable tool for the management of key whitefly species, providing inhibition of virus transmission and overall crop protection. Laboratory and field efficacy results on fruiting vegetables and cucurbit crops will be presented.

# Sub article: Spiromesifen: A New Pest Management Tool for Whitefly Management

**DOI:** 10.1673/031.008.0479

**Published:** 2008-01

**Authors:** Nilima Prabhaker, Nick C. Toscano

**Affiliations:** Department of Entomology, University of California, Riverside, CA, USA.

During the last two decades, there have been a number of changes in whitefly management worldwide. Previously broad-spectrum insecticide applications were recommended, based on the population dynamics of whiteflies, leading to resistance problems in some cases. At present, a number of options in chemical control are available for managing whiteflies through development of integrated pest management programs that include use of safer, more selective insecticides with narrow spectrum of activity that may be compatible with beneficial insects. Some of the newer insecticides, including insect growth regulators, neonicotinoids, sodium channel blockers and tetronic acids, are more selective and reported to be compatible with use of many kinds of natural enemies. The enhancement of natural enemy populations in a cropping system can add additional mortality factors and promote integration of chemical and biological control components of IPM programs. Spiromesifen is a member of a recently introduced class of selective chemistry, tetronic acids, with broad spectrum insecticidal and acaricidal activity against whiteflies and spider mites. Its mode of action is by inhibition of lipid biosynthesis that affects the egg and immature stages of these pests. Foliar sprays of spiromesifen are reported to be effective against whiteflies in cotton, vegetables and ornamentals. The present study was conducted to evaluate the efficacy of spiromesifen against a number of natural populations of *Bemisia argentifolii* from California and Arizona and against imidacloprid-resistant whiteflies, to establish baseline toxicity data. Baseline toxicity data for spiromesifen against *B. argentifolii* populations have been collected for consideration as part of future whitefly resistance management program. The test populations were all ‘B’ biotypes with the exception of a ‘Q’ biotype from Spain held in quarantine at UC Riverside. The compound was effective against all immature stages of whiteflies but exhibited geographical susceptibility differences. However, spiromesifen was significantly more active against early instars of whiteflies based on lowest LC50s recorded. Spiromesifen was highly effective against the ‘Q’ type strain from Spain, which is highly resistant to neonicotinoids, suggesting the absence of cross-resistance between the two classes of chemistry. Spiromesifen also showed reduced oviposition and egg hatch in an imidacloprid-resistant strain. Spiromesifen appears to be safe to *Eretmocerus eremicus* under greenhouse conditions. Results of this study suggest that spiromesifen has no cross-resistance to more commonly used neonicotinoids and appears to provide a more compatible environment for biological control of whiteflies. Therefore, spiromesifen can be added to the arsenal of chemistries that are currently available for whitefly management. Spiromesifen can be considered to be an ideal candidate for whitefly resistance management programs in rotation with chemistries with other modes of action and minimize current problems in controlling whiteflies in various parts of the world.

# Sub article: Whitefly (Bemisia tabaci) Transmitted Squash Vein Yellowing Virus (SqVYV): A Component of Watermelon Vine Decline in South Florida

**DOI:** 10.1673/031.008.0480

**Published:** 2008-01

**Authors:** P. D. Roberts, Philip A. Stansly, Scott Adkins, Susan Webb, Carlye Baker, Benny Bruton, Rosa Muchovej, Diann Achor, C.S. Kousik, Alicia L. Whidden, Gene McAvoy, Phyllis Gilreath

**Affiliations:** ^1^ University of Florida, Department of Plant Pathology, SWFREC, Immokalee, FL, USA.; ^2^ University of Florida, Department of Entomology & Nematology, Immokalee, FL, USA; ^3^ United States Department of Agriculture-ARS, USHRL, Fort Pierce, FL, USA; ^4^ University of Florida, Department of Entomology & Nematology, Gainesville, FL, USA; ^5^ Florida Department of Agriculture, Division of Plant Industry, Gainesville, FL, USA; ^6^ United States Department of Agriculture-ARS, Lane, Oklahoma, USA; ^7^ University of Florida, CREC, Lake Alfred, FL, USA; ^8^ US Vegetable Lab, USDA, Charleston, SC, USA; ^9^ University of Florida/IFAS, Hillsborough County Extension Service, Seffner, FL, USA; ^10^ University of Florida/IFAS, Hendry County Extension Service, LaBelle, FL, USA; ^11^ University of Florida/IFAS, Manatee County Extension Service, Palmetto, FL,USA

Watermelon production has been greatly impacted in South Florida since 2003 when symptoms were first observed of a severe watermelon vine decline (WVD) that killed plants as the crop approached first harvest (Roberts et. al., 2005 Phytopathology 95:S89.; Huber 2006, Citrus and Vegetable Magazine, 70(6): 22–24). Since 2003, watermelon plants have been affected in each fall (September–December) and spring (January–May) season. Symptoms of WVD occur as fruit are approaching harvest or soon thereafter. Foliage initially turns a slight yellow, then scorched and brown, followed by wilting and collapse of the entire vine. The xylem exhibits a uniform dark tan to light brown discoloration which is distinct from the discoloration of the xylem caused by races of *Fusarium oxysporum* f. sp. *niveum*. Although there are no external symptoms, the fruit on the declining vines frequently have rinds with greasy and discolored (brown and/or watersoaked) internal blotches. The non-macerated flesh is distinctly off-flavor, inedible, and non-marketable. However, symptoms of WVD are variable in that foliage decline symptoms can occur in the absence of fruit rot symptoms and vice-versa. Disease progress is normally very rapid although variations in the rate have also been observed. In some fields, vine decline increased from 10% affected plants to greater than 80% within a week. In other fields, the disease progressed through the yellowing, wilting phase and then stopped. Yield losses have been typically high; losses of 50%–100% are not uncommon in affected fields. The disease has occurred at epidemic levels in southwest and south central Florida since 2003 and poses a significant threat to production of this economically important crop. Losses to Florida growers are estimated at more than $60 million in the past four years (Huber, 2006). Abiotic and biotic causes of WVD were examined. Nutritional testing indicated soil and plant tissue nutritional deficiencies in most samples collected from affected fields. However, results varied from site to site, indicating that deficiencies may only have contributed to weakening the plants, thus increasing their susceptibility to disease. Examination of cultural practices did not uncover common links to fungicides, insecticides, herbicides, irrigation, cultivars, transplant production, fumigation, or other practices. Likewise, environmental conditions (rainfall, temperature) were variable from season to season, and while it does not appear that there is an environmental cause of this disease, the environment may play a role in WVD. Several fungi and bacteria have been recovered from affected vines and fruit which caused mortality of watermelon seedlings and decay of fruits in controlled environmental studies. *Plectosporium tabacinum* was frequently isolated from the xylem of affected plants that caused a seedling disease under greenhouse conditions. Several *Fusarium, Pythium, Phytophthora*, and *Rhizoctonia* spp. have been recovered but none of these were consistently isolated from symptomatic plants. Although race 0, 1, and 2 of *Fusarium oxysporum* f. sp. *niveum* were often isolated from symptomatic plants, symptoms of WVD are distinct from Fusarium wilt. Strains of *Serratia* and *Pseudomonas* spp. and an unidentified bacterial strain caused tissue maceration, necrosis, or brown blotches on fruit rind similar to field symptoms. However, neither fungal nor bacterial pathogens appeared to be the primary cause of WVD, but the role of these and other factors continue to be investigated. Recent research by Adkins and colleagues (2006) identified a novel ipomovirus dubbed *Squash vein yellowing virus* (SqVYV) associated with WVD. In addition, they showed that SqVYV was transmitted by the silverleaf whitefly *Bemisia tabaci* (Biotype “B”). Watermelon death and fruit rot symptoms of WVD were evident in SqVYV greenhouse-inoculated plants (Adkins et al. 2006, Phytopathology 96:in press). A field study conducted in the spring 2006 at SWFREC showed that watermelon plants grown adjacent to SqVYV-infected squash plants developed vine decline symptoms, including fruit rot, and eventually died. Plants which were grown in the same field, but protected by screened walk-in cages to prevent infestation by whitefly, did not develop symptoms of vine decline. Initial analysis of declining watermelon plants in the field study confirmed the presence of SqVYV in these plants. Insecticide applications of Admire and Fulfill did not prevent vine decline on treated plants. This failure could have been due to inadequate whitefly control obtained in this field trial, or, as is the case with many potyviruses transmitted by aphids, due to the inability of insecticides to act quickly enough to prevent virus transmission. Experiments to confirm these preliminary field results and target management of WVD by insecticidal control of whiteflies are being conducted.

# Sub article: Imidacloprid Resistance of Bemisia tabaci in Crete, Greece

**DOI:** 10.1673/031.008.0481

**Published:** 2008-01

**Authors:** Emmanouil Roditakis, Nikos E. Roditakis, Maria Grispou, John Vontas, Anastasia Tsagkarakou

**Affiliations:** ^1^ Laboratory of Entomology and Agricultural Zoology, Plant Protection Institute of Heraklion, National Agricultural Research Foundation, Heraklion, Crete, Greece.; ^2^ Laboratory of Pesticide Science, Agricultural University of Athens, Athens, Greece

*Bemisia tabaci* (Homoptera: Aleyrodidae) is considered a major pest of both vegetable and flower crops in Crete, Greece. The first severe pest management problems were reported after repeated failures to control the pest during a sudden TYLSV outbreak in 2001. In a survey during 2002–03, high resistance levels were recorded against pyrethroids and neonicotinoids in whitefly populations (Biotype Q) collected from greenhouses practicing conventional pest management. In 2005 and 2006, during an extensive resistance monitoring survey for imidacloprid resistance, toxicological bioassays were preformed on 41 whitefly populations, collected from greenhouses located in the main vegetable producing areas in southern Crete at the end of the growing season (May – June). LC50 ranged from 14 to 267 mg a.i./l in 2005 and from 24 to 705 mg a.i./lt in 2006, indicating a rapid progress of the resistance problem in the *B. tabaci* populations of Crete. Studies on the mechanisms of resistance to imidacloprid showed that P450 monooxygenase activity with the substrate ethoxycoumarine was substantially elevated in a highly resistant strain, compared to the susceptible strain SUD-S. Using the metabolic inhibitors piperonyl butoxide (PBO) and S,S,S-tributyl phosphorotrithioate (DEF) the implication of cytochrome P450-dependent monooxygenases was confirmed; however, the mortality was still lower than that of the susceptible strain, suggesting the possible involvement of additional resistance mechanisms.

# Sub article: The Inside Story: Bacteriocyte-associated Endosymbionts of Whiteflies

**DOI:** 10.1673/031.008.0482

**Published:** 2008-01

**Authors:** Rosemarie C. Resell, D. R. Frohlich, H. S. Costa, M. Coombs, P. DeBarro

**Affiliations:** ^1^ Biology Department, University of St. Thomas, Houston, TX, USA.; ^2^ Entomology Department, University of California, Riverside, CA, USA; ^3^ CSIRO, Brisbane, QLD, Australia

Whiteflies, members of the Sternorrhyncha that feed exclusively on phloem sap, harbor bacterial endosymbionts within specialized insect cells, bacteriocytes, which aggregate in the abdominal cavity to form the bacteriome. In whiteflies, the P-endosymbionts have been designated as “*Candidatus* Portiera aleyrodidarum”. It has been hypothesized that the pleomorphic bacteria present in whitefly bacteriocytes are the primary endosymbionts. The P-endosymbiont phylogeny has been reconstructed based on either 16S rDNA or 16S-23S rDNA and results support the separation of whiteflies into two subfamilies. In addition, *Bemisia tabaci* may house several secondary endosymbionts, including *Wolbachia*, aphid T-type endosymbionts and *Arsenophonus* (A-type endosymbionts) and *Candidatus* Fritschea bemisiae (chlamydiae). This study focuses on the endosymbionts from a broad range of whitefly species collected primarily in Australia and the U.S. The goals are threefold: 1) to further extend the phylogeny of whitefly P- and S-endosymbionts using 16SrDNA sequences from these whitefly species, 2) to examine phylogenetic congruence between the symbionts and the host, and 3) to morphologically characterize both P- and S-endosymbionts in these whitefly species. Phylogenetic analyses of P-endosymbiont 16S rDNA sequences from 50 whitefly species are partially consistent with previously published phylogenies. The data are unique in that we include the largest group of taxa and examine individual variability within each taxon instead of consensus data. The G+C content of our P-endosymbiont DNA samples ranged from 46.46 to 48.77 mol%, which is in the same scale as previous reports for *Candidatus* Portiera. In our morphology studies, yellow-orange bacteriomes were clearly visible through the 4^th^ instar cuticle of some species, while in others there was no discernible coloration to indicate the presence of bacteriomes. This could simply mean that bacteriocytes are not as strongly pigmented in these species. In whitefly species that had a hard black cuticle in the immature stages, individuals were peeled off of the leaf and observed from beneath. Again, the visibility of bacteriomes varied among species. Thus far, we have examined the bacteriomes from 11 species using TEM. Visually the bacteria present are similar to those that previously described in *B. tabaci*. At least two morphological types of endosymbionts (P and S) were present in each species examined. Labeling studies are in progress to correlate morphology with species designation.

# Sub article: Arthropod Predation on Bemisia tabaci Cassava in Uganda: Preliminary Results from Molecular Gut Analysis

**DOI:** 10.1673/031.008.0483

**Published:** 2008-01

**Authors:** Daniel P. Rowley, Peter Asiimwe, James P. Legg, Matthew H. Greenstone

**Affiliations:** ^1^ Insect Biocontrol Laboratory, USDA-Agricultural Research Service, Beltsville, MD, USA.; ^2^ Eastern and Southern Africa Regional Center, International Institute of Tropical Agriculture, Kampala, Uganda

Cassava is second only to maize as a staple crop in Sub-Saharan Africa. *Bemisia tabaci* is the principle vector of African cassava mosaic disease (ACMD), which is responsible for greater reductions in cassava yield than any other pest in the region. As part of IPM research to reduce the incidence of ACMD, we are examining the potential of predators to suppress *B. tabaci* populations. Predators that we observed feeding on the whitefly include ants, coccinellid adults and larvae, *Conwentzia* larvae, and syrphid larvae. Using PCR primers designed to distinguish *B. tabaci* DNA from that of two abundant alternate prey, cassava green mite and cotton aphid, we screened a sample of all of these predators, and found positives only in syrphid larvae and larvae of an undescribed coccinellid of the genus *Serangium.* A larger sample disclosed no positives in *Serangium* adults (N=25), 8.3 % positives in *Serangium* larvae (N=24), and 92% positives in the syrphid larvae (N=24). Since the detectability interval for DNA can affect the interpretation of prey positives, we are performing *B. tabaci* half-life studies in these predators so that we can correctly weight these data and determine the relative importance of these two predators as natural enemies of *B. tabaci*.

# Sub article: Monitoring of Imidacloprid Resistance in Biotype B of Bemisia tabaci Florida

**DOI:** 10.1673/031.008.0484

**Published:** 2008-01

**Authors:** D. J. Schuster, R. S. Mann, Marco Toapanta, R. Cordero, S. Thompson, R. F. Morris

**Affiliations:** ^1^ University of Florida, IFAS, Gulf Coast Research and Education Center, Wimauma, FL, USA.; ^2^ B & W Quality Growers, Inc., Fellsmere, FL, USA; ^3^ Bayer CropScience, Lakeland, FL, USA

Biotype B of the sweetpotato whitefly, *Bemisia tabaci* (Genn.), is a worldwide pest that has developed resistance to many insecticides, including the novel neonicotinoid class. Field populations were collected from 2000 to 2006 from different locations in Florida and were compared for their susceptibility to the neonicotinoid imidacloprid (Bayer Crop Science) using a cut leaf petiole bioassay method. Three whitefly populations collected in 2000 showed high susceptibility to imidacloprid, with a maximum resistance ratio (RS50) of 6.00. Whitefly susceptibility was lower in 2001, 2002 and 2003 with 3 out of 12 bioassayed populations indicating RS50 values over 10.0 during 2001, 4 out of 15 during 2002 and 14 out of 21 during 2003. The maximum resistance ratio also showed a similar pattern with RS50 values of 14.6, 35.2 and 28.1 for 2001, 2002 and 2003, respectively. The populations showed increased susceptibility during 2004 and 2005 with only 2 populations out of 17 recording RS50 values over 10.0 during 2004 and none out of 2 during 2005. The maximum RS50 value also decreased to 11.4 during 2004 and 2.59 during 2005. Whitefly populations collected during 2006 again showed reduced susceptibility, with 6 out of 8 populations recording RS50 values above 10.0 and with the maximum RS50 value being 45.51. Rearing whitefly populations in the laboratory for 4–6 generations without exposure to imidacloprid resulted in RS50 values declining by 75 to 80 per cent to acceptable levels during 2001 and 2002. Similar decreases are also being observed with 2006 populations. All of the 2006 populations were tested and confirmed to be biotype B. Therefore, biotype B possesses the potential to develop unstable tolerance to imidacloprid, and monitoring for imidacloprid resistance needs to continue in Florida.

# Sub article: DuPont Rynaxypyr™ (DPX-E2Y45): A Novel Anthranilamide Insecticide for Managing Bemisia tabaci and Interfering with Transmission of Tomato Yellow Leaf Curl Virus on Tomato Transplants

**DOI:** 10.1673/031.008.0485

**Published:** 2008-01

**Authors:** David J. Schuster, Natália A. Peres, Robert W. Williams, Paula C. Marçon, Hector E. Portillo

**Affiliations:** ^1^ University of Florida/IFAS, Gulf Coast Research & Education Center, Wimauma, FL, USA.; ^2^ Dupont Crop Protection, Raleigh, NC, USA; ^3^ Dupont Crop Protection, Stine-Haskell Research Center, Newark, DE, USA

Rynaxypyr™ (DPX-E2Y45) is a novel insecticide from the anthranilamide class of chemistry, discovered and being commercialized by Dupont Crop Protection. This new insecticide provides effective control and good residual activity against a broad spectrum of economically important pest species at low use rates. Greenhouse studies were conducted to determine the efficacy of Rynaxypyr™ in controlling *Bemisia tabaci* biotype B and in preventing transmission of the begomovirus *Tomato yellow leaf curl virus* (TYLCV). Tomato seedlings were grown in transplant trays and, at the 2-leaf stage, insecticide treatments were applied either as soil drenches or foliar applications. (I) To evaluate the direct effects of the treatments on adult whitefly mortality, insecticide treated seedlings were transplanted into pots (7 days after the drench treatment or 1 day after the foliar treatment). Ten non-virulent whitefly adults were confined on one leaf of each plant in clip cages and the number of surviving adults was counted daily for 3 days. (II) To evaluate the indirect effects on inhibition of virus transmission, treated plants were exposed to virulent TYLCV whitefly adults in organdy cages for 3 days, after which adults were removed. Plants were held for an additional 3 weeks and were periodically inspected for obvious TYLCV symptoms. The presence or absence of TYLCV was confirmed by PCR using TYLCV specific primers. Rynaxypyr™ was not as effective as the standard pymetrozine as a foliar spray or the standard imidacloprid as a drench in killing whitefly adults and in reducing TYLCV transmission. Nevertheless, the level of suppression provided by foliarly applied Rynaxypyr™ was significant and comparable to current control levels observed with neonicotinoids in the field. These data justify inclusion of this novel insecticide with a new mode of action for foliar chemical rotations and alternative drench treatments as part of a resistance management program.

# Sub article: Management of Bemisia, TYLCV, and Insecticide Resistance in Florida Vegetables

**DOI:** 10.1673/031.008.0486

**Published:** 2008-01

**Authors:** David J. Schuster, Philip A. Stansly, Phyllis R. Gilreath, Jane E. Polston

**Affiliations:** ^1^ Univeristy of Florida/IFAS, Gulf Coast Research & Education Center, Wimauma, FL, USA.; ^2^ University of Florida/IFAS, Southwest Florida Research & Education Center, Immokalee, FL, USA; ^3^ University of Florida/IFAS, Cooperative Extension Service, Manatee County, Palmetto, FL, USA; ^4^ University of Florida/IFAS, Department of Plant Pathology, Gainesville, FL, USA

Staked tomatoes and other fruiting vegetables destined for the fresh market are grown outdoors primarily in four distinct regions of southern Florida. Greenhouse-grown seedlings are transplanted into raised beds covered with polyethylene mulch in late summer/early fall and again in mid-winter. Thus, crops may be in the field almost year round, with the exception of early to mid summer. The sweetpotato whitefly (*Bemisia* spp.) biotype B, also known as the silverleaf whitefly, is a key pest of tomatoes and other vegetables grown in southern Florida. Although the whitefly induces an irregular ripening disorder of tomato fruit, most damage results from the transmission of *tomato yellow leaf curl virus* (TYLCV). Various insecticides in the neonicotinoid class have been relied upon heavily for the past 12 years to manage the whitefly. To ensure the continued viability of this class of insecticides as well as that of other insecticide classes, resistance management recommendations have been prepared. Although insecticide-use practices are an integral part of the management recommendations, it is recognized that they cannot be successful without adherence to integrated pest management practices. Therefore, recommendations include field hygiene and other cultural practices which are considered high priority and which are included as integral parts of the overall strategy for managing whitefly populations, TYLCV incidence, and insecticide resistance. These practices include: establishment of a two month crop-free period in the summer; prompt, efficient and thorough destruction of crops within 5 days of final harvest; use of whitefly and virus-free transplants; separation of new crops from old crops; use of TYLCV-resistant tomato and pepper cultivars; use of determinant cultivars of grape tomatoes; use of UV-reflective plastic soil mulch; applications of effective insecticides prior to pruning, tying, harvest, etc.; removal and destruction of tomato plants with symptoms of TYLCV until the second tie; management of weeds within crops; proper disposal of tomatoes culled from packing houses and avoidance of post-harvest gleaning operations. Insecticide-use practices include: avoidance of neonicotinoid use on transplants in production facilities; use of neonicotinoids in the field only during the first six weeks following transplanting; use of insecticides in classes other than neonicotinoids six weeks after transplanting and on crops not severely impacted by whiteflies; use of neonicotinoids as soil drenches at transplanting for best control; avoidance of soil applications of neonicotinoids through drip irrigation systems; avoidance of split applications of soil drenches of neonicotinoids; adherence to scouting recommendations if neonicotinoids are applied foliarly; use of selective rather than broad-spectrum insecticides; and avoidance of insecticide applications on weeds at field perimeters. Growers are encouraged to participate in region-wide management efforts.

# Sub article: Development of a Management Program against Silverleaf Whitefly (SLW), Bemisia argentifolii Bellows & Perring (Hemiptera: Aleyrodidae), using Insecticides

**DOI:** 10.1673/031.008.0487

**Published:** 2008-01

**Authors:** D. R. Seal

**Affiliations:** University of Florida-IFAS, Tropical Research and Education Center, Homestead, FL, USA.

The silverleaf whitefly (SLW), *Bemisia argentifolii* Bellow & Perring, is the most damaging and dangerous insect pest of vegetable production in southern Florida. It is a major pest of tomato, bean, squash, cucumber, watermelon, eggplant, peanut and soybean, and of many ornamental plants. The SLW vectors several extremely damaging plant pathogenic geminiviruses in the United States. In southern Florida, our main concerns are *bean golden mosaic virus* (BGMV) and *tomato yellow leaf curl virus* (TYLCV). Growers use Admire® as a major tool to manage SLW. There is great danger that the current pattern of insecticide use by growers will induce widespread resistance to Imidacloprid and other effective insecticides. In the present studies, efforts have been made to develop on SLW management programs, using various effective chemicals in rotation or in combination. In one study with tomato, treatments evaluated were: 1) Admire® followed by Oberon®; 2) Admire® followed by Oberon® in combination Cohere®; 3) Admire® followed by Knack® in rotation with Applaud®; 4) Admire® followed by Oberon® in combination with Baythroid ® and Cohere®. All treatments significantly reduced SLW eggs and nymphs. In the second study with cucumber, treatments evaluated were: 1) Admire® followed by Oberon®; 2) Admire® followed by Knack® in rotation with Applaud; 3) Admire followed by Oberon in combination with Cohere®. Mean numbers of SLW adults on treated plants did not differ from nontreated control plants. However, all treatments significantly reduced SLW eggs and nymphs on cucumber, compared with the nontreated control. In the third study, various Neem-based products were evaluated for control of SLW in cucumber. These products showed some reduction in SLW numbers at various developmental stages, but in an inconsistent pattern, compared with the nontreated control. This information bears great importance in managing SLW with various insecticides in rotation or in combination. This practice may help to delay development of resistance in SLW against Admire and other effective insecticides.

# Sub article: A knottin-like putative antimicrobial gene family in the whitefly Bemisia tabaci biotype B: Cloning and transcript regulation

**DOI:** 10.1673/031.008.0488

**Published:** 2008-01

**Authors:** Robert G. Shatters, C. L. McKenzie, L. M. Boykin, Shirley Gazit, X. Sinisterra, A. A. Weathersbee, J. K. Brown, H. Czosnek

**Affiliations:** ^1^ Subtropical Insects Research Unit, U. S. Horticultural Research Laboratory, ARS, USDA, Fort Pierce, FL USA.; ^2^ The Robert H. Smith Institute for Plant Science and Genetics in Agriculture, The Hebrew University of Jerusalem, Rehovot, Israel; ^3^ IRREC, IFAS, University of Florida, Fort Pierce, FL USA; ^4^ Department of Plant Sciences, The University of Arizona, Tucson, AZ, USA

The whitefly, *Bemisia tabaci*, is a worldwide plant pest and the vector of agriculturally important plant pathogenic begomoviruses. Of two vectored begomoviruses, *Tomato yellow leaf curl virus* (TYLCV) is transcriptionally active in *B. tabaci* and *Tomato mottle virus* (ToMoV) is not. Comparative analyses of 7093 EST sequences from cDNA libraries from either aviruliferous or ToMoV or TYLCV viruliferous whiteflies revealed four clones of a knottin-like gene family (*btk-1, -2, -3,* and *-4*). Two of these, *btk-1* and *btk-3*, were more abundant in cDNA libraries from viruliferous whiteflies. Time course, virus uptake/acquisition experiments showed physical manipulation of whiteflies induced a transient increase in *btk-1* and *btk-3* transcripts that was influenced by the presence of ToMoV. The Btk proteins range from 59 to 65 amino acids, and contain a secretory signal sequence and a conserved spatial representation (in peptide sequence and predicted 3-D protein-folding model) of six cysteine residues. These characteristics were identical to those for plant and insect knottin-family antifungal/toxin proteins. Begomovirus ingestion and physical stress influence on transcript abundance and sequence similarities with other knottin proteins suggest that btk protein family belongs to the knottin antimicrobial family and are part of the insect innate immune system.

# Sub article: Population genetics of Bemisia tabaci Biotypes B and Q from the Mediterranean and the U.S. inferred using microsatellite markers

**DOI:** 10.1673/031.008.0489

**Published:** 2008-01

**Authors:** Robert G. Shatters, L. M. Boykin, R. A. Bagnall, R. C. Resell, D. R. Frohlich, C. L. McKenzie

**Affiliations:** ^1^ Subtropical Insects Research Unit, U. S. Horticultural Research Laboratory, ARS, USDA, Fort Pierce, FL, USA.; ^2^ Biology Department, University of St. Thomas, Houston, TX, USA

*Bemisia tabaci* is composed of several biotypes, some of which appear to have pre and/or post zygotic barriers to hybridization. Numerous studies have been performed to determine the geographical distribution of different biotypes, but little is know about the gene flow within and among biotypes. Recent research on microsatellite markers associated with different biotypes in Australia and South East Asia indicates limited gene flow among populations. Using the same microsatellite markers, we evaluated gene flow among and within B and Q biotype populations in the Mediterranean region and in the United States. Our data indicate that despite the recent invasion of the Q biotype into the U.S., it has much greater genetic diversity than the B biotype *B. tabaci*. Furthermore, Structure analysis of the Biotype Q microsatellite data indicates that movement of the Q biotype into the U.S. is the result of more than a single introduction. Observed differences in specific markers among the B and Q biotypes does not support recent gene flow between these two biotypes. This data corroborates the mitochondrial COI sequence comparisons performed from populations throughout the U.S.

# Sub article: Wild Germplasm: Plant Resistance for Watermelon

**DOI:** 10.1673/031.008.0490

**Published:** 2008-01

**Authors:** Alvin Simmons, Amnon Levi, Angela Davis, Ling Kai-shu, Rolando Lopez, D. Michael Jackson, Kousik Chandrasekar, B. Merle Shepard, Judy Thies

**Affiliations:** ^1^ USDA, ARS, U.S. Vegetable Laboratory, Charleston, SC, USA.; ^2^ Coastal Research and Education Center, Clemson, University, Charleston, SC, USA

Watermelon (*Citrullus lanatus* var. *lanatus*) is an important worldwide horticultural crop. In the U.S., the largest production areas are Texas, Florida, California, Georgia, Indiana, and Arizona. Pests and diseases are problematic in the production of this crop. Watermelon is an excellent host for some pests including whiteflies in the *Bemisia* complex and spidermites. In addition to direct injury from feeding by *Bemisia*, there is concern about the capacity of this pest to vector diseases. Plant resistance is the most fundamental pest management tool for horticultural crops. However, as verified by the high economic losses, the current level of resistance in commercial watermelon is quite inadequate. We have been conducting research to improve the cultivated watermelon against attack by selected pests and diseases. We have identified germplasm from some wild *Citrullus* sources to have improved resistance to *Bemisia* and other pests, and we are working to incorporate resistance into advanced breeding lines.

# Sub article: Biologically Based Management of Whiteflies in Greenhouse Vegetable Production

**DOI:** 10.1673/031.008.0491

**Published:** 2008-01

**Authors:** P. A. Stansly

**Affiliations:** University of Florida/IFAS, SWFREC, Immokalee, FL USA.

Whiteflies are key pests in vegetable greenhouse production throughout the world. Greenhouse whitefly, *Trialeurodes vaporariorum* (Westwood) is generally the prevalent species in temperate regions while the sweetpotato or silverleaf whitefly *Bemisia tabaci* predominates in warmer parts. Both species damage plants directly through sap removal and indirectly by vectoring virus diseases. Various biotypes of this latter species have been identified, of which biotype “B”, the silverleaf whitefly is dominant in most areas and causes additional damage through physiological disorders such as tomato irregular ripening and squash silverleaf. However, “Q” biotype, originally from the Mediterranean but recently introduced into the US, has developed resistance to many insecticides, including commonly used insect growth regulators and systemic insecticides, and is thus selected for under insecticide-based management regimes. Insecticide resistance along with public pressure has spurred development of alternative management tactics such as insect netting for pest exclusion, and virus-resistant varieties that provide a more compatible environment for biological control. Insect netting must be fine enough to exclude insects, yet allow sufficient ventilation to discourage fungal plant disease. Resistant varieties are often seen as lacking desirable horticultural characteristics and therefore only used when the threat of vector-borne virus disease is acute. Selective insecticides may also be compatible with biological control but are never free of all side effects. The first, effective biological control for whiteflies was *Encarsia formosa*, initially against *T.vaporariorum*, but also used with limited success against *B. tabaci*. This was followed by *Eretmocerus eremicus*, equally effective against both whitefly species, later to be supplanted for control of *B. tabaci* by *E. mundus*. Meanwhile, interest in and use of phytozoophagous mirids in the genera *Macrolophus, Dicyphus* and *Nesiochorus* has been steadily increasing. Excitement has been generated recently by the apparent spectacular success of the predaceous mite *Amblyseius swirskii*. Many challenges remain to integrate use of these species with each other and with biological and chemical control of other greenhouse pests such as spider mites and thrips.

# Sub article: Implementation of Chemical and Biological Protection Compounds of Different Chemical and/or Biological Groups against Whiteflies in IPM Systems

**DOI:** 10.1673/031.008.0492

**Published:** 2008-01

**Authors:** Guido Sterk, M. Van de Veire, K. Put

**Affiliations:** ^1^ Biobest N.V. Belgium.; ^2^ Ghent University, Faculty Agricultural & Applied Biological Sciences Belgium

Several new chemical insecticides against whiteflies have recently reached the market in Europe. The most important ones are flonicamid, pymethrozine, spiromesifen and some neonicotinoids. To determine if they fit with biological control agents and pollinators in IPM systems in protected crops, their side-effects were determined in semi-field trials against several beneficial organisms. The so called physical compounds are another specific group of pesticides. They only kill through their sticking or suffocating mode of action, no active ingredients or no direct effects on the pest are involved. Some of them are very effective against larvae and adults of different species of whiteflies, spider mites, pear sucker and even powdery mildew. They also seem to be rather harmless for most predators, parasitoids and pollinators. However, the main risk here is their possible fytotoxicity for certain varieties of greenhouse crops, fruit trees or ornamentals. Entomopathogenic fungi are used in glasshouses and protected crops in Europe against *Trialeurodes vaporariorum* and *Bemisia tabaci.* Some trials were made in Spain against *Bemisia tabaci* with PreFeRal, based on the fungus *Paecilomyces fumosoroseus* in comparison with *Beauvaria bassiana* with encouraging results. While testing the side-effects of fungicides against *Paecilomyces fumosoroseus* it became clear that some of them were highly effective themselves against whiteflies. This was investigated in detail at the Ghent University, and mancozeb e.g. turned out to show a very good efficacy against eggs and larvae of *Trialeurodes vaporariorum*.

# Sub article: Cotton Whitefly, Bemisia tabaci(Genn.) Degree-Hour Model for Predicting Phenological Development

**DOI:** 10.1673/031.008.0493

**Published:** 2008-01

**Authors:** Bahram Tafaghodinia, Saied Iranmanesh

**Affiliations:** ^1^ Entolomology Department, Agricultural Research Center, Iranian Research Organization for Science and Technology, Tehran, Iran.; ^2^ Entomology Department, Faculty of Agriculture, Tehran University, Karaj, Iran

As part of a pest management analysis and planning program we developed a degree-day based computer simulation model to forecast development periods of immature stages of *Bemisia tabaci* in cotton fields of Tehran state. B. tabaci has been reported from all continents except Antarctica. Over 900 host plants have been recorded for B. tabaci and it reportedly transmits 111 virus species. This insect has been difficult to control because of its resistance to many pesticides, its ability to attack many different plants, its high rate of population growth, and its readiness to disperse from field to field. Because of these characteristics the adequate management of this pest may require an ability to forecast population growth. In this situation, simulation models can be useful because they allow the prediction of *B. tabaci* abundance over a period of time. Our model was designed to imitate the dynamics of the real *B. tabaci* system so as to help in choosing the best time and strategy for management from amongst the whole range of possibilities. Parameter values were obtained from the literature or calculated from cotton field data. All simulations were performed on an IBM compatible computer, using the Stella Ver 7.0 program. Based on these data we developed a phenological model for *B. tabaci* that uses temperature to predict development times of immatures. The inputs to the model are maximum and minimum temperature and latitude of the local area; the outputs are growth and dynamics of population of all stages during night and day. The model has three parts. In the first part, length of day is calculated based on the day number (1 is for first of January) and local latitude. We considered three different periods during a night and day to calculate temperature oscillations. Lower and upper developmental thresholds for eggs to pupa were estimated using linear regression (Lower=10C° and Upper=.32 C°). Length of day and maximum and minimum daily temperature were used to calculate the hourly changes in temperature during the day. Finally the effective degree-hour was calculated using hourly temperature data. In the second part, the output of the first part (effective degree-hour) was used to calculate the transit time of all stages. The degree-hours to complete all stages from egg to pupa are: 1189, 1271, 922, 893, 1084, 2235 C°, respectively. In the third part, we will have the population oscillation of all stages. To verify the model predictions we checked the output by constant transit time. This model simulated phenological patterns that agreed with field records. Thus, the model may be useful for predicting the density and phenology of *B. tabaci*.

# Sub article: Oberon® 2SC: A New Resistant Management Tool for Whitefly Control in Vegetables

**DOI:** 10.1673/031.008.0494

**Published:** 2008-01

**Authors:** Marco Toapanta, David Schuster, Rajinder Mann, Roberta Cordero, Lamar Buckelew, Robert Steffens, Shane Hand, Richard Rudolph, Ralf Nauen

**Affiliations:** ^1^ Bayer CropScience, Brandon, FL., USA.; ^2^ Gulf Coast Research and Education Center, University of Florida, Wimauma, FL., USA; ^3^ Bayer CropScience, Research Triangle Park, NC., USA; ^4^ Bayer CropScience, Monheim, Germany

Oberon® contains a novel insecticidal and acaricidal active ingredient, spiromesifen, from the new chemical class of spirocyclic tetronic acids, generally known as keto-enols. This new compound is effective against all developmental stages of whiteflies (*Bemisia* spp and *Trialeurodes* spp), mites, and other pests, through a new mode of action (MOA), inhibition of lipid biosynthesis. It is listed in the IRAC-International Mode-of-Action classification as, MOA Group 23, a lipid biosynthesis inhibitor, together with spirodiclofen. In field tests conducted between 2000 and 2005 in Florida, Oberon, as a 240 g AI/liter suspension concentrate (SC) formulation, applied in different vegetable cropping systems at rates ranging from 100 to 150 g AI/ha, provided significantly greater control of *B. tabaci* biotype B nymphs than conventional insecticides. In addition, foliar applications of Oberon resulted in longer residual control than commercial insecticides including the juvenile hormone mimic, pyriproxyfen and the chitin synthesis inhibitor, buprofezin. Baseline susceptibility tests conducted in 2005 with 2nd instars of *B. tabaci* biotype B collected from tomato-growing areas in west-central Florida showed LC50 values ranging from 0.27 to 0.85 mg liter-1, with the former value corresponding to the laboratory susceptible colony. The values found here are within a range of values obtained elsewhere for *B. tabaci* biotype B and Q populations including neonicotinoid and pyriproxyfen resistant strains. Monitoring tests conducted in 2006 with 2nd instars of *B. tabaci* biotype B collected from tomato-growing areas in west-central and southwest Florida, which received Oberon applications during the growing season, resulted in LC50 values below the values obtained in the baseline tests, thus confirming full susceptibility of *B. tabaci* to field applications of Oberon. Moreover, Oberon showed no cross-resistance to other commercial insecticides applied to control whiteflies, thus representing a new valuable tool in whitefly resistance management, in combination or rotation with other insecticides, such as Admire Pro Systemic Protectant, a MOA Group 4A.

# Sub article: Integrated Management Approach to Bemisia tabaci control in Agricultural Systems

**DOI:** 10.1673/031.008.0495

**Published:** 2008-01

**Authors:** Nick C. Toscano

**Affiliations:** Department of Entomology, University of California, Riverside, CA, USA.

The importance of the agricultural system in creating high densities of *Bemisia tabaci* populations can not be underestimated. The types and sequence of crops grown in the Imperial and southern San Joaquin valleys provide a constant source of food for whitefly populations. In these valleys, alfalfa, cotton, cole crops and melons are grown in vast acreages and in sequences that serve as major sources of whiteflies. Due to the overlapping harvesting of a number of crop hosts, perpetually high numbers of whiteflies disperse from one crop host to another. Whitefly movement from May through November resulted in a heavy reliance upon insecticides, which may continue to be necessary for crop protection. Management of building whitefly populations led to creation of several regional programs in Arizona and California. These programs varied in their approaches, but resulted in reduced and created manageable whitefly populations in their respective agricultural systems. Management and reduction of *B. tabaci* populations in California resulted from various changes in the agricultural system. These include the use of the neonicotinoid, imidacloprid; use of insect growth regulators, buprofezin and pyriproxyfen; implementation of management decisions; monitoring for insecticide resistance; short season and reduction of cotton acreage; shortening of alfalfa harvesting cycles, and cropping pattern changes. In general, all the aforementioned factors impact *B. tabaci* population densities, with some are being more important than others.

# Sub article: Biotype monitoring and genetic relationships of Bemisia tabaci in Greece: Mitochondrial DNA and microsatellite polymorphism

**DOI:** 10.1673/031.008.0496

**Published:** 2008-01

**Authors:** A. Tsagkarakou, C. S. Tsigenopoulos, K. Gorman, J. B. Kristoffersen, J. Lagnel, D. Bedford

**Affiliations:** ^1^ National Agricultural Research Foundation, Plant Protection Institute, Heraklion, Greece; ^2^ Institute of Marine Biology and Genetics, Hellenic Centre for Marine Research, Heraklion, Greece; ^3^ Rothamsted Research, Harpenden, Hertfordshire, United Kingdom.; ^4^ John Innes Centre, Colney Lane, Norwich, Norfolk, United Kingdom

We studied the biotype identity and the genetic relationships of *Bemisia tabaci* from different localities in Greece (mainland Greece and the island of Crete) between 2002 and 2006. Two different approaches were used, sequencing of the mitochondrial cytochrome oxidase I (mtCOI) gene and genotyping using microsatellite markers. Analyses of mtCOI sequences revealed a high homogeneity between samples; only five polymorphic sites were detected in sequences of an 879 bp mtCOI fragment from 16 Greek insects. Comparison of the mtCOI sequences from Greek *B. tabaci* with sequences from reference biotypes identified only the Q biotype within Greece. Greek whiteflies clustered with other Q biotype *B. tabaci* from Spain, Portugal, and Morocco, and were loosely separated from a group that included Q biotype sequences from more eastern Mediterranean countries (Turkey, Cyprus and Israel). Based on sequence differences, two molecular diagnostic tests for discriminating between Q and B and non Q / B biotypes were developed involving amplification of biotype specific mtCOI fragments or digestion of mtCOI PCR fragment with *Alu*I*.* These tools showed the absence of the B biotype and presence of the Q biotype in all 40 Greek samples examined. In contrast, a high genetic differentiation was detected between Q biotype samples from Crete using six microsatellite markers. Gene flow appeared low even between populations separated by just a few kilometres (*F*ST estimates ranging from 0.437 to 0.025; *P* < 0.01). This differentiation was supported by Bayesian analyses showing that individuals clustered into at least two groups based on microsatellite data.

# Sub article: Characterization and distribution of the biotypes of the whitefly, Bemisia tabaci, in Japan by mitochondrial cytochrome oxidase I sequence analysis

**DOI:** 10.1673/031.008.0497

**Published:** 2008-01

**Authors:** Shigenori Ueda, Toshio Kitamura, Honda Ken-ichiro, Kenkichi Kanmiya

**Affiliations:** ^1^ National Agricultural Research Center for Kyushu Okinawa Region, National Agriculture and Food Research Organization, Kumamoto, Japan.; ^2^ National Institute of Vegetable and Tea Science, National Agriculture and Food Research Organization, Mie, Japan; ^3^ Kurume University, School of Medicine, Fukuoka, Japan

The sweetpotato whitefly, *Bemisia tabaci* (Gennadius), has been considered a major pest insect of cultivated crops worldwide. The first report of the introduction and establishment of the B biotype in Japan was in approximately 1989 associating with imported poinsettia plants. Since then, the B biotype had been quickly dispersed and caused irregular ripening of tomato fruit in greenhouses, silverleaf symptoms in pumpkin, and symptomatic soybean plants in multiple locations in Japan. Since 1996, *Tomato yellow leaf curl virus* (TYLCV) has been introduced and dispersed widely in West Japan. To prevent TYLCV transmission by the vector whitefly, a large amount of insecticides have been used with some physical controls such as screens for avoiding severe yield loss in tomato greenhouses in Japan. The Q biotype was first reported in February 2005 by mitochondrial cytochrome oxidase I (mtCOI) sequence analysis. These whiteflies were collected in 2004 in Hiroshima, Kagoshima and Kumamoto prefectures, respectively. Until July 2005, the Q biotype has been found from various vegetable and ornamental crops in 30 prefectures in Japan. In addition, in 2006, the B2 biotype was first identified in Gunma Prefecture in Kanto district. It was not clear on actual routes of these invasive populations. One indigenous genetic type of *B. tabaci* was identified in the Japanese islands by mtCOI analysis. In Japan, the local population of *B. tabaci, B. lonicerae* Takahashi was first described in 1957. This population normally infested on weed honeysuckle plants, *Lonicera japonica*. Before 1990s, it was supposed to be a vector insect, which occasionally transmits indigenous begomoviruses from weed to tomato or tobacco plants. Cross experiments between the local population and B biotype were performed, but it was failed because of differences of acoustic vibratory sounds (Ohtaishi & Okada, 1996; Kanmiya, 1998). Since 2004, distinct individuals have been collected from *Lonicera japonica, Lycopersicon esculentum* and *Perilla frutescens*, which formed a distinct group in the Southeast Asia/Far East clade by phylogenetic analyses. The genetic type, named JpL (Japan, *Lonicera japonica*) was detected from Kanto district (in Honshu) through Kyushu. This biotype should be considered an indigenous biotype in the Japanese islands because individuals were mainly collected from *Lonicera japonica*.

# Sub article: Successes and Challenges of Using Biological Control against Bemisia tabaci A Producer's View-point

**DOI:** 10.1673/031.008.0498

**Published:** 2008-01

**Authors:** Jan Vermeulen, Paco Lozano Rubio

**Affiliations:** Biobest NV, Belgium.

The success and challenges of biological *Bemisia tabaci* control in protected crops will be considered from a producer's view-point. Today already a set of successful natural enemies against *B. tabaci* exists: the parasitoids *Eretmocerus eremicus* and *E. mundus,* the predatory mite *Amblyseius swirskii,* the predatory bugs *Macrolophus caliginosus* and *Nesidiocoris tenuis.* The fungi *Paecylomyces fumosoroseus* and *Verticillium lecanii* and other microorganisms are available. Promising new natural enemies such as *Encarsia hispida* are being investigated. More new products and better understanding and use of the existing tools have to be developed. This requires technical knowledge, understanding of the market and good business sense. We also have to cope with regulatory requirements such as import regulations and ecological concerns. Recently a new issue, patenting of the use and production of biological agents has come up.

# Sub article: Resistance to Silverleaf Whitefly, Bemisia argentifolii (Hemiptera: Aleyrodidae) in Gossypium thurberi, a Wild Cotton Species

**DOI:** 10.1673/031.008.0499

**Published:** 2008-01

**Authors:** G. P. Walker, E. T. Natwick

**Affiliations:** ^1^ Department of Entomology, University of California, Riverside, CA, USA; ^2^ Division of Agriculture and Natural Resources, University of California Cooperative Extension, Holtville, CA, USA.

*Gossypium thurberi* Todaro is a wild cotton species native to Mexico and parts of the southwestern United States. Four years of field studies in California's Imperial Valley revealed consistent high levels of resistance in *G*. *thurberi* against silverleaf whitefly, *Bemisia argentifolii* Bellows & Perring, an important worldwide pest of cotton. Naturally developing field infestations in plots of *G. thurberi* were significantly lower than in plots of the *G. hirsutum* L. commercial cotton cultivars, DP 5415, Siokra L23, and Stoneville 474. *G. thurberi* has two morphological traits that, in past research, have been associated with lower levels of whitefly susceptibility, smooth-leaf and okra-leaf; however, the levels of resistance observed in *G. thurberi* were significantly greater than the cotton cultivar DP 5415, which is a smooth-leaf cotton, and Siokra L23 which, like *G. thurberi*, has both smooth-leaf and okra-leaf traits. Therefore, the high level of resistance in *G. thurberi* seems to be due to factors above and beyond smooth-leaf and okra-leaf. In contrast to the clear results on naturally developing field infestations, experiments comparing nymphal survival among *G. thurberi* and commercial cotton cultivars did not detect antibiosis and both choice and no-choice oviposition experiments did not detect antixenosis. Immature survival (first instar through adult) was actually slightly higher on *G. thurberi* than on DP 5415 in the greenhouse test. This test was conducted on very young plants, so it seemed plausible that the unexpected results were due to resistance not being expressed in young plants and/or in greenhouse conditions. However, the next two tests in the field on mature plants also detected little or no resistance in *G. thurberi*. Likewise choice and no-choice oviposition tests in the greenhouse and in the field did not detect any difference in whitefly oviposition rate between *G. thurberi* and DP 5415, Table 1. At this point, while the data conclusively demonstrate a high level of whitefly resistance in *G. thurberi*, the mechanisms of the resistance remains an enigma. Despite the failure of the oviposition tests to demonstrate non-preference, we suspect that non-preference is the most likely candidate for the mechanism of resistance. The reason for this suspicion is that each year of the field plot tests, the number of adults and eggs early in the season, which would be the initial colonization, were much less on *G. thurberi* than on the other cotton entries. In our choice and no-choice oviposition experiments in this study, the whiteflies were in a confined space which may have interfered with their normal host selection behavior. We plan additional oviposition tests using less confining conditions to investigate this possibility further.

# Sub article: Effects of heat shock on survival and fecundity of two whitefly species, Trialeurodes vaporariorum and Bemisia tabaci B-biotype

**DOI:** 10.1673/031.008.04a1

**Published:** 2008-01

**Authors:** Wan Fang-Hao, Ming Xie, Cui Xu-Hong

**Affiliations:** ^1^ State Key Laboratory for Biology of Plant Diseases and Insect Pests, Institute of Plant Protection, Chinese Academy of Agricultural Sciences, Beijing, China.; ^2^ Center for Management of Invasive Alien Species, Ministry of Agriculture, Beijing, China

The effects of heat shock on survival and fecundity of two whitefly species, *Trialeurodes vaporariorum* (Westwood) and *Bemisia tabaci* (Gennadius) B-biotype, were examined after treatment for 1 h at five temperatures: 37, 39, 41, 43 and 45°C, under laboratory conditions. The objectives of the study were to evaluate the responses of both whitefly species to temperature stress and to understand why they might coexist in different areas with different population dynamics in China. The results showed that the heat tolerance of egg, red-eyed nymph and adult *B. tabaci* B-biotype was higher than that of *T. vaporariorum*. For both whitefly species, the response of adults to heat shock was related to sex; female adults were more heat tolerant than adult males. Heat shock had no detrimental effect on the pre-oviposition period of both whitefly species; female adults began to lay eggs in 2 d after a heat shock. However, the fecundity of both whitefly species was affected. When both sexes of *B. tabaci* B-biotype adults were heat shocked, the fecundity per female varied with the degree of heat shock, but there were no significant differences between heat-shocked and control female individuals. In the case of *T. vaporariorum,* the number of eggs produced per female was strongly affected by heat shock, significantly decreasing with the increasing heat shock temperature. Parental heat shock had substantial and lasting effects on egg hatching ability and the sex ratio of offspring of both whitefly species. Following the heat shock, ranging from 37 to 45°C, the proportion of hatched *B. tabaci* B-biotype eggs was reduced by 10–40% compared to the control, but no *T. Vaporariorum* eggs survived when parental adults were exposed at 43°C for 1 h. The average sex ratio (expressed in % females) of *B. tabaci* B-biotype was about 55% after heat shock at 26, 37 and 39°C, respectively. When the temperature was above 43°C, the ratio of females decreased and that of males increased to over 60%. For *T. vaporariorum*, the mean sex ratio was about 54% when the heat shock temperature varied from 37 to 41°C, and there was no significant correlation between sex ratio and degree of heat shock. Our results indicated that *T. vaporariorum* was more sensitive to higher temperature than *B. tabaci* B-biotype. The different thermal adaptation between *B. tabaci* B-biotype and *T. vaporariorum* could be an important determinant in population dynamics and distribution of both whitefly species in China.

# Sub article: Dynamics of Digestive Enzymes of Bemisia tabaci (Gennadius) B-biotype and Trialeurodes vaporariorum (Westwood) During Plant Species Switching

**DOI:** 10.1673/031.008.04a2

**Published:** 2008-01

**Authors:** Wan Fang-Hao, Gui-Fen Zhang, Fang Lei

**Affiliations:** ^1^ State Key Laboratory for Biology of Plant Diseases and Insect Pests, Institute of Plant Protection, Chinese Academy of Agricultural Sciences, Beijing, China.; ^2^ Center for Management of Invasive Alien Species, Ministry of Agriculture, Beijing, China

*Bemisia tabaci* (Gennadius) B-biotype is a key pest of greenhouse vegetables, ornamental crops and cotton in many regions of the world. It is an alien invasive species in China. The range of host plant species colonized by *B. tabaci* B-biotype has been increasing with its invasive spread to new areas. Furthermore, in north China, competition between *B. tabaci* B-biotype and its co-existing, closely related species *Trialeurodes vaporariorum* (Westwood), has been observed. Biochemical techniques were used to understand potential reasons why host plant species of *B. tabaci* B-biotype are increasing and to clarify its competition with *T. vaporariorum. B. tabaci* B-biotype and *T. Vaporariorum* were reared on tomato for more than 5 generations, and activities of the digestive enzymes: trehalase, amylase, and proteinase were evaluated when both whitefly species were switched to cabbage, maize and cotton plants. Results showed that the trehalose contents of *B. tabaci* B-biotype and *T. Vaporariorum* decreased equally when the insects were switched from tomato to the three tested plant species, especially cotton. However, when switched to non-host maize, the recovery ability and stability of *B. tabaci* B-biotype was greater than that of *T. vaporariorum*. The dynamics of trehalase activity of *B. tabaci* B-biotype were not different following the host switch to cotton, cabbage or maize, but patterns differed for *T. vaporariorum*. The dynamics of trehalase activity of *B. tabaci* B-biotype and *T. vaporariorum* on non-host maize plants were not significantly different; however, the recovery ability and stability of *B. tabaci* B-biotype were greater than those of *T. vaporariorum*. The dynamics of amylase of *B. tabaci* B-biotype were more regular than those of *T. vaporariorum*. The dynamics of protein contents of both whitefly species were similar following the host switch to cotton. The protein content of *B. tabaci* B-biotype increased with a prolonged feeding period on cabbage, however, that of *T. vaporariorum* decreased. The dynamics of the protein contents of both whitefly species on maize were similar to those on cabbage, but they were much subtler. The proteinase activity was not significantly different when *B. tabaci* B-biotype was switched to the three tested host plants, but it was significantly reduced when *T. vaporariorum* was switched to cotton or maize. Significant differences had been observed between the two whitefly species when feeding on the three tested plant species for 2h. The dynamics of proteinase activity of *B. tabaci* B-biotype were modeled as activated - inhibited when switched to cotton and cabbage, but as inhibited - activated when switched to maize. However, the dynamics of proteinase activity of *T. vaporariorum* were similar on the three tested plant species. Our results indicated that the adaptability of *B. tabaci* B-biotype to plant species switches was greater than that of *T. vaporariorum*.

# Sub article: Host Plant Infection by a Plant Pathogen Changes Parasitoid Host-Searching Behavior: A Case Study Using Whiteflies and Encarsia Formosa

**DOI:** 10.1673/031.008.04a3

**Published:** 2008-01

**Authors:** Liande Wang, Stefan Vidal

**Affiliations:** ^1^ Key Laboratory of Biopesticide and Chemical Biology, MOE. Fujian Agriculture & Forestry University, Fuzhou, P.R. China.; ^2^ Department of Crop Science, Entomological Section, Georg-August-University, Goettingen, Germany

Plants frequently suffer from either simultaneous or sequential infestations or attack by plant pathogens and/or insect herbivores. When damaged, plants release volatile compounds (VCs), which are used by parasitoids to locate their herbivorous hosts. However, few studies so far have investigated the influence of simultaneous colonization of plants by plant pathogens and insect herbivores on the efficacy of parasitoids to locate their hosts. We conducted a series of laboratory and greenhouse experiments using tomato plants, the powdery mildew *Oidium neolycopersici*, whiteflies (*Trialeurodes vaporariorum*) and the parasitoid *Encarsia formosa*, respectively, to test the impact of diseased host plants on herbivore-parasitoid-interactions. *E. formosa* females were significantly less attracted to powdery mildew-infected tomato plants (PMP) as compared to healthy plants (HP); thus parasitism rates of whiteflies (WF) were significantly lower on PMP as compared to HP (17.5:41.5 %). These differences disappeared when *E. formosa* females were forced to parasitize whiteflies on either PMP or HP. The performance of the parasitoid was not significantly influenced when developing on whiteflies feeding on powdery mildew infected plants. In olfactometer bioassays, either naïve or experienced parasitoids significantly preferred healthy plants colonized by whiteflies (HPWF) as compared to powdery mildew infected plants colonized by whitefly (PMPWF) (30.0:10.0% for naïve parasitoids; 41.4:15.2% for experienced parasitoids). In wind tunnel bioassays, volatiles emanating from whiteflies significantly increased upwind oriented flight and landing in *E. formosa* targets as compared to any other treatments. Volatiles from HP, PMP, PMPWF and WF, respectively were collected and analyzed by GC and GC-MS, revealing that both qualities and quantities of volatile compounds differed to a large extend. Plants significantly increased volatile emission after being attacked by whiteflies (275.3 mol.hr -1) or inoculated by powdery mildew (267.58 mol.hr -1). However, plants significantly reduced volatile emissions in PMPWF co-existing systems (80.58 mol.hr -1). Our results demonstrate that volatiles released by either herbivore damaged plants, pathogen diseased plants or combinations of these agents, respectively, may influence the foraging behavior of the parasitoids while searching for their hosts. The negative impact of diseased host plants on the parasitoids host-searching behavior resulted in reduced efficacies of parasitoids. This in turn may impact the outcome of biological control systems, especially under greenhouse conditions.

# Sub article: Response by a Whitefly Predator, Delphastus catalinae to Toxins from Verticillium lecanii and Mass Rearing Techniques for the Ladybeetle

**DOI:** 10.1673/031.008.04a4

**Published:** 2008-01

**Authors:** Liande Wang, Jian Huang, Hongwei Luo

**Affiliations:** ^1^ Key Laboratory of Biopesticide and Chemical Biology, MOE. Fujian Agriculture & Forestry University, Fuzhou, P.R. China.; ^2^ Department of Crop Science, Entomological Section, Georg-August-University, Goettingen, Germany

*Delphastus catalinae* Horn (Coleopetra: Coccinellidae) is one of the most important predators attacking whiteflies and was imported from Britain to control the invasive pest *Bemisia tabaci* in southern China. Response of the beetles to metabolite toxins from the entomopathogenic fungus, *Verticillium lecanii* was studied in the laboratory and in field experiments to evaluate compatibility of the two biological control agents. Larval beetles showed slight sensitivity, while adult beetles were not susceptible to the metabolite toxins from the fungus. Consumption and foraging capacity were significantly impaired, especially for second-instar beetles which took twice as long as control beetles to consume whitefly eggs after exposure to toxins. However, no significant effect on fecundity and longevity of the beetle was observed after exposure to the toxin at field rate. The data suggest that spraying of *V. lecanii* or its toxins should be avoided in the field when immature stages of *D. catalinae* are present. Six experimental diets were formulated and tested for rearing *D. catalina*. A liquid formulation confined in two layers of Parafilm proved to be satisfactory for rearing this coccinellid and compatible with its feeding habits. The basic ingredients of this diet were chicken egg, pollen, sugar, vitamin B and honeybee drone pupal homogenate. Olive oil and honeydew were added as feeding stimulants. Adult beetles reared on this formulation lived more than 30 d. Rearing alternately on artificial diet and on natural prey could resolve the “bottle neck” created by decreasing fecundity of colonies reared on artificial diet on the one hand and short supply of the natural prey food for the mass rearing system on the other.

# Sub article: Vector Specificity of Criniviruses in Tomato and Virus Competitiveness during Mixed Infection

**DOI:** 10.1673/031.008.04a5

**Published:** 2008-01

**Authors:** W. M. Wintermantel

**Affiliations:** USDA-ARS, Salinas, CA, USA.

*Tomato chlorosis virus* (ToCV), family *Closteroviridae,* genus *Crinivirus*, causes interveinal chlorosis, leaf brittleness and limited necrotic flecking or leaf bronzing on tomato leaves. ToCV can cause a decline in plant vigor and reduce fruit yield; it is emerging as a serious production problem for field and greenhouse tomato growers, and has been increasing in prevalence in many parts of the world. ToCV has a moderately wide host range, infecting 24 host plant species in seven families. The virus is unique among known whitefly-transmitted viruses, due to its ability to be transmitted by four whitefly vectors from two genera. Studies demonstrated that transmission efficiency and virus persistence in the vector varies significantly among the different whitefly vectors. *Trialeurodes abutilonea* and *Bemisia tabaci* biotype B are highly efficient vectors of ToCV. *B. tabaci* biotype A and *T. vaporariorum* are less efficient vectors, but are fully capable of transmission. ToCV persists for up to *5* days in *T. abutilonea*, 2 days in *B. tabaci* biotype B, and only 1 day in *B. tabaci* biotype A and *T. Vaporariorum*. ToCV is closely related to another crinivirus, *Tomato infectious chlorosis crinivirus* (TICV). TICV and ToCV produce identical symptoms on tomato, but TICV differs from ToCV in that it is transmitted exclusively by *T. vaporariorum*. Geographical distribution of TICV and ToCV depends on distribution of the whitefly vectors. In some parts of the world both viruses have been found together in tomato, indicating that infection by one crinivirus does not prevent infection by a second. Crinivirus epidemiology is impacted not only by vector-movement of viruses, but also by factors such as virus competitiveness in host plants. It is likely that competitiveness of each virus varies among different host plant species, and may be influenced by factors such as plant age or which virus became established first. Alternatively, co-infection may increase the potential for genetic recombination or pseudo-recombination between related crinivirus species, and may contribute to selection pressure leading to emergence of new strains or species with altered host range, symptomatology or vector specificity. In order to elucidate the effects of co-infection on crinivirus vector specificity and accumulation, we established *Physalis wrightii* and *Nicotiana benthamiana* source plants, containing either TICV alone, ToCV alone, or both viruses together. *T. Vaporariorum* and *T. abutilonea* were allowed to feed separately on all virus sources, as well as virus-free plants for 24 hours, then were transferred to young host plants. Symptomatic plants were tested by northern hybridization and quantitative RT-PCR, and indicated some host-specific differences in accumulation by TICV and ToCV. Interestingly, transmission of TICV from mixed infections by the non-vector, *T. abutilonea* was confirmed in two of 56 plants.

# Sub article: Responses of Two Whitefly Species, Trialeurodes vaporariorum (Westwood) and Bemisia tabaci (Gennadius) B-biotype, to Low Temperatures

**DOI:** 10.1673/031.008.04a6

**Published:** 2008-01

**Authors:** Ming Xie, Chen Yan-Hua, Wan Fang-Hao

**Affiliations:** ^1^ State Key Laboratory for Biology of Plant Diseases and Insect Pests, Institute of Plant Protection, Chinese Academy of Agricultural Sciences, Beijing, China.; ^2^ Center for Management of Invasive Alien Species, Ministry of Agriculture, Beijing, China

Two alien whitefly species, *Trialeurodes vaporariorum* (Westwood) and *Bemisia tabaci* (Gennadius) B-biotype, coexist in different geographic areas in China. The population density of *B. tabaci* B-biotype has been growing since the mid 1990s. The responses of both whitefly species to low temperatures were evaluated to clarify the interspecific differentiation in adaptation. The results showed that the cold-tolerant ability of all developmental stages of *T. vaporariorum* was much greater than that of *B. tabaci* B-biotype when they were exposed at 2°C for a series of time durations, 1–12d. Acclimation could significantly influenced cold tolerance of two whitefly species. When acclimated at 5°C for 48 h, the survival of *T. vaporariorum* was 78% at -8°C for 10 h, much higher than that of *B. tabaci* B -biotype. The optimal temperature range for *T. vaporariorum* development was lower than that for *B. tabaci* B-biotype. The developmental duration from egg to adult *B. tabaci* B-biotype was 1.5 times longer than *T. vaporariorum* when they were reared at 18° and 15°C, respectively. However, the development of *B. tabaci* B-biotype was faster than *T. vaporariorum* when the temperature was above 24°C. Our results indicated that the tolerant ability of *T. vaporariorum* to lower temperature was greater than *B. tabaci* B-biotype, which might facilitate the development of *T. vaporariorum* populations in the cool season, thus affecting the distributions of the two whitefly species.

# Sub article: Research on Bemisia and Other Whiteflies in China

**DOI:** 10.1673/031.008.04a7

**Published:** 2008-01

**Authors:** Yan Fengming

**Affiliations:** College of Life Sciences, Peking University, Beijing, China.

About 200 whitefly species have been recorded in China, among which only several species become pests in agricultural, horticultural and ornamental crops, such as *Aleurocanthus spiniferus* (Quaintance), *Trialeurodes vaporariorum* (Westwood), *Dialeurodes citri* (Ashmead), and *Bemisia tabaci* (Gennadius), with *A. spiniferus* and *D. citri* only damaging orange, rice and other crops in south China. Whiteflies were not a serious problem in north part of China until *T. Vaporariorum* broke out in greenhouse crops in 1980's when China started to develop greenhouse vegetable production on a large scale. *B. tabaci* was first recorded in China in 1940's, and only occurred occasionally in some areas. However, biotype “B” has been an extremely harmful crop pest in both greenhouses and open fields since mid-1990's. This pest was identified with mainly molecular approaches as the exotic *B. tabaci* biotype ‘B’ probably invading on imported flowers or other imported plants. *B. tabaci* B biotype has caused outbreaks in over 20 provinces all over the country, resulting in great losses annually to agricultural and horticultural crops, while other biotypes of *B. tabaci* are distributed in very limited areas. Research on distribution, biology, host plant range, as well as genetic variations, have been conducted in many institutions supported by funds from national and local governments. Due to its reproductive potential, adaptation, tolerance to environmental conditions, and resistance to insecticides, biotype “B” is very difficult to control. Efforts are being made to manage biotype “B” sufficiently, by cultural, physical and chemical methods, and biological control using entomopathogenic microbes, parasitoids and predators, as well as quarantine measures. Since the planting of transgenic cotton on a large scale in China, non-target pests, such as mites, hemipterans, aphids, and *B. tabaci*, have resurged, especially in Bt cotton fields compared to conventional cotton fields. Concerns have arisen towards the ecological consequences of Bt cotton release on a large scale. Studies of feeding behavior of *B. tabaci* biotype “B” using recordings made with an electrical penetration graph (EPG) in our laboratory have shown that *B. tabaci* B biotype behaves differently on Bt cotton and conventional cotton.

# Sub article: Intraguild Interactions between Bemisia tabaci Predator, Delphastus catalinae, and Parasitoid, Encarsia sophia, and their Impacts on Whitefly Suppression

**DOI:** 10.1673/031.008.04a8

**Published:** 2008-01

**Authors:** Zang Lian-Sheng, Liu Tong-Xian

**Affiliations:** Vegetable IPM Laboratory, Texas A&M Agricultural Experiment Station, Weslaco, TX USA.

The intraguild interactions between two natural enemies of *Bemisia tabaci* (Gennadius), a predator, *Delphastus catalinae* (Horn), and a parasitoid, *Encarsia sophia* (Girault and Dodd), associated with predation, parasitization, host feeding, and suppression of *B. tabaci* were determined under laboratory and greenhouse conditions. In no-choice tests, the 2nd instar *D. catalinae* most prefer unparasitized 4th-instar *B. tabaci*, followed by the whitefly nymphs with 2nd and 3rd instars of *E. sophia*, and the nymphs with *E. sophia* pupae were the most non-preferred prey. The 3rd and 4th instars and adults of *D. catalinae* prey fewer whitefly nymphs with *E. sophia* pupae than other parasitized and unparasitized whitefly nymphs. In choice tests, the 2nd instar *D. catalinae* prey more unparasitized whitefly nymphs and the nymphs with 2nd instar *E. sophia* than the nymphs with 3rd instar and pupae of *E. sophia*. The 3rd and 4th instar *D. catalinae* prey fewer whitefly nymphs with *E. sophia* pupae than other parasitized and unparasitized nymphs. *D. catalinae* adults did not show any preference among the parasitized and unparasitized whitefly nymphs. In a greenhouse experiment with two treatments, *E. sophia* alone and *E. sophia* plus *D. catalinae*, more *B. tabaci* nymphs were killed (parasitism, host feeding, host mutilation and natural mortality) by *E. sophia* than by *E. sophia* with addition of *D. catalinae*, implying that parasitization and host feeding activities of *E. sophia* were reduced with the presence of *D. catalinae*. The rates of whitefly population increase were significantly lower in the five treatments with various numbers of parasitoids and lady beetles after release of *B. tabaci* adults (15 females and 15 males) on to the plants in a cage over a period of four weeks than the plants without natural enemies. Generally, the plants with release of 6 and 12 *D. catalinae* adults and 20 *E. sophia* adults and 6 *D. catalinae* adults had lower whitefly populations than thetreatments of 20 and 40 *E. sophia* adults. Similarly, the percentages of whiteflies killed by both *D. catalinae* and *E. sophia* were greater in the treatments with *D. catalinae* than those without. These results indicate that *D. catalinae* did not avoid feeding on *B. tabaci* nymphs with larval stages of *E. sophia,* and the activities of *E. sophia* to control whiteflies were greatly offset in the presence of *D. catalinae*.

# Sub article: Status of Eretmocerus (Hymenoptera: Aphelinidae) Systematics

**DOI:** 10.1673/031.008.04a9

**Published:** 2008-01

**Authors:** Gregory Zolnerowich

**Affiliations:** Department of Entomology, Kansas State University, Manhattan, KS, USA.

An overview of the current systematics of the genus *Eretmocerus* is presented. There are currently 65 described species of *Eretmocerus*, with 16 of those recorded from *Bemisia* (*tabaci* group). For some *Eretmocerus* species names associated with *Bemisia* (*tabaci* group) host records are erroneous as the result of misidentifications. Surveys of native whitefly to assess possible non-target host utilization by exotic *Eretmocerus* in the USA have produced no instances of introduced species attacking native hosts. Such surveys have yielded newly-described and additional undescribed species of *Eretmocerus*.
